# Lipase-catalyzed alcoholysis of butter for acid value reduction: a comparative study with lipase-catalyzed hydrolysis

**DOI:** 10.1016/j.fochx.2026.104753

**Published:** 2026-11-05

**Authors:** Xinyu Guo, Yunjie Lei, Weinong Zhang, Tao Yang, Zongyuan Wu, Jiangtao Feng, Yanxia Cong

**Affiliations:** a College of Food Science and Engineering, Wuhan Polytechnic University, Key Laboratory for Deep Processing of Major Grain and Oil, Ministry of Education, Hubei Key Laboratory for Processing and Transformation of Agricultural Products, Wuhan 430048, China; b Wuhan Qisidalebei Food Co. LTD., Wuhan 430062, China

**Keywords:** Butterfat, Flavor regulation, Lipase-catalyzed hydrolysis, Lipase-catalyzed alcoholysis, Esters

## Abstract

Lipase-catalyzed hydrolysis can improve butter flavor but may cause acid accumulation and undesirable taste. In this study, ethanol was employed as an acyl acceptor to establish an lipase-catalyzed alcoholysis system, and its effects on butter properties and volatile profiles were compared with lipase-catalyzed hydrolysis. After 72 h, lipase-catalyzed hydrolysis increased the acid value from 0.75 to 8.74 and 3.03 mg KOH·g^−1^ for Palatase 20,000 L and lipase MHA 10SD, whereas ethanol reduced it to 0.72 mg KOH·g^−1^ (91.8% reduction) and 0.85 mg KOH·g^−1^ (71.9% reduction). Volatile analysis showed that hydrolyzed butter was characterized by acids, ketones, and lactones, whereas alcoholyzed butter exhibited low acids and ester-dominated profile, with ethyl butyrate and ethyl octanoate as major esters. Lipase-catalyzed alcoholysis effectively reduced acid accumulation and shifted the flavor toward fruity and creamy profile, providing a promising strategy for producing high-quality flavored butter with improved sensory properties and industrial application potential.

## Highlights

• Enzymatic alcoholysis eliminates acids, reduces ketones and lactones.
• The flavor of enzymatically alcoholized butter is mainly dominated by esters.
• Esters in enzymatically alcoholized butter are dominated by ethyl butyrate and ethyl caprate.
• Palatase 20,000 L has higher esterification activity than Lipase MHA 10SD.

## Graphical abstract

**Figure:**
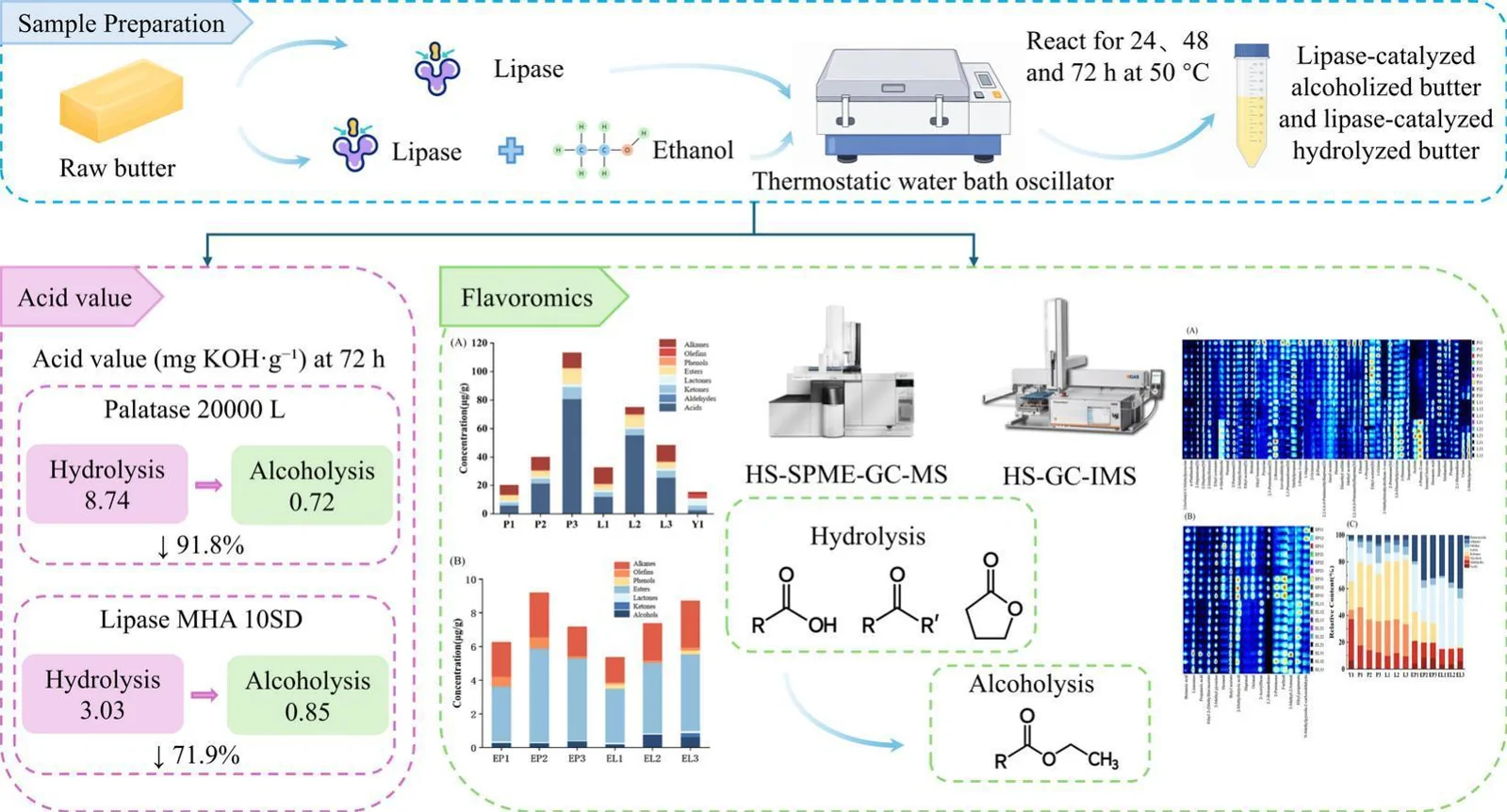

## 1 Introduction

As a flavor-enhancing ingredient, lipase-catalyzed hydrolyzed butter is highly favored in the food industry, particularly in baking, and its distinctive flavor profile significantly elevates product quality, thereby aligning with the current market demand for premium baked goods (Lubary et al., 2010). This ingredient is produced by lipase-catalyzed hydrolysis of butter using proteases or lipases. Its flavor profile stems from the targeted breakdown of milk fats and milk proteins during the lipase-catalyzed process (Ramon Tome Oliveira et al., 2025). Compared to conventional butter, it boasts a richer, more complex flavor—prominent dairy and cream aromas, complemented by subtle nutty and caramel notes, along with a full, rounded taste, mild acidity and a lingering finish (Wang & Xu, 2009; Zhang et al., 2016). Key flavor compounds in lipase-catalyzed hydrolyzed butter include short-chain fatty acids (SCFAs) and medium-chain fatty acids (MCFAs), which serve as the primary hydrolyzed components that deliver rich dairy aromas, enhance the creamy texture from milk fat, and intensify both the base and aftertaste (Wang et al., 2024; Zhang et al., 2021). Additionally, lactones (e.g., *γ*-decalactone) and methyl ketones (e.g., 2-heptanone, 2-nonanone) contribute distinct dairy-like aromas, the former adds a gentle sweetness, while the latter brings fruity and creamy freshness, together forming its complex flavor profile (Cong et al., 2025; Mu et al., 2024; Wang et al., 2024; Zhang et al., 2021).

Recent advances have increasingly focused on improving the flavor and sensory quality of butter through controlled lipase-catalyzed modification. Chen et al. (2021) developed a two-step hydrolysis-esterification strategy for milk fat, which increased desirable sweet, floral, and fruity esters while reducing odor-active fatty acids, thereby improving the overall flavor profile. More recently, Cong et al. (2025) demonstrated that different lipases produced distinct volatile profiles in enzyme-modified butter, with acids, lactones, and ketones serving as major contributors to flavor development, highlighting the importance of enzyme selection and hydrolysis control. In addition, Fan et al. (2025) combined lipase-catalyzed hydrolysis with *Lactococcus lactis* fermentation to produce cheese-flavored butter, further demonstrating the potential of lipase-catalyzed and microbial approaches for tailoring butter flavor. Despite these efforts, the technology still faces several challenges. Improper reaction control can cause excessive hydrolysis, leading to high free fatty acids (FFAs) content that results in rancid flavors and impairs product taste and stability (Cong et al., 2025). Additionally, enzyme preparations are relatively expensive, and their activity is easily affected by temperature, pH, water activity, and substrate concentration, all of which increase the complexity and expense of maintaining optimal reaction conditions (Ramon Tome Oliveira et al., 2025). Moreover, flavor compounds from existing lipase-catalyzed processes often lack sufficient variety and concentration, struggling to meet market demand for rich, intense, multi-layered aromas.

Esters, such as ethyl octanoate and ethyl butyrate, are important flavor compounds in butter. They can provide pleasant creamy and fruity notes, effectively enhancing the aroma complexity of butter (de Oliveira et al., 2019). However, current methods for esters production are limited, as chemically synthesized esters fail to replicate the stereochemical structure and flavor synergistic effects of natural ones and face consumer acceptance issues (Xu et al., 2020). While microbial fermentation alone suffers from low yields, poor stability, and environmental sensitivity (Panakkal et al., 2021). Ramon Tome Oliveira et al. (2025) noted in their 2018–2024 review that existing lipase-catalyzed esters synthesis primarily focuses on direct esterification of SCFAs and alcohols. For example, Radzi et al. (2011) and Martins et al. (2011) used immobilized lipases Lipozyme RM IM and Novozym 435 to synthesize pineapple-flavored butyl acetate from acetic acid and butanol. Beyond direct esterification, FFAs released by lipase-catalyzed hydrolysis serve as valuable substrates for esters biosynthesis in alcohol-triglyceride systems. Chen et al. (2021) demonstrated that Lipozyme TL IM can be used to sequentially catalyze goat milk fat by hydrolyzing it to release FFAs, then esterifying it to reconstitute flavor, which raised sweet, floral, and fruity esters to over 65% of total volatile components while reducing undesirable fatty acids by 75%. Enzyme-catalyzed esterification is also effective in modifying flavors of coconut milk and milk fat. Palatase 20,000 L catalyzes transesterification of coconut cream with fusel oil to produce aromatic octanoic acid esters (e.g., ethyl octanoate), which enhance creamy flavor (Sun et al., 2012). Similarly, lipases like Lipozyme TL, Lipozyme RM, and Novozym 435 can convert SCFAs in milk fats into creamy ethyl caprylate and fruity ethyl butyrate, with Novozym 435 exhibiting the highest activity and selectivity across various reaction systems (Lubary et al., 2009). Among various alcohol-based acyl acceptors, ethanol is an attractive acyl acceptor for food-related lipase-catalyzed alcoholysis because of its suitability for food applications and favorable safety profile when used at appropriate levels. Compared with methanol, ethanol has been reported to exert a weaker inhibitory effect on certain lipases, which may facilitate lipase-catalyzed alcoholysis under appropriate reaction conditions (Sasso et al., 2015). Moreover, ethanolysis can generate short-chain fatty acid ethyl esters, including ethyl butyrate and ethyl octanoate, which are important flavor compounds (Lubary et al., 2009). Thus, ethanol provides a practical route for converting lipase-released FFAs into volatile flavor esters while reducing acid accumulation.

This study addresses the issue of excessive FFAs generated during lipase-catalyzed hydrolysis of butter. By introducing ethanol as an acyl acceptor in the lipase-catalyzed stage, a lipase-catalyzed alcoholysis system is established. In this system, lipase directly catalyzes the alcoholysis of butter, converting lipids into fatty acid ethyl esters. This process not only effectively reduces FFAs content by shifting the reaction toward ester formation, but also imparts richer, more natural flavors to the butter through the generated esters. Consequently, it achieves the dual objectives of regulating acid value (AV) while enhancing the overall flavor quality of lipase-catalyzed hydrolyzed butter.

## 2 Materials and methods

### 2.1 Chemicals and materials

This experiment utilized Anchor original flavor butter (New Zealand) as the raw material, stored at 4 °C. Its main components are fat, protein, lactose, and water. Palatase 20,000 L was obtained from Novozymes (Bagsvaerd, Denmark) and Lipase MHA 10SD was purchased at Amano Enzyme (Nagoya, Japan). The C7–C40 n-saturated alkanes mixture for volatile compound identification, 37 fatty acid methyl esters mixture and internal standard (IS) 2-methyl-3-heptanone, were obtained from Anpel (Shanghai, China). Normal ketones (2-butanone, 2-pentanone, 2-hexanone, 2-heptanone, 2-octanone, 2-nonanone) were purchased from Hanon (Shandong, China). All of anhydrous diethyl ether, isopropyl alcohol, sodium hydroxide, and anhydrous ethanol were of analytical grade, while n-hexane was chromatographic grade, all purchased from Sinopharm (Shanghai, China). Boron trifluoride-methanol was analytical grade and obtained from Aladdin (Shanghai, China). Methanol of chromatographic grade was sourced from Kemiou (Tianjin, China).

### 2.2 Preparation of lipase-catalyzed alcoholized butter and lipase-catalyzed hydrolyzed butter

50 g of butter was weighed and melted at 80 °C for 5 min, then allowed them to cool. Following the optimal conditions recommended by the manufacturer, lipase was directly added into the melted butter at an enzyme activity of 2 U/g substrate. For the ethanol-containing treatments, ethanol was added at 10% (w/w) relative to the butter. The reactions were conducted in sealed centrifuge tubes with air as the headspace atmosphere and incubated in a water-bath shaker at 50 °C for 200 rpm. Reactions were conducted using Palatase 20,000 L without ethanol for 24 h (P1), 48 h (P2), and 72 h (P3), and with 10% (w/w) ethanol for 24 h (EP1), 48 h (EP2), and 72 h (EP3), using Lipase MHA 10SD without ethanol for 24 h (L1), 48 h (L2), and 72 h (L3), and with 10% (w/w) ethanol for 24 h (EL1), 48 h (EL2), and 72 h (EL3). After the designated reaction time, each sample was heated at 85 °C for 15 min to inactivate the enzyme, cooled to room temperature, labeled with the above codes, and stored at −20 °C for future use.

### 2.3 Physicochemical composition analysis

#### 2.3.1 AV analysis

The AV was measured following the method of Cong et al. (2025) with minor adjustments. Briefly, 10 g of butter was weighed into a 50 mL centrifuge tube and melted at 45 °C. Then the sample was centrifuged at 6796×*g* for 5 min at 25 °C, and the supernatant was retained as the test solution. The AV was determined by titration with standardized KOH solution (0.01958 mol/L) and using phenolphthalein as the indicator. The standardization of the KOH solution was performed according to the Chinese National Standard GB/T601—2016 (2016), using potassium hydrogen phthalate as the primary standard and phenolphthalein as the indicator.

#### 2.3.2 Solid fat content (SFC) analysis

SFC during sample crystallization was measured by an NMI20-040V-I nuclear magnetic resonance instrument (Suzhou Niumag Analytical Instrument Co., Ltd., China), referencing the method of Cong et al. (2025). Briefly, 6 mL of butter was transferred into a standard solid fat tube (inner diameter of 0.8 cm), forming a liquid column of approximately 4 cm. The sample was melted at 80 °C for 30 min to erase solid fat crystallization memory, then equilibrated at 0 °C for 1 h. Subsequently, the sample was incubated sequentially in a water bath at each target temperature (10, 20, 25, 30, and 35 °C) for 30 min, then measured SFC of them.

### 2.4 Fatty acid proportion analysis

The fatty acid proportion of butter was analyzed by gas chromatography (GC) using an Agilent 7890A gas chromatograph (Agilent Technologies Inc., USA), following the method of Tene et al. (2024). Briefly, 0.1 g of butter was weighed into a methyl esterification tube, vortex-mixed with 2 mL of 0.5 mol/L sodium hydroxide-methanol solution, and incubated at 65 °C for 30 min. Then, 2 mL of boron trifluoride-methanol solution was added and the reaction was continued at 65 °C for 3 min. After cooling, 2 mL of n-hexane was added for extraction by shaking, the mixture was allowed to separate, and the upper layer was collected for GC analysis. A Supelco SP-2560 capillary GC column (100 m × 0.25 mm × 0.2 μm, Supelco, USA) was used with high-purity nitrogen as the carrier gas. The GC was operated in constant-pressure mode at 31.31 psi with a split ratio of 20:1 and an injection volume of 1 μL. The injector and flame ionization detector (FID, Agilent Technologies Inc., USA) temperatures were both maintained at 250 °C. The column temperature program initially held at 100 °C for 4 min, then increased to 230 °C at 3 °C/min and held for 20 min, with a total analysis time of 67.33 min.

### 2.5 Volatile components analysis

#### 2.5.1 Headspace solid-phase microextraction gas chromatography–mass spectrometry (HS-SPME-GC–MS) analysis

Volatile aroma components in butter were extracted by headspace solid-phase microextraction (HS-SPME), with reference to Cong et al. (2025). Briefly, 2.0 g of butter was weighed into a 20 mL headspace vial, then 10 μL of 2-methyl-3-heptanone working solution (0.408 mg/mL), prepared by 2000-fold dilution of the pure compound (density = 0.816 g/mL), was added as the IS. The sample was then thoroughly mixed at 500 rpm for 3 min to ensure uniform distribution of the IS and equilibrated at 80 °C for 20 min before HS-SPME extraction. The headspace was extracted with a SPME fiber (CAR/PDMS, 75 μm), which was then desorbed in the GC injection port at 250 °C for 5 min. GC–MS analysis was performed using an Agilent 7890A-5975C gas chromatography-mass spectrometry system (Agilent Technologies Inc., USA) equipped with an HP-5 capillary column (30 m × 0.32 mm × 0.25 μm, Agilent Technologies Inc., USA). Helium (purity ≥99.999%) was used as the carrier gas at a flow rate of 1.8 mL/min under splitless conditions. The oven temperature program held initially at 50 °C for 3 min, then increased to 250 °C at 6 °C/min and held for 5 min. Electron ionization mode was used for the mass spectrometer at 70 eV, with an ion source temperature of 200 °C, interface temperature of 250 °C, emission current of 200 μA, and a mass scan range of 30–402 *m*/*z*. Identification of volatile compounds was performed by comparison of their mass spectra with the NIST.11 library (confidence level > 80%). Retention indices (RI) were calculated from the retention times of C7–C40 n-alkanes under the same conditions and served to confirm the library search results. The concentration of each compound was calculated by comparing its peak area with that of the IS using formula as follows, as described by Yang et al. (2024).

$$C_{i}=\frac{S_{i}}{S_{A}}\times C_{A}\times \frac{V_{A}}{m_{i}}\times n_{o/i}$$

where C_i_ is the concentration of the unknown compound (*μ*g/g); C_A_ is the mass concentration of 2-methyl-3-heptanone (g/mL); S_i_ is the peak area of the unknown compound; S_A_ is the peak area of 2-methyl-3-heptanone; V_A_ is the volume of the diluent (mL); n_o/i_ is the ratio of the IS volume to the diluent volume in the mixture; m_i_ is the mass of the sample before pretreatment (g).

#### 2.5.2 Headspace gas chromatography-ion mobility spectrometry (HS-GC-IMS) analysis

Volatile organic compounds (VOCs) in butter were analyzed by HS-GC-IMS (FlavourSpec®, G.A.S., Germany) following the method of Cong et al. (2025). Briefly, 1 g of butter was weighed into a headspace vial and incubated for 10 min at 80 °C with shaking at 500 rpm. Headspace injection parameters were set with an injection volume of 500 μL, injection temperature of 85 °C. Chromatographic separation was carried out on an MXT-5 column (15 m × 0.53 mm × 1 μm, Restek Corporation, USA), with column temperature set at 60 °C and analysis time of 21 min. The GC conditions were set with an initial flow rate of 1 mL/min, increasing to 2 mL/min over 2 min, then to 5 mL/min at 2 mL/min, linearly rising to 15 mL/min over the next 6 min, and finally to 150 mL/min over the last 10 min. IMS conditions were set with a *β*-ray (^3^H) radiation source, drift gas of high-purity nitrogen (purity ≥99.999%) at a flow rate of 150 mL/min, and drift tube temperature of 45 °C. For qualitative analysis, C4–C9 normal ketones severd as external standards for RI calculation. Identification of volatile compounds was performed by comparing RI and drift time with those in the standard IMS database, and their relative content were calculated based on peak intensities.

#### 2.5.3 Relative odor activity value (ROAV) analysis

The ROAV was calculate following the method of Xu et al. (2022). The compound contributing most significantly to the overall aroma profile was assigne ROAV_stan_ of 100. The ROAV for the remaining components (x) was calculated according to formula as follows:

$${\mathrm{ROAV}}_{x}=\frac{100\times C{\%}_{x}\times T_{\mathrm{stan}}}{C{\%}_{\mathrm{stan}}\times T_{x}}$$

where C%_x_ and T_x_ is the relative content (%) and odor threshold (mg/kg) of each volatile compound; C%_stan_ and T_stan_ is the relative content (%) and odor threshold (mg/kg) of the compound contributing most significantly to the sample's flavor.

### 2.6 Data statistics and analysis

All samples were measured in triplicate, and the results were expressed as mean ± standard deviation. Statistical analyses were performed using SPSS 19.0 (IBM Corp., Armonk, NY, USA). Significance analysis between two groups was performed using Welch's independent-samples *t*-test (two-tailed), whereas multiple groups were assessed using one-way analysis of variance (ANOVA), followed by the Waller–Duncan multiple range test. A *p*-value of less than 0.05 was considered statistically significant. Bar charts were generated using Origin 2024 (OriginLab Corporation, Northampton, MA, USA). Partial least squares-discriminant analysis (PLS-DA), variable importance in projection (VIP) analysis, principal component analysis (PCA), and heatmap visualization were performed using MetaboAnalyst 6.0 (McGill University, Montreal, QC, Canada). 200-permutation tests were performed on the Metware Cloud platform (Wuhan Metware Biotechnology Co., Ltd., Wuhan, China).

## 3 Results and discussion

### 3.1 Physicochemical composition analysis

#### 3.1.1 AV analysis

As shown in Table 1, the addition of ethanol significantly altered the AV during lipase-catalyzed butter hydrolysis. For the lipase-catalyzed hydrolyzed butter (P and L series), AV increased markedly with reaction time from 24 to 72 h. Specifically, AV in the P series increased from 2.71 to 8.74 mg KOH·g^−1^, while that in the L series increased from 2.01 to 3.03 mg KOH·g^−1^. In comparison, the AV of raw butter (Y1) was only 0.75 mg KOH·g-1. This indicated that lipase continuously catalyzed triglyceride hydrolysis, leading to substantial FFAs accumulation. However, for the lipase-catalyzed alcoholized butter (EP and EL series), AV was dramatically lower than those in the P and L series. Notably, AV in EP series remained consistently low throughout the reaction (0.75–0.72 mg KOH·g^−1^), and EL series decreased continuously with prolonged reaction time, from 1.13 (24 h) to 0.85 (72 h) mg KOH·g^−1^. This discrepancy can be ascribed to the dual role of ethanol. Ethanol may suppress the hydrolytic activity of lipase by altering its conformation (Rigos et al., 2006), while simultaneously acting as a nucleophile that reacts with FFAs to produce fatty acid ethyl esters, thereby limiting the accumulation of FFAs. The occurrence of esterification also shifted the hydrolysis equilibrium toward the reverse direction, in accordance with Le Chatelier's principle (Javed et al., 2019). Moreover, the distinct behaviors of the two lipases during alcoholysis reflected their inherent substrate specificities. Ethanol addition significantly reduced the AV. In the Palatase 20,000 L group, the AV of the EP series decreased by 72.3%–91.8% compared with the P series. In the Lipase MHA 10SD group, the reduction was smaller, with the EL series showing a decrease of 43.3%–71.9% relative to the L series. Throughout the reaction, AV for the EP series were consistently lower than those for the EL series. These results suggest that Palatase 20,000 L may have favored the formation of fatty acid ethyl esters from FFAs, whereas Lipase MHA 10SD showed comparatively lower levels of hydrolysis and ester formation under the present conditions (Zhang et al., 2016).

**Table 1:** AV of raw, lipase-catalyzed hydrolyzed and lipase-catalyzed alcoholized butter.

Sample name	AV (mg KOH/g)	Sample name	AV (mg KOH/g)
Y1	0.75 ± 0.02^f^	EP1	0.75 ± 0.01^d^
P1	2.71 ± 0.02^d^	EP2	0.73 ± 0.01^d^
P2	4.84 ± 0.04^b^	EP3	0.72 ± 0.02^d^
P3	8.74 ± 0.21^a^	EL1	1.13 ± 0.03^a^
L1	2.01 ± 0.02^e^	EL2	0.93 ± 0.02^b^
L2	2.91 ± 0.01^cd^	EL3	0.85 ± 0.01^c^
L3	3.03 ± 0.02^c^		

The results were expressed as mean ± standard deviation (*n* = 3). Mean values with different lowercase letters in the same column are significantly different at *p* < 0.05. Y1: raw butter; P1-P3: hydrolysis by Palatase 20,000 L for 24, 48, 72 h; L1-L3: hydrolysis by Lipase MHA 10SD for 24, 48, 72 h; EP1-EP3: alcoholysis by Palatase 20,000 L for 24, 48, 72 h; EL1-EL3: alcoholysis by Lipase MHA 10SD for 24, 48, 72 h.

#### 3.1.2 SFC analysis

Changes in the SFC of butter during the reaction are presented in Fig. 1. Overall, the SFC of all treated samples decreased with increasing temperature. Both lipase-catalyzed hydrolysis and lipase-catalyzed alcoholysis markedly modified the SFC-temperature profile compared with the Y1. The SFC of Y1 decreased gradually from 49.84% at 0 °C to 0.60% at 35 °C. In contrast, all treated samples displayed steeper melting curves. The SFC of Y1 decreased from 49.84% at 0 °C to 0.60% at 35 °C, whereas the treated samples exhibited steeper melting profiles, and reached 0% at 30 or 35 °C. The gradual decrease in SFC of Y1 may be associated with the broad melting range of its native triacylglycerols, whereas the steeper declines in treated samples may be related to the changes in lipid composition, as FFAs released during hydrolysis and fatty acid ethyl esters formed during alcoholysis may have altered fat crystallization and melting behavior, potentially contributing to the observed SFC changes (Soleimanian et al., 2023). At 0 °C, the SFC of treated samples ranged from 50.04% to 63.07%, with the L series showing the highest values. As temperature increased, the differences became more pronounced. At 20 °C, the P and EP series showed lower SFC values (6.69%–9.87% and 7.49%–11.60%) than the L and EL series (16.04%–16.73% and 13.47%–14.87%). This trend persisted at 25 °C, and at 30 °C, the P and EP series had completely melted, whereas the L and EL series still retained a small amount of solid fat. This may suggest that the effects of enzymatic treatment on melting behavior depended on the lipase and reaction system.

**Fig. 1:**
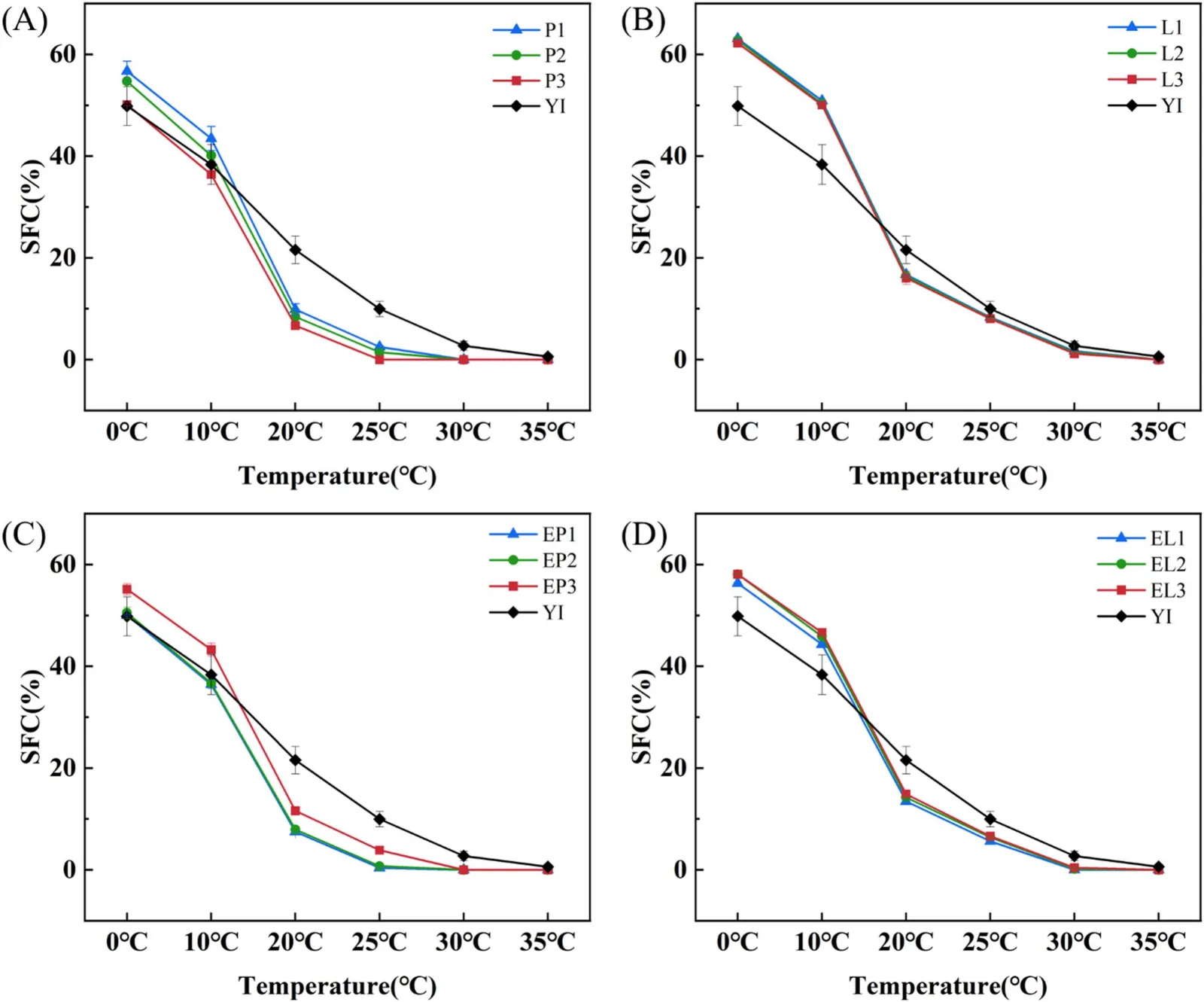
SFC curves of raw, lipase-catalyzed hydrolyzed and lipase-catalyzed alcoholized butter. (A) Group of P; (B) Group of L; (C) Group of EP; (D) Group of EL.

Compared with the lipase-catalyzed hydrolyzed samples, the lipase-catalyzed alcoholyzed samples generally exhibited lower SFC values. This difference may be explained by the distinct lipid compositions of the two systems. The P and L series contained relatively high levels of FFAs, and their ester products were mainly fatty acid methyl esters, whereas the EP and EL series contained lower levels of FFAs, with fatty acid ethyl esters as the predominant ester products. Fatty acid ethyl esters generally have lower melting points than the corresponding methyl esters, and esters exhibit lower melting points than FFAs, the different proportions of FFAs and esters may have contributed to the lower SFC observed in the EP and EL series (Ginsburg & Jiménez-Flores, 2024). In addition, ethanol may induce structural changes in lipases, reducing their catalytic activity, thereby limiting FFAs formation (Endo, 2024; Rigos et al., 2006). As a nucleophile, ethanol can react with FFAs to form fatty acid ethyl esters (Fang et al., 2025). Ethanol may also competitively inhibit ester bond hydrolysis while promoting the accumulation of fatty acid ethyl esters. The synergistic effect of increased ethyl esters with reduced methyl esters and FFAs contributed to the lower SFC in lipase-catalyzed alcoholyzed butter. Moreover, SFC for the L and EL series were consistently higher than those for the P and EP series across all measured temperatures. This trend closely correlated with the amount of FFAs generated in each system. FFAs disrupted the ordered packing of glyceride molecules,thereby hindering the formation and stabilization of high-melting crystal networks. Additionally, FFAs showed poor crystallization compatibility and limited capacity to integrate into the solid fat crystal structure, thus lowering the overall solid fat proportion (Monie et al., 2023).

### 3.2 Fatty acid proportion analysis

Fatty acid proportion is a key index for evaluating lipid quality. Fatty acid profiles of hydrolyzed and alcoholyzed butter are shown in Table S1. In total, 37 fatty acids were identified by GC, including FFAs and fatty acyl groups in glycerides. Palmitic acid (C16:0) and oleic acid (C18:1) were the dominant fatty acids in all samples, consistent with the finding of Demir et al. (2024). Minor differences in fatty acid proportion were observed among samples, which may be related to differences in the extent of lipid modification under the two enzyme treatments. Previous studies have reported that some lipases exhibit different substrate preferences toward fatty acids of different chain lengths (Acquistapace et al., 2019). Specifically, the SCFAs content in the P and EP series increased markedly and was considerably higher than those in Y1, with butyric acid (C4:0) showing the most prominent elevation. The MCFAs proportion in the P and EP series reached up to 10.12% (EP2), far exceeding the 7.72% in Y1, primarily due to the increased levels of capric acid (C10:0) and lauric acid (C12:0). In comparison, the SCFAs contents in the L and EL series were only slightly higher than Y1 and significantly lower than the P and EP series. Meanwhile, the MCFAs contents in the L and EL series (7.54%–7.72%) remained close to that of Y1. These differences suggest that Palatase 20,000 L may possess higher hydrolytic activity toward butter fats than Lipase MHA 10SD, which was also reported for enzyme-modified butter by Cong et al. (2025). In addition, the proportions of monounsaturated fatty acids (MUFAs) also varied among the samples. C18:1 was the main MUFA. Its proportion in the P and EP series (16.21%–17.94%) was lower than those in Y1 (18.28%) and the L, EL series (17.81%–18.93%). The regioselectivity and fatty acid specificity of lipases may drive the preferential conversion of C18:1 during reaction, thereby reducing its relative content in the residual glycerides (Chen et al., 2023). Therefore, we hypothesized that this decrease may be related to the differential modification of MUFAs by Palatase 20,000 L. Among saturated fatty acids (SFAs), C16:0 was the most abundant. Its content was higher in the L and EL series. Although the substrate specificity and regioselectivity of lipases may contribute to these differences, the relative content of C16:0 may also result from changes in the relative content of other fatty acids, particularly unsaturated fatty acids (Yang et al., 2021).

### 3.3 Volatile components analysis

#### 3.3.1 HS-SPME-GC–MS analysis

VOCs in lipase-catalyzed alcoholized butter, lipase-catalyzed hydrolyzed butter, and raw butter were qualitatively and quantitatively analyzed by HS-SPME-GC–MS (Table 2 and Table 3). A total of 22 VOCs belonging to six classes were detected in Y1, including 4 acids, 2 aldehydes, 3 ketones, 4 lactones, 3 alkenes, and 6 alkanes. Meanwhile, 43 VOCs across seven classes were identified in the P and L series, including 6 acids, 5 ketones, 5 lactones, 8 esters, 1 phenol, 1 alkene, and 17 alkanes. For the EP and EL series, 32 VOCs spanning seven classes were detected, with 6 alcohols, 1 ketone, 1 lactone, 11 esters, 1 phenol, 2 alkenes, and 10 alkanes. These results indicate that lipase-catalyzed treatment markedly altered the volatile composition of butter. Lipase-catalyzed hydrolysis increased the diversity of acids, ketones, lactones, and esters compared with raw butter Y1, potentially owing to the release of FFAs from triglycerides and their subsequent conversion into various volatile compounds (Cong et al., 2025). In contrast, ethanol addition shifted the volatile profile toward esters and reduced the total number of detected volatile compounds in the EP and EL series. Ethanol altered the VOCs possibly through two main mechanisms. It directly consumed acids by esterification to form new esters, and suppressed side reactions (e.g., oxidation, cleavage) that generate undesirable flavor compounds, ultimately reducing the total number of detected VOCs in EP and EL series (Fang et al., 2025).

**Table 2:** HS-SPME-GC-MS integration parameters of VOCs in raw and lipase-catalyzed hydrolyzed butter.

No.	Compounds	CAS	Concentration (*μ*g/g)						
			Y1	P1	P2	P3	L1	L2	L3
	**ACIDS**		**1.96** **±** **0.34**^**f**^	**5.69** **±** **0.57**^**ef**^	**21.11** **±** **7.90**^**cd**^	**80.66** **±** **2.77**^**a**^	**11.87** **±** **2.46**^**de**^	**55.26** **±** **0.70**^**b**^	**25.42** **±** **5.58**^**c**^
1	Butanoic acid	000107-92-6	n.d.^1^	1.41 ± 0.23^d^	4.82 ± 1.32^c^	15.13 ± 0.38^a^	2.42 ± 0.002^d^	7.28 ± 0.63^b^	6.09 ± 0.59^bc^
2	Hexanoic acid	000142-62-1	0.59 ± 0.11^d^	0.66 ± 0.37^d^	9.05 ± 4.46^c^	32.95 ± 0.89^a^	4.73 ± 0.8^cd^	18.33 ± 0.79^b^	8.98 ± 1.10^c^
3	Octanoic acid	000124-07-2	0.59 ± 0.04^d^	2.93 ± 0.22^cd^	4.81 ± 3.03^cd^	15.57 ± 1.35^b^	6.00 ± 0.001^cd^	22.72 ± 1.60^a^	5.76 ± 2.84^c^
4	n-Decanoic acid	000334-48-5	0.58 ± 0.11^c^	0.68 ± 0.10^c^	2.22 ± 1.03^bc^	13.43 ± 1.44^a^	3.28 ± 0.41^bc^	6.46 ± 0.28^b^	3.22 ± 1.38^bc^
5	Nonanoic acid	000112-05-0	n.d.	n.d.	0.30 ± 0.06^bc^	0.90 ± 0.18^a^	0.39 ± 0.04^b^	0.73 ± 0.19^a^	0.08 ± 0.05^c^
6	Dodecanoic acid	000143-07-7	0.12 ± 0.05^c^	n.d.	n.d.	2.67 ± 1.18^a^	1.05 ± 0.49^bc^	2.23 ± 0.78^ab^	0.63 ± 0.24^c^
	**ALDEHYDES**		**0.75** **±** **0.05**^**a**^	**n.d.**	**n.d.**	**n.d.**	**n.d.**	**n.d.**	**n.d.**
1	Phenylacetaldehyde	000122-78-1	0.55 ± 0.02^a^	n.d.	n.d.	n.d.	n.d.	n.d.	n.d.
2	Nonanal	000124-19-6	0.20 ± 0.06^a^	n.d.	n.d.	n.d.	n.d.	n.d.	n.d.
	**ALCOHOLS**		**n.d.**	**n.d.**	**n.d.**	**n.d.**	**n.d.**	**n.d.**	**n.d.**
1	Ethanol	000064-17-5	n.d.	n.d.	n.d.	n.d.	n.d.	n.d.	n.d.
2	3-Methyl-2-hexanol	002313-65-7	n.d.	n.d.	n.d.	n.d.	n.d.	n.d.	n.d.
3	5-Methyl-2-hexanol	000627-59-8	n.d.	n.d.	n.d.	n.d.	n.d.	n.d.	n.d.
4	2-Octanol	000123-96-6	n.d.	n.d.	n.d.	n.d.	n.d.	n.d.	n.d.
5	5-Methyl-2-heptanol	054630-50-1	n.d.	n.d.	n.d.	n.d.	n.d.	n.d.	n.d.
6	6-Methyl-2-heptanol	004730-22-7	n.d.	n.d.	n.d.	n.d.	n.d.	n.d.	n.d.
	**KETONES**		**3.08** **±** **0.43**^**bc**^	**2.07** **±** **0.40**^**c**^	**3.43** **±** **1.17**^**bc**^	**8.56** **±** **0.82**^**a**^	**3.31** **±** **0.87**^**bc**^	**4.42** **±** **0.34**^**b**^	**4.66** **±** **1.60**^**b**^
1	2-Heptanone	000110-43-0	0.90 ± 0.22^bc^	0.79 ± 0.17^bc^	0.97 ± 0.44^bc^	1.58 ± 0.05^a^	0.52 ± 0.18^c^	1.23 ± 0.18^ab^	1.20 ± 0.17^ab^
2	2-Nonanone	000821-55-6	1.40 ± 0.12^bc^	0.57 ± 0.10^c^	1.01 ± 0.30^bc^	2.65 ± 0.11^a^	0.90 ± 0.47^bc^	1.75 ± 0.06^ab^	2.20 ± 0.34^bc^
3	2-Undecanone	000112-12-9	0.78 ± 0.1^b^	0.37 ± 0.07^b^	0.62 ± 0.19^b^	3.32 ± 1.01^a^	0.49 ± 0.11^b^	0.90 ± 0.04^b^	1.24 ± 0.20^b^
4	2-Tridecanone	000593-08-8	n.d.	0.22 ± 0.07^b^	0.80 ± 0.07^a^	0.70 ± 0.12^a^	0.39 ± 0.06^b^	0.60 ± 0.14^a^	n.d.
5	2-Pentadecanone	002345-28-0	n.d.	0.34 ± 0.06^b^	0.63 ± 0.21^a^	0.21 ± 0.01^bc^	0.70 ± 0.10^a^	0.14 ± 0.02^bc^	0.15 ± 0.03^bc^
	**LACTONES**		**5.03** **±** **0.86**^**a**^	**1.25** **±** **0.23**^**c**^	**1.62** **±** **0.30**^**bc**^	**2.37** **±** **0.13**^**b**^	**1.72** **±** **0.24**^**bc**^	**1.52** **±** **0.17**^**bc**^	**1.91** **±** **0.28**^**bc**^
1	*δ*-Octalactone	000698-76-0	0.67 ± 0.07^bc^	0.54 ± 0.09^c^	0.56 ± 0.19^bc^	1.42 ± 0.07^a^	0.81 ± 0.14^bc^	0.87 ± 0.08^b^	0.87 ± 0.20^b^
2	*δ*-Decalactone	000705-86-2	2.86 ± 0.48^a^	n.d.	n.d.	n.d.	n.d.	n.d.	n.d.
3	*γ*-6-(*Z*)-Dodecenolactone	018679-18-0	n.d.	n.d.	0.49 ± 0.35^a^	n.d.	n.d.	n.d.	0.25 ± 0.09^ab^
4	*γ*-Undecalactone	000104-67-6	n.d.	0.50 ± 0.08^ab^	0.31 ± 0.23^abc^	0.30 ± 0.02^abc^	0.60 ± 0.06^a^	0.16 ± 0.03^bc^	0.38 ± 0.34^abc^
5	*δ*-Dodecalactone	000713-95-1	1.23 ± 0.12^a^	0.21 ± 0.07^d^	0.26 ± 0.09^d^	0.66 ± 0.03^b^	0.32 ± 0.06^cd^	0.49 ± 0.10^bc^	0.41 ± 0.08^cd^
6	*δ*-Tetradecalactone	002721-22-4	0.27 ± 0.20^a^	n.d.	n.d.	n.d.	n.d.	n.d.	n.d.
	**ESTERS**		**n.d.**	**3.91** **±** **0.41**^**c**^	**3.88** **±** **0.49**^**c**^	**10.68** **±** **0.63**^**a**^	**3.69** **±** **0.47**^**c**^	**8.32** **±** **1.37**^**b**^	**4.40** **±** **0.67**^**c**^
1	Hexanoic acid, methyl ester	000106-70-7	n.d.	0.47 ± 0.34^ab^	0.31 ± 0.21^bc^	0.79 ± 0.01^a^	0.29 ± 0.17^bc^	0.77 ± 0.03^a^	0.39 ± 0.06^abc^


No.	Compounds	CAS	Concentration (*μ*g/g)						
			Y1	P1	P2	P3	L1	L2	L3
2	Octanoic acid, methyl ester	000111-11-5	n.d.	0.59 ± 0.09^e^	0.94 ± 0.34^de^	2.32 ± 0.11^b^	1.06 ± 0.14^d^	3.25 ± 0.21^a^	1.65 ± 0.23^c^
3	Methoxyacetic acid, pentyl ester	168920-35-2	n.d.	n.d.	n.d.	n.d.	n.d.	n.d.	n.d.
4	Methoxyacetic acid, decyl ester	259141-02-1	n.d.	n.d.	n.d.	n.d.	n.d.	n.d.	n.d.
5	Methoxyacetic acid, 2-pentyl ester	1000282-69-2	n.d.	n.d.	n.d.	n.d.	n.d.	n.d.	n.d.
6	Oxalic acid, 6-ethyloct-3-yl hexyl ester	1000309-34-4	n.d.	0.57 ± 0.31^ab^	0.47 ± 0.36^abc^	0.83 ± 0.04^a^	0.57 ± 0.32^ab^	0.16 ± 0.03^bc^	0.33 ± 0.07^abc^
7	Octanoic acid, ethyl ester	000106-32-1	n.d.	n.d.	n.d.	n.d.	n.d.	n.d.	n.d.
8	Decanoic acid, methyl ester	000110-42-9	n.d.	0.38 ± 0.08^de^	0.74 ± 0.31^cd^	3.08 ± 0.19^a^	0.64 ± 0.01^d^	1.72 ± 0.20^b^	1.16 ± 0.18^c^
9	Decanoic acid, ethyl ester	000110-38-3	n.d.	n.d.	n.d.	n.d.	n.d.	n.d.	n.d.
10	Dodecanoic acid, methyl ester	000111-82-0	n.d.	0.42 ± 0.33^b^	0.27 ± 0.11^b^	1.49 ± 0.20^a^	0.56 ± 0.08^b^	1.23 ± 0.47^a^	0.41 ± 0.05^b^
11	Dodecanoic acid, ethyl ester	000106-33-2	n.d.	n.d.	n.d.	n.d.	n.d.	n.d.	n.d.
12	Methyl tetradecanoate	000124-10-7	n.d.	0.65 ± 0.17^a^	0.27 ± 0.14^bcd^	0.48 ± 0.11^abc^	0.25 ± 0.04^bcd^	0.57 ± 0.31^ab^	0.15 ± 0.02^cd^
13	Methoxyacetic acid, 2-pentadecyl ester	1000282-05-1	n.d.	n.d.	n.d.	n.d.	n.d.	n.d.	n.d.
14	Methoxyacetic acid, octyl ester	029267-10-5	n.d.	n.d.	n.d.	n.d.	n.d.	n.d.	n.d.
15	Methoxyacetic acid, 3-tetradecyl ester	1000282-04-9	n.d.	n.d.	n.d.	n.d.	n.d.	n.d.	n.d.
16	Methoxyacetic acid, 3-tridecyl ester	1000282-04-6	n.d.	n.d.	n.d.	n.d.	n.d.	n.d.	n.d.
17	Acetic acid, 3,7,11,15-tetramethyl-hexadecyl ester	1000193-63-0	n.d.	0.27 ± 0.02^bc^	0.36 ± 0.12^b^	1.39 ± 0.33^a^	0.13 ± 0.01^bc^	0.23 ± 0.05^bc^	0.17 ± 0.04^bc^
18	Hexadecanoic acid, methyl ester	000112-39-0	n.d.	0.58 ± 0.17^a^	0.52 ± 0.35^ab^	0.31 ± 0.04^abc^	0.19 ± 0.03^abc^	0.40 ± 0.17^abc^	0.14 ± 0.03^bc^


No.	Compounds	CAS	Concentration (*μ*g/g)						
			Y1	P1	P2	P3	L1	L2	L3
19	Octadecanoic acid, ethyl ester	000111-61-5	n.d.	n.d.	n.d.	n.d.	n.d.	n.d.	n.d.
	**PHENOLS**		**n.d.**	**0.29** **±** **0.04**^**a**^	**0.43** **±** **0.15**^**a**^	**0.15** **±** **0.01**^**a**^	**0.41** **±** **0.01**^**a**^	**0.43** **±** **0.40**^**a**^	**0.37** **±** **0.33**^**a**^
1	2,4-Bis(1,1-dimethylethyl)-phenol	000096-76-4	n.d.	0.29 ± 0.04^a^	0.43 ± 0.15^a^	0.15 ± 0.01^a^	0.41 ± 0.01^a^	0.43 ± 0.40^a^	0.37 ± 0.33^a^
	**OLEFINS**		**3.13** **±** **0.24**^**a**^	**0.39** **±** **0.03**^**bc**^	**0.53** **±** **0.20**^**bc**^	**0.15** **±** **0.01**^**c**^	**0.50** **±** **0.05**^**bc**^	**0.56** **±** **0.31**^**bc**^	**0.77** **±** **0.15**^**b**^
1	8-Heptadecene	002579-04-6	1.91 ± 0.11^a^	n.d.	n.d.	n.d.	n.d.	n.d.	n.d.
2	2,6,10,14-Tetramethyl-2-hexadecene	056554-34-8	0.96 ± 0.10^a^	n.d.	n.d.	n.d.	n.d.	n.d.	n.d.
3	2-Hexadecene, 3,7,11,15-tetramethyl-, [R-[R*,R*-(E)]]-	014237-73-1	0.26 ± 0.03^bc^	0.39 ± 0.03^bc^	0.53 ± 0.20^ab^	0.15 ± 0.01^c^	0.50 ± 0.05^abc^	0.56 ± 0.31^ab^	0.77 ± 0.15^a^
	**ALKANES**		**1.45** **±** **0.16**^**c**^	**6.58** **±** **0.68**^**ab**^	**9.17** **±** **2.37**^**ab**^	**10.88** **±** **0.39**^**a**^	**11.14** **±** **3.84**^**a**^	**4.71** **±** **0.21**^**bc**^	**11.01** **±** **2.39**^**a**^
1	2,4-Dimethyl-heptane	002213-23-2	n.d.	n.d.	n.d.	n.d.	n.d.	n.d.	n.d.
2	4-Ethyl-decane	001636-44-8	n.d.	0.60 ± 0.13^b^	0.88 ± 0.35^ab^	1.27 ± 0.16^a^	0.41 ± 0.14^bc^	0.52 ± 0.04^b^	1.34 ± 0.27^a^
3	5-Ethyl-2-methyl-octane	062016-18-6	n.d.	0.29 ± 0.07^b^	0.40 ± 0.12^b^	0.63 ± 0.03^a^	0.23 ± 0.07^b^	0.24 ± 0.01^b^	0.64 ± 0.15^a^
4	4-Methyl-undecane	002980-69-0	n.d.	0.42 ± 0.09^bcd^	0.55 ± 0.15^bc^	0.78 ± 0.04^ab^	0.29 ± 0.08^cd^	0.27 ± 0.01^cd^	1.16 ± 0.49^a^
5	3,7-Dimethyl-decane	017312-54-8	n.d.	0.48 ± 0.27^a^	0.32 ± 0.26^ab^	n.d.	0.32 ± 0.26^ab^	n.d.	0.31 ± 0.04^ab^
6	3,8-Dimethyl-decane	017312-55-9	n.d.	n.d.	n.d.	n.d.	n.d.	n.d.	n.d.
7	2-Methyl-undecane	007045-71-8	n.d.	0.15 ± 0.03^cd^	0.26 ± 0.11^bcd^	0.51 ± 0.06^b^	0.41 ± 0.34^bc^	0.90 ± 0.03^a^	0.50 ± 0.10^bc^
8	2,4-Dimethyl-undecane	017312-80-0	n.d.	0.55 ± 0.29^ab^	1.87 ± 1.81^a^	n.d.	n.d.	n.d.	n.d.
9	2,6-Dimethyl-undecane	017301-23-4	n.d.	0.34 ± 0.07^b^	0.61 ± 0.20^b^	n.d.	6.11 ± 4.08^a^	n.d.	1.31 ± 0.14^b^
10	2,3,7-Trimethyldecane	062238-13-5	0.15 ± 0.03^a^	n.d.	n.d.	n.d.	n.d.	n.d.	
11	4-Methyl-dodecane	006117-97-1	n.d.	0.17 ± 0.03^c^	0.31 ± 0.10^c^	0.88 ± 0.05^a^	0.21 ± 0.04^c^	0.22 ± 0.01^c^	0.63 ± 0.12^b^
12	2,4,6-Trimethyl-decane	062108-27-4	n.d.	0.36 ± 0.30^a^	0.22 ± 0.04^a^	0.45 ± 0.11^a^	0.40 ± 0.36^a^	0.17 ± 0.003^a^	0.38 ± 0.07^a^
13	4-Ethyl-undecane	017312-59-3	n.d.	0.40 ± 0.05^c^	0.64 ± 0.20^c^	1.65 ± 0.16^a^	0.46 ± 0.08^c^	0.45 ± 0.01^c^	1.23 ± 0.22^b^
14	Dodecane	000112-40-3	0.15 ± 0.03^c^	0.56 ± 0.10^bc^	0.96 ± 0.37^b^	1.70 ± 0.17^a^	0.56 ± 0.18^bc^	0.65 ± 0.02^b^	1.65 ± 0.27^b^
15	4,6-Dimethyl-dodecan	061141-72-8	n.d.	0.39 ± 0.36^bc^	0.22 ± 0.07^bc^	1.28 ± 0.48^a^	0.16 ± 0.04^c^	0.12 ± 0.01^c^	0.75 ± 0.15^ab^


No.	Compounds	CAS	Concentration (*μ*g/g)						
			Y1	P1	P2	P3	L1	L2	L3
16	4-Methyl-tridecane	026730-12-1	n.d.	n.d.	0.45 ± 0.13^a^	n.d.	n.d.	n.d.	0.46 ± 0.14^a^
17	2-Methyl-tridecane	001560-96-9	n.d.	0.40 ± 0.11^ab^	0.77 ± 0.24^a^	0.26 ± 0.06^b^	0.44 ± 0.07^ab^	0.44 ± 0.02^ab^	0.44 ± 0.40^ab^
18	2,6,10-Trimethyl-pentadecane	003892-00-0	n.d.	n.d.	n.d.	n.d.	n.d.	n.d.	n.d.
19	2,6,10,14-Tetramethyl-hexadecane	000638-36-8	n.d.	n.d.	n.d.	n.d.	n.d.	n.d.	n.d.
20	Tetradecane	000629-59-4	0.13 ± 0.02^b^	0.52 ± 0.10^a^	0.19 ± 0.01^ab^	0.30 ± 0.13^ab^	0.36 ± 0.29^ab^	0.25 ± 0.02^ab^	0.22 ± 0.03^ab^
21	Hexadecane	000544-76-3	0.07 ± 0.01^a^	0.55 ± 0.30^a^	0.33 ± 0.22^a^	0.47 ± 0.09^a^	0.37 ± 0.34^a^	0.14 ± 0.01^a^	0.24 ± 0.05^a^
22	Heptadecane	000629-78-7	0.84 ± 0.07^a^	n.d.	n.d.	n.d.	n.d.	n.d.	n.d.
23	Octadecane	000593-45-3	0.11 ± 0.02^a^	n.d.	n.d.	n.d.	n.d.	n.d.	n.d.
24	Eicosane	000112-95-8	n.d.	0.38 ± 0.34^ab^	0.20 ± 0.06^b^	0.71 ± 0.05^a^	0.41 ± 0.36^ab^	0.34 ± 0.13^ab^	0.19 ± 0.07^b^

The results were expressed as mean ± standard deviation (*n* = 3). Mean values with different lowercase letters in the same row are significantly different at *p* < 0.05. ^1^n.d., not detected.

**Table 3:** HS-SPME-GC-MS integration parameters of VOCs in lipase-catalyzed alcoholized butter.

No.	Compounds	CAS	Concentration (*μ*g/g)					
			EP1	EP2	EP3	EL1	EL2	EL3
	**ACIDS**		**n.d.**	**n.d.**	**n.d.**	**n.d.**	**n.d.**	**n.d.**
1	Butanoic acid	000107-92-6	n.d.	n.d.	n.d.	n.d.	n.d.	n.d.
2	Hexanoic acid	000142-62-1	n.d.	n.d.	n.d.	n.d.	n.d.	n.d.
3	Octanoic acid	000124-07-2	n.d.	n.d.	n.d.	n.d.	n.d.	n.d.
4	n-Decanoic acid	000334-48-5	n.d.	n.d.	n.d.	n.d.	n.d.	n.d.
5	Nonanoic acid	000112-05-0	n.d.	n.d.	n.d.	n.d.	n.d.	n.d.
6	Dodecanoic acid	000143-07-7	n.d.	n.d.	n.d.	n.d.	n.d.	n.d.
	**ALDEHYDES**		**n.d.**	**n.d.**	**n.d.**	**n.d.**	**n.d.**	**n.d.**
1	Phenylacetaldehyde	000122-78-1	n.d.	n.d.	n.d.	n.d.	n.d.	n.d.
2	Nonanal	000124-19-6	n.d.	n.d.	n.d.	n.d.	n.d.	n.d.
	**ALCOHOLS**		**0.27** **±** **0.05**^**ab**^	**0.25** **±** **0.13**^**ab**^	**0.36** **±** **0.09**^**ab**^	**0.19** **±** **0.05**^**b**^	**0.77** **±** **0.38**^**a**^	**0.62** **±** **0.30**^**ab**^
1	Ethanol	000064-17-5	0.07 ± 0.05^a^	0.18 ± 0.002^a^	0.06 ± 0.05^a^	0.06 ± 0.002^a^	0.05 ± 0.02^a^	0.30 ± 0.37^a^
2	3-Methyl-2-hexanol	002313-65-7	n.d.	n.d.	n.d.	n.d.	0.46 ± 0.06^a^	n.d.
3	5-Methyl-2-hexanol	000627-59-8	n.d.	n.d.	0.16 ± 0.002^a^	n.d.	0.25 ± 0.003^a^	n.d.
4	2-Octanol	000123-96-6	0.10 ± 0.04^a^	0.09 ± 0.03^a^	0.13 ± 0.03^a^	0.09 ± 0.03^a^	0.11 ± 0.04^a^	0.14 ± 0.03^a^
5	5-Methyl-2-heptanol	054630-50-1	0.09 ± 0.02^a^	0.10 ± 0.02^a^	0.12 ± 0.03^a^	0.12 ± 0.03^a^	0.15 ± 0.05^a^	0.18 ± 0.04^a^
6	6-Methyl-2-heptanol	004730-22-7	n.d.	n.d.	n.d.	n.d.	0.11 ± 0.03^a^	n.d.
	**KETONES**		**n.d.**	**n.d.**	**n.d.**	**n.d.**	**n.d.**	**0.24** **±** **0.01**^**a**^
1	2-Heptanone	000110-43-0	n.d.	n.d.	n.d.	n.d.	n.d.	n.d.
2	2-Nonanone	000821-55-6	n.d.	n.d.	n.d.	n.d.	n.d.	0.24 ± 0.01^a^
3	2-Undecanone	000112-12-9	n.d.	n.d.	n.d.	n.d.	n.d.	n.d.
4	2-Tridecanone	000593-08-8	n.d.	n.d.	n.d.	n.d.	n.d.	n.d.
5	2-Pentadecanone	002345-28-0	n.d.	n.d.	n.d.	n.d.	n.d.	n.d.
	**LACTONES**		**0.11** **±** **0.01**^**a**^	**0.07** **±** **0.0003**^**a**^	**0.05** **±** **0.03**^**a**^	**0.10** **±** **0.05**^**a**^	**0.09** **±** **0.04**^**a**^	**0.13** **±** **0.06**^**a**^
1	*δ*-Octalactone	000698-76-0	n.d.	n.d.	n.d.	n.d.	n.d.	n.d.
2	*δ*-Decalactone	000705-86-2	n.d.	n.d.	n.d.	n.d.	n.d.	n.d.
3	*γ*-6-(Z)-Dodecenolactone	018679-18-0	n.d.	n.d.	n.d.	n.d.	n.d.	n.d.
4	*γ*-Undecalactone	000104-67-6	n.d.	n.d.	n.d.	n.d.	n.d.	n.d.
5	*δ*-Dodecalactone	000713-95-1	n.d.	n.d.	n.d.	n.d.	n.d.	n.d.
6	*δ*-Tetradecalactone	002721-22-4	0.11 ± 0.01^a^	0.07 ± 0.0003^a^	0.05 ± 0.03^a^	0.10 ± 0.05^a^	0.09 ± 0.04^a^	0.13 ± 0.06^a^
	**ESTERS**		**3.25** **±** **0.55**^**b**^	**5.57** **±** **1.53**^**a**^	**4.89** **±** **0.17**^**ab**^	**3.20** **±** **0.80**^**b**^	**4.18** **±** **0.40**^**ab**^	**4.56** **±** **0.85**^**ab**^
1	Hexanoic acid, methyl ester	000106-70-7	n.d.	n.d.	n.d.	n.d.	n.d.	n.d.
2	Octanoic acid, methyl ester	000111-11-5	n.d.	n.d.	n.d.	n.d.	n.d.	n.d.
3	Methoxyacetic acid, pentyl ester	168920-35-2	n.d.	0.37 ± 0.001^b^	0.56 ± 0.04^ab^	0.58 ± 0.23^a^	0.57 ± 0.19^a^	n.d.
4	Methoxyacetic acid, decyl ester	259141-02-1	n.d.	0.43 ± 0.17^a^	0.62 ± 0.15^a^	0.50 ± 0.19^a^	0.55 ± 0.11^a^	0.61 ± 0.07^a^


No.	Compounds	CAS	Concentration (*μ*g/g)					
			EP1	EP2	EP3	EL1	EL2	EL3
5	Methoxyacetic acid, 2-pentyl ester	1000282-69-2	0.11 ± 0.02^bc^	n.d.	0.37 ± 0.09^a^	0.30 ± 0.14^ab^	0.31 ± 0.13^ab^	n.d.
6	Oxalic acid, 6-ethyloct-3-yl hexyl ester	1000309-34-4	n.d.	n.d.	n.d.	n.d.	n.d.	n.d.
7	Octanoic acid, ethyl ester	000106-32-1	0.57 ± 0.12^ab^	0.80 ± 0.21^a^	0.66 ± 0.10^a^	0.32 ± 0.07^b^	0.49 ± 0.06^ab^	0.75 ± 0.18^a^
8	Decanoic acid, methyl ester	000110-42-9	n.d.	n.d.	n.d.	n.d.	n.d.	n.d.
9	Decanoic acid, ethyl ester	000110-38-3	1.31 ± 0.24^bc^	2.35 ± 0.74^a^	1.45 ± 0.06^bc^	0.71 ± 0.09^c^	1.14 ± 0.08^bc^	1.81 ± 0.32^ab^
10	Dodecanoic acid, methyl ester	000111-82-0	n.d.	n.d.	n.d.	n.d.	n.d.	n.d.
11	Dodecanoic acid, ethyl ester	000106-33-2	0.53 ± 0.05^bcd^	1.01 ± 0.25^a^	0.67 ± 0.10^abc^	0.26 ± 0.02^d^	0.44 ± 0.11^cd^	0.85 ± 0.24^ab^
12	Methyl tetradecanoate	000124-10-7	n.d.	n.d.	n.d.	n.d.	n.d.	n.d.
13	Methoxyacetic acid, 2-pentadecyl ester	1000282-05-1	0.32 ± 0.07^a^	0.35 ± 0.09^a^	0.33 ± 0.02^a^	0.27 ± 0.04^a^	0.30 ± 0.04^a^	n.d.
14	Methoxyacetic acid, octyl ester	029267-10-5	0.20 ± 0.03^ab^	0.23 ± 0.07^ab^	0.21 ± 0.04^ab^	0.13 ± 0.02^b^	0.20 ± 0.02^ab^	0.28 ± 0.08^a^
15	Methoxyacetic acid, 3-tetradecyl ester	1000282--04-9	n.d.	n.d.	n.d.	0.08 ± 0.02^a^	n.d.	n.d.
16	Methoxyacetic acid, 3-tridecyl ester	1000282-04-6	0.11 ± 0.02^a^	0.12 ± 0.03^a^	0.10 ± 0.02^a^	0.08 ± 0.001^b^	0.08 ± 0.005^ab^	0.11 ± 0.02^a^
17	Acetic acid, 3,7,11,15-tetramethyl-hexadecyl ester	1000193-63-0	n.d.	n.d.	n.d.	n.d.	n.d.	n.d.
18	Hexadecanoic acid, methyl ester	000112-39-0	n.d.	n.d.	n.d.	n.d.	n.d.	n.d.
19	Octadecanoic acid, ethyl ester	000111-61-5	0.10 ± 0.02^b^	0.17 ± 0.04^a^	0.11 ± 0.01^ab^	0.07 ± 0.0001^c^	0.09 ± 0.02^b^	0.15 ± 0.01^ab^
	**PHENOLS**		**n.d.**	**n.d.**	**n.d.**	**0.28** **±** **0.09**^**a**^	**n.d.**	**0.21** **±** **0.05**^**a**^
1	2,4-Bis(1,1-dimethylethyl)- phenol	000096-76-4	n.d.	n.d.	n.d.	0.28 ± 0.09^a^	n.d.	0.21 ± 0.05^a^
	**OLEFINS**		**0.59** **±** **0.12**^**a**^	**0.66** **±** **0.22**^**a**^	**0.13** **±** **0.02**^**b**^	**0.13** **±** **0.02**^**b**^	**0.13** **±** **0.02**^**b**^	**0.19** **±** **0.03**^**b**^
1	8-Heptadecene	002579-04-6	n.d.	n.d.	n.d.	n.d.	n.d.	n.d.
2	2,6,10,14-Tetramethyl-2-hexadecene	056554-34-8	0.43 ± 0.07^a^	0.58 ± 0.19^a^	0.09 ± 0.02^b^	0.09 ± 0.01^b^	0.09 ± 0.02^b^	0.13 ± 0.02^b^
3	2-Hexadecene, 3,7,11,15-tetramethyl-, [R-[R*,R*-(E)]]-	014237-73-1	0.23 ± 0.13^a^	0.08 ± 0.03^a^	0.04 ± 0.004^a^	0.04 ± 0.01^a^	0.04 ± 0.004^a^	0.06 ± 0.02^a^


No.	Compounds	CAS	Concentration (*μ*g/g)					
			EP1	EP2	EP3	EL1	EL2	EL3
	**ALKANES**		**2.04** **±** **0.22**^**ab**^	**2.64** **±** **0.96**^**ab**^	**1.74** **±** **0.21**^**ab**^	**1.45** **±** **0.20**^**b**^	**2.20** **±** **0.09**^**ab**^	**2.76** **±** **0.55**^**a**^
1	2,4-Dimethyl-heptane	002213-23-2	0.72 ± 0.07^ab^	0.84 ± 0.29^a^	0.60 ± 0.10^ab^	0.41 ± 0.05^b^	0.66 ± 0.08^ab^	0.80 ± 0.13^a^
2	4-Ethyl-decane	001636-44-8	n.d.	n.d.	n.d.	n.d.	n.d.	n.d.
3	5-Ethyl-2-methyl-octane	062016-18-6	n.d.	n.d.	n.d.	n.d.	n.d.	n.d.
4	4-Methyl-undecane	002980-69-0	n.d.	n.d.	n.d.	n.d.	n.d.	n.d.
5	3,7-Dimethyl-decane	017312-54-8	0.31 ± 0.06^ab^	0.38 ± 0.10^ab^	0.33 ± 0.07^ab^	0.26 ± 0.03^b^	0.35 ± 0.03^ab^	0.49 ± 0.11^a^
6	3,8-Dimethyl-decane	017312-55-9	0.39 ± 0.07^ab^	0.52 ± 0.15^a^	n.d.	0.29 ± 0.04^b^	0.38 ± 0.02^ab^	n.d.
7	2-Methyl-undecane	007045-71-8	n.d.	n.d.	n.d.	n.d.	n.d.	0.26 ± 0.06^a^
8	2,4-Dimethyl-undecane	017312-80-0	n.d.	n.d.	n.d.	n.d.	n.d.	n.d.
9	2,6-Dimethyl-undecane	017301-23-4	n.d.	n.d.	n.d.	n.d.	n.d.	0.40 ± 0.09^a^
10	2,3,7-Trimethyldecane	062238-13-5	n.d.	n.d.	n.d.	n.d.	n.d.	n.d.
11	4-Methyl-dodecane	006117-97-1	0.18 ± 0.04^b^	0.21 ± 0.05^b^	0.24 ± 0.03^b^	0.19 ± 0.03^b^	0.24 ± 0.04^b^	0.38 ± 0.08^a^
12	2,4,6-Trimethyl-decane	062108-27-4	n.d.	n.d.	n.d.	n.d.	n.d.	n.d.
13	4-Ethyl-undecane	017312-59-3	n.d.	n.d.	n.d.	n.d.	n.d.	n.d.
14	Dodecane	000112-40-3	n.d.	n.d.	n.d.	n.d.	n.d.	n.d.
15	4,6-Dimethyl-dodecan	061141-72-8	n.d.	n.d.	n.d.	n.d.	n.d.	n.d.
16	4-Methyl-tridecane	026730-12-1	n.d.	n.d.	n.d.	n.d.	n.d.	n.d.
17	2-Methyl-tridecane	001560-96-9	n.d.	n.d.	n.d.	n.d.	n.d.	n.d.
18	2,6,10-Trimethyl-pentadecane	003892-00-0	n.d.	0.04 ± 0.0002^b^	0.03 ± 0.003^a^	n.d.	n.d.	n.d.
19	2,6,10,14-Tetramethyl-hexadecane	000638-36-8	n.d.	n.d.	n.d.	0.02 ± 0.0006^ab^	0.05 ± 0.01^a^	n.d.
20	Tetradecane	000629-59-4	n.d.	n.d.	n.d.	n.d.	n.d.	n.d.
21	Hexadecane	000544-76-3	0.44 ± 0.06^a^	0.64 ± 0.14^b^	0.37 ± 0.06^a^	0.30 ± 0.04^a^	0.34 ± 0.04^a^	0.43 ± 0.09^a^
22	Heptadecane	000629-78-7	n.d.	0.25 ± 0.08^a^	0.17 ± 0.01^a^	n.d.	0.19 ± 0.02^a^	n.d.
23	Octadecane	000593-45-3	n.d.	n.d.	n.d.	n.d.	n.d.	n.d.
24	Eicosane	000112-95-8	n.d.	n.d.	n.d.	n.d.	n.d.	n.d.

The results were expressed as mean ± standard deviation (*n* = 3). Mean values with different lowercase letters in the same row are significantly different at *p* < 0.05.

In the P and L series, lipase-catalyzed hydrolysis of triglycerides generated FFAs, predominantly butyric acid, hexanoic acid, octanoic acid, and decanoic acid. In the P series, acids content increased from 1.96 μg/g to 80.66 μg/g with prolonged reaction time, while in the L series, it peaked at 55.26 μg/g at 48 h of hydrolysis and then declined to 25.42 μg/g at 72 h. The pronounced accumulation of acids in the P series was mainly associated with the greater extent of hydrolysis, suggesting a relatively higher hydrolytic activity of Palatase 20,000 L under the present conditions, which is consistent with the findings of Cong et al. (2025). In contrast, the initial increase followed by a decrease in acid content in the L series may reflect a gradual decline in the catalytic activity of Lipase MHA 10SD during prolonged reaction, which limited further triglyceride hydrolysis and acid production (Jiang et al., 2024). The subsequent decrease in acid content may also be associated with the further oxidation of acids to form aldehydes and ketones, collectively contributing to the decline in acid content during the later stages of the reaction (Mu et al., 2024). In contrast to the P and L series, no acids were detected in the EP and EL series. Instead, new esters were generated, including octanoic acid ethyl ester, decanoic acid ethyl ester, dodecanoic acid ethyl ester, and octadecanoic acid ethyl ester. Ethanol addition markedly reshaped the ester profile of the lipase-catalyzed treated butter. The P and L series contained several methyl esters, such as octanoic acid methyl ester, decanoic acid methyl ester and dodecanoic acid methyl ester, whereas the EP and EL series were characterized by multiple ethyl esters and methoxyacetic acid esters. This shift occurred because ethanol acted as a competing alcohol substrate, prompting lipases to preferentially catalyze fatty acid ethyl esters formation (Fang et al., 2025). Among the fatty acid ethyl esters, decanoic acid ethyl ester was particularly abundant, reaching 2.35 μg/g in EP2 and 1.81 μg/g in EL3. Octanoic acid ethyl ester and dodecanoic acid ethyl ester were also consistently detected in the alcoholyzed samples. Compared with methyl esters, ethyl esters are milder and more persistent, imparting a more pleasant aroma to the butter. Ethyl octanoate exhibits pineapple, apple and brandy-like notes, ethyl decanoate presents pear and brandy nuances, and ethyl laurate offers mild fruity and floral notes (Bennis et al., 2024; Duan et al., 2024).

Aldehydes are primarily formed through the oxidation of fatty acids, particularly unsaturated fatty acids (Mu et al., 2024). Due to their extremely low odor thresholds, they contribute pleasant flavor notes to butter, such as nonanal imparts fatty, floral, and citrus aromas, while phenylacetaldehyde exhibits sweet, hyacinth-like aromas (The Good Scents Company Information System*, 2025*). Both of these aldehydes were detected in Y1 (phenylacetaldehyde 0.55 μg/g, nonanal 0.2 μg/g) but disappeared after lipase-catalyzed hydrolysis and lipase-catalyzed alcoholysis. The absence of these aldehydes after lipase-catalyzed treatment indicates that the aldehyde profile of butter was markedly altered by hydrolysis and alcoholysis. Ketones are mainly produced by *β*-oxidation of FFAs and the subsequent decarboxylation reaction, play a significant role in shaping the overall flavor profile due to their ultra-low odor thresholds (Yan et al., 2020). Lactones are formed through intramolecular esterification of hydroxy fatty acids, imparting creamy, coconut, and peach-like odors to butter, while their low sensory thresholds also help attenuate the pungent notes of FFAs (Saeki et al., 2022). In the P and L series, the total ketones content ranged from 2.07 μg/g to 8.56 μg/g, with 2-heptanone, 2-nonanone, and 2-undecanone as the main contributors. The P3 exhibited the highest total ketone content (8.56 μg/g), while the L series showed relatively lower levels, ranging from 3.31 to 4.66 μg/g. Lactones were also detected at relatively higher levels in the P and L series. Among lactones, *δ*-octalactone reached 1.42 μg/g in P3 and 0.87 μg/g in L2 and L3, while *γ*-undecalactone ranged from 0.30 to 0.60 μg/g in the P and L series. *δ*-Dodecalactone reached 0.66 in P3 and 0.49 in L2. These lactones are associated with characteristic coconut-like, peach-like, and creamy or fruity aroma characteristics and may contribute to the aroma of hydrolyzed butter (The Good Scents Company Information System*, 2025*). The P3 exhibited the highest levels of ketones, lactones and acids, which may be associated with the release of FFAs during lipase-catalyzed hydrolysis and their potential role as precursors for the formation of these compounds (Mu et al., 2024; Saeki et al., 2022; Yan et al., 2020). In contrast, ketones were largely absent from the EP and EL series, with only 2-nonanone detected at 0.24 μg/g in EL3. For the lactones, only δ-tetradecalactone was detected in the EP and EL series, at low concentrations of 0.05–0.13 μg/g. This may be related to the conversion of some FFAs into fatty acid ethyl esters, potentially reducing the availability of precursors for ketone and lactone formation (Fang et al., 2025). Ethanol may also affect lipase activity, which could further influence FFA release during enzymatic treatment (Rigos et al., 2006). The substantially lower abundance and diversity of ketones and lactones in the EP and EL series compared with the P and L series. This difference was accompanied by the absence of acids and the predominance of ethyl esters in the alcoholyzed samples, suggesting that ethanol addition redirected the VOCs toward esters rather than the acids, ketones, and lactones in the hydrolyzed samples. Additionally, several methyl-branched alcohols, including 3-methyl-2-hexanol and 5-methyl-2-hexanol, were detected in the lipase-catalyzed alcoholyzed butter. These compounds, commonly found in fermented foods and characterized by low odor thresholds, enriched the aroma profile of butter (Yildiz et al., 2026).

Fig. 2A and Fig. 3A demonstrate that ethanol significantly altered the flavor compounds composition of butter. The flavor compounds in lipase-catalyzed hydrolyzed butter were dominated by acids, ketones, and lactones, while ethanol induced a marked shift in the flavor profile, with esters, alkanes, and ketones becoming the predominant components. The reliability of the PLS-DA models were evaluated using a 200-permutation test (Fig. 2D and Fig. 3D). The PLS-DA models exhibited high goodness of fit (R^2^Y = 0.988 and R^2^Y = 0.974), predictive ability (Q^2^ = 0.896 and Q^2^ = 0.865), and the Q^2^ regression line had a negative intercept, suggesting that the models were not overfitted and exhibited good reliability. Based on the validated PLS-DA models, VOCs with VIP > 1 were selected as key discriminatory compounds. As shown in Fig. 2B and Fig. 3B, 30 key VOCs were identified in the lipase-catalyzed hydrolyzed butter, including 2-undecanone, 2-methylundecane, octanoic acid methyl ester, 2,4-bis(1,1-dimethylethyl)-phenol, dodecanoic acid methyl ester, 2-methyl-tridecane, undecalactone, 2,6,10,14-tetramethyl-2-hexadecene, decalactone, heptadecane, 8-heptadecene, octadecane, etc. These compounds were primarily alkanes, esters, and ketones. In the lipase-catalyzed alcoholyzed butter, 13 key VOCs were screened, including octanoic acid ethyl ester, decanoic acid ethyl ester, 2,6,10-trimethyl-pentadecane, dodecanoic acid ethyl ester, methoxyacetic acid 3-tetradecyl ester, octadecanoic acid ethyl ester, methoxyacetic acid 3-tridecyl ester, 2,4-bis(1,1-dimethylethyl)-phenol, 2,6,10,14-tetramethyl-heptadecane, hexadecane, tetradecalactone, 4-methyl-dodecane, 3,8-dimethyl-decane, and methoxyacetic acid pentyl ester, with esters being the major component. The clustering heatmap (Fig. 2C) reveals that the P and L series clustered closely, while the Y1 formed an independent cluster, indicating that lipase-catalyzed hydrolysis significantly altered the flavor profile of butter compared to the raw material. Similarly, Fig. 3C showed distinct differences in both the types and concentrations of VOCs between the EP and EL series. We also performed PCA analysis. For the P, L series and Y1 (Fig. 2E), PCA showed that PC1 and PC2 explained 63.8% and 13.5% of the total variance. Y1 was clearly separated from all lipase-catalyzed treatment samples. For the EP and EL series (Fig. 3E), PC1 and PC2 explained 36.5% and 16.5% of the total variance. The separation between EP and EL samples indicates that the two lipase treatments produced different volatile profiles, which were also influenced by reaction time. In summary, HS-SPME-GC–MS analysis indicated that lipase-catalyzed hydrolysis markedly enhanced the diversity and concentration of flavor compounds in butter, particularly acids, ketones, and lactones. In contrast, ethanol addition reshaped the flavor profile into one dominated by ethyl esters through directed esterification, highlighting the regulatory role of ethanol in butter flavor formation.

**Fig. 2:**
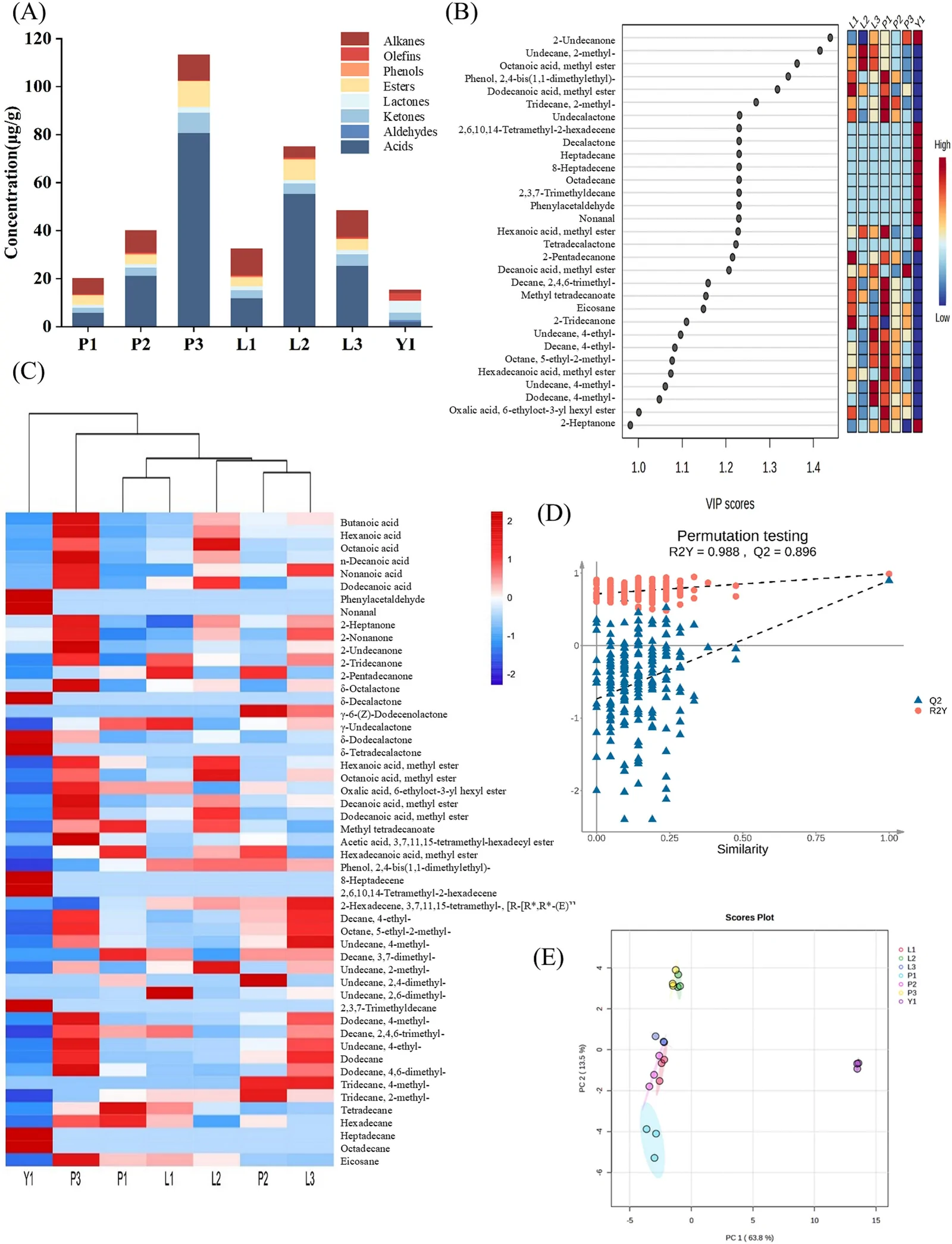
HS-SPME-GC-MS analysis of VOCs in raw and lipase-catalyzed hydrolyzed butter. (A) Concentration of classified VOCs; (B) VIP scores of VOCs; (C) Heatmap clustering of VOCs; (D) Plot of 200-permutation tests; (E) Score plot of PCA.

**Fig. 3:**
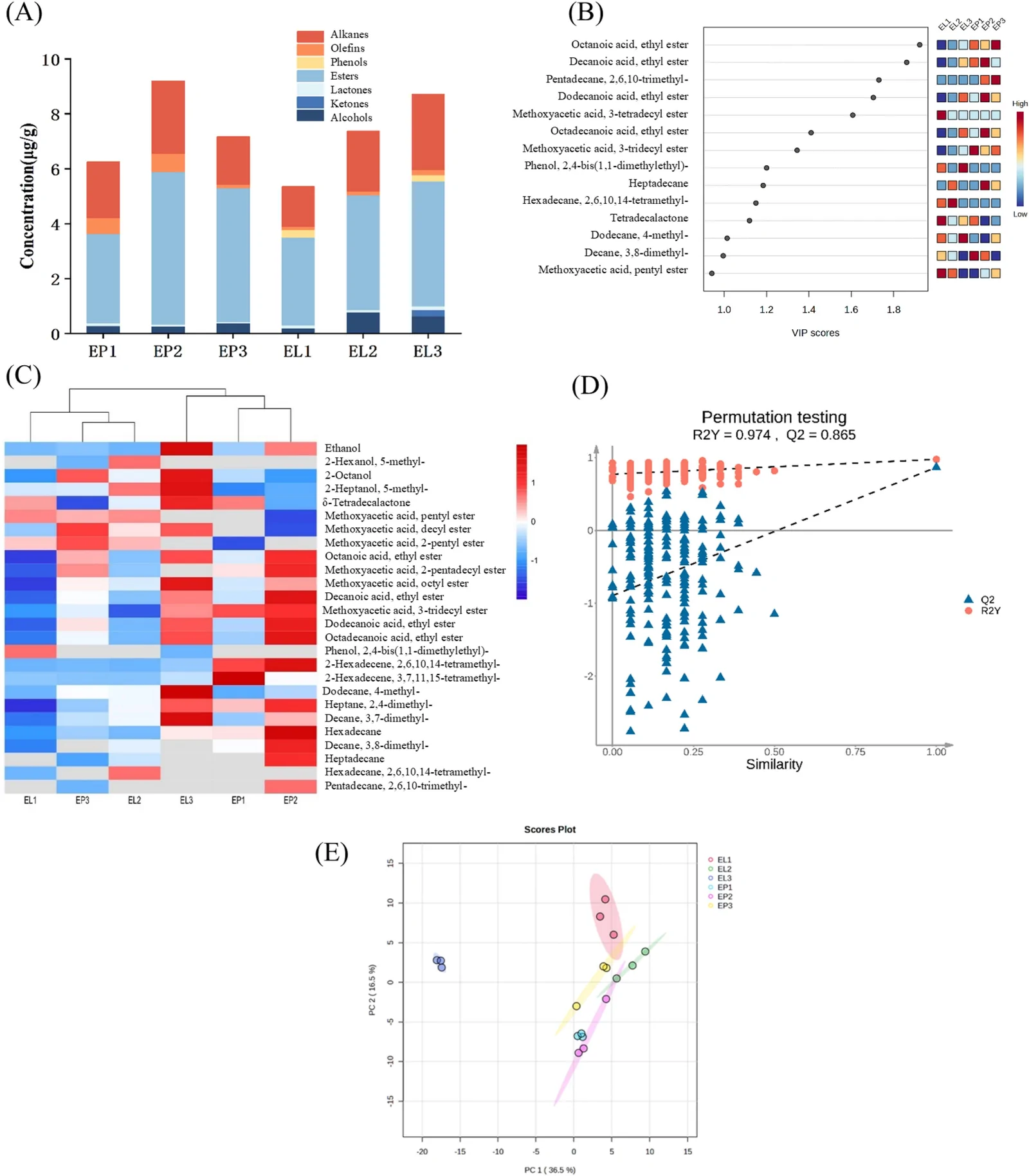
HS-SPME-GC-MS analysis of VOCs in lipase-catalyzed alcoholized butter. (A) Concentration of classified VOCs; (B) VIP scores of VOCs; (C) Heatmap clustering of VOCs; (D) Plot of 200-permutation tests; (E) Score plot of PCA.

#### 3.3.2 HS-GC–IMS analysis

VOCs in butter analyzed by HS-GC-IMS are listed in Table S2 and Table S3. A total of 43 VOCs were detected in Y1, including 2 acids, 9 aldehydes, 4 alcohols, 10 ketones, 13 esters, 2 olefins, 1 alkane, and 2 heterocyclic compounds. The P and L series contained 48 VOCs (1 acid, 7 aldehydes, 7 alcohols, 14 ketones, 7 esters, 4 olefins, 3 alkanes, and 5 heterocyclic compounds), while the EP and EL series had 17 VOCs (3 acids, 6 aldehydes, 2 ketones, 3 esters, 1 olefin, and 2 heterocyclic compounds). Notably, three acid compounds were detected in the EP and EL samples by HS-GC-IMS but were not detected by HS-SPME-GC–MS, indicating that the VOCs obtained by HS-GC-IMS differed substantially from those obtained by HS-SPME-GC–MS. Such differences may be related to the carbon-chain distribution of the detected VOCs. Previous studies have reported that HS-GC-IMS is particularly effective for detecting relatively small volatile molecules, especially those within the C2–C9 range, whereas HS-SPME-GC–MS provides complementary coverage of VOCs with long carbon-chain lengths (Cong et al., 2025).

As shown in Fig. 4A and Fig. 4B, the content of flavor compounds showed different changes with prolonged reaction time. Both the P and L series contained 2-heptanone (D), 2-heptanone (M), pentanal, 2-pentanone (D), 1-propanol, and isoamyl butyrate, with relatively consistent content. However, ethyl acetate (D) and butanal contents increased with prolonged hydrolysis time in the P series, whereas in the L series, their contents were significantly lower than those in the P series. These differences may be related to the distinct catalytic characteristics of the two lipases. Both lipases hydrolyzed triglycerides to produce short-chain FFAs (e.g., acetic acid and butyric acid), which may have served as important precursors for the formation of several volatile compounds during prolonged hydrolysis (Cong et al., 2025; Mu et al., 2024). Notably, Palatase 20,000 L appeared to exhibit a greater capacity for hydrolysis and ester formation under the present conditions, as reflected by the higher content of ethyl acetate in the P series. It may have efficiently hydrolyzed triglycerides to generate acetic acids and butyric acids, then converted acetic acid to form ethyl acetate (Fang et al., 2025). Similarly, the higher butyric acid content in the P series was accompanied by higher butanal levels, suggesting that lipid degradation may have promoted aldehyde formation (Yan et al., 2024). In contrast, Lipase MHA 10SD showed a relatively lower extent of hydrolysis and ester formation, resulting in lower ethyl acetate and butanal contents than those observed in the P series (Cong et al., 2025). The content of 2-methyltetrahydrofuran-3-one increased with time in the L series, whereas it was significantly lower in the P series. Additionally, levels of acetoin, 3-penten-2-one, undecane, and 2-methylpropanol were markedly higher in the L series than those in the P series. These differences may be related to the stronger esterification activity of Palatase 20,000 L, which appeared to divert FFAs toward esters formation and thereby limit metabolite accumulation from alternative branched pathways, whereas the weaker esterification activity of Lipase MHA 10SD may have allowed more FFAs to be directed toward ketones, alkanes, and other products (Bai et al., 2022). Following ethanol addition, 2,3-butanedione and aldehydes (hexanal, heptanal, octanal, furfural, 3-methyl-2-butenal) were more abundant in the EP series, but propanoic acid and 2-methylbutyric acid contents were higher in the EL series. These differences may be related to variations in substrate availability between the two series (Song et al., 2022). The higher abundance of aldehydes and 2,3-butanedione in the EP series may be associated with the greater release of FFAs by Palatase 20,000 L, which could provide more precursors for the formation of these compounds. In addition, acetoin serves as a precursor of 2,3-butanedione, and its conversion may have contributed to the higher 2,3-butanedione level observed in the EP series (Zhou et al., 2022).

**Fig. 4:**
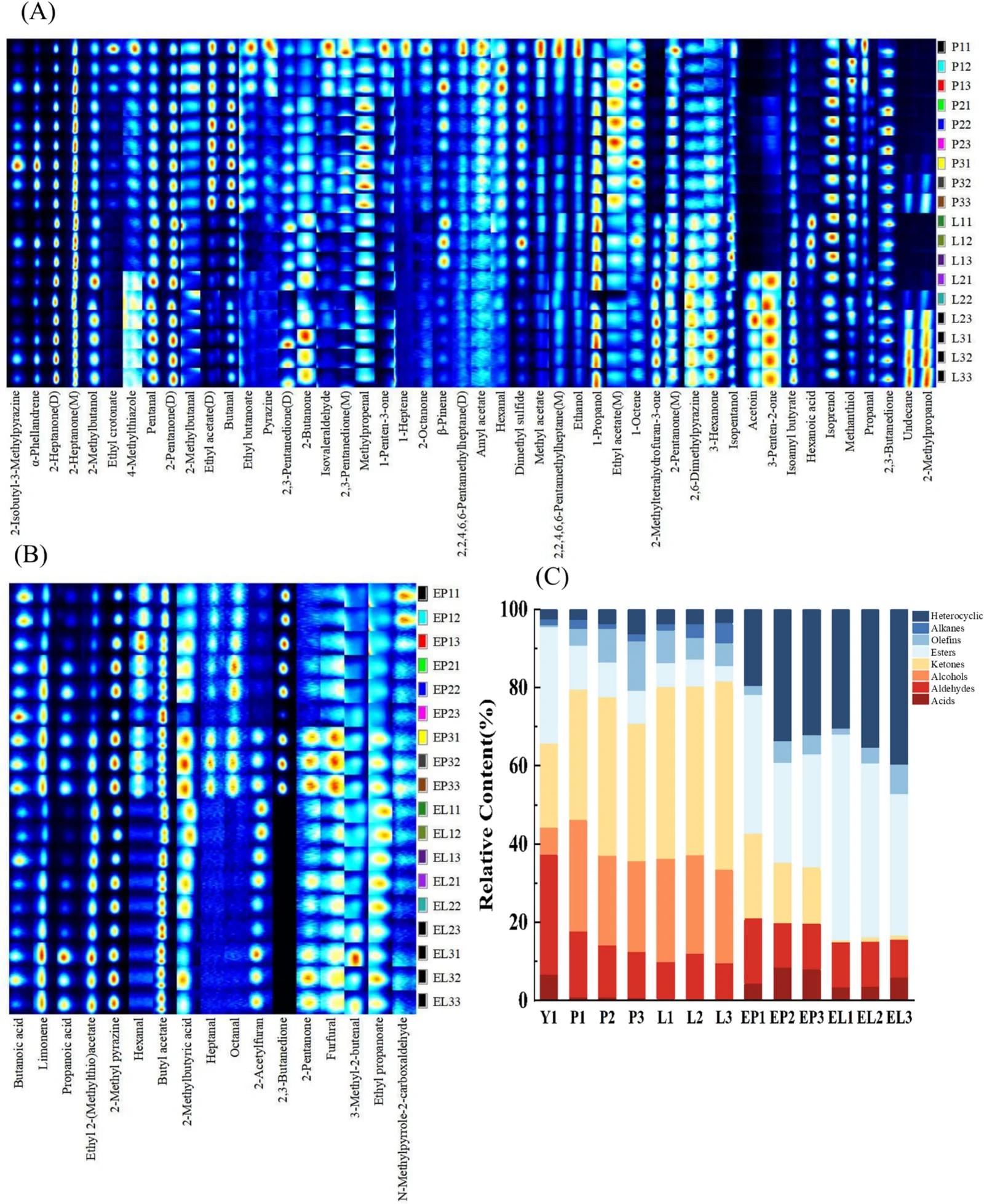
HS-GC-IMS analysis of VOCs in raw, lipase-catalyzed hydrolyzed and lipase-catalyzed alcoholized butter. (A) Fingerprints of lipase-catalyzed hydrolyzed butter; (B) Fingerprints of lipase-catalyzed alcoholized butter; (C) Relative content of VOCs as a percentage.

From an overall compositional perspective, ethanol induced a marked transformation in the flavor profile of lipase-catalyzed alcoholyzed butter. In the P and L series, the levels of flavor compounds ranked in descending order as ketones > alcohols > aldehydes > esters > olefins > heterocycles > alkanes > acids. In contrast, ethanol addition eliminated alkanes in the EP and EL series, with the order shifting to esters > ketones > heterocycles > aldehydes > acids > olefins. As shown in Fig. 4C, esters were the dominant class of flavor compounds in the EP and EL series. Notably, ethyl butyrate, which was not detected by HS-SPME-GC–MS but was identified by HS-GC-IMS, highlights the complementarity of these two analytical techniques and demonstrates the importance of combining multiple methods for a more comprehensive characterization of volatile compounds in butter. Ethyl butyrate content was significantly higher than other esters in the EP and EL series, indicating that butyric acid might be the primary product among the short-chain FFAs generated by lipase-catalyzed hydrolysis, consistent with fatty acid composition analysis results. Ester composition also differed between groups, with the P and L series mainly containing ethyl acetate (D) and methyl acetate, while ethanol addition may have influenced substrate utilization, promoting the formation of ethyl esters (Ginsburg & Jiménez-Flores, 2024). Ketone composition changed dramatically, with the P and L series containing 14 ketones (mainly 2-heptanone, 2-pentanone, 2,3-butanedione), while ethanol addition reduced ketone diversity to only two compounds (2,3-butanedione, 2-pentanone) in the EP series and only trace amounts of 2-pentanone in the EL series. This reduction might be associated with the competition of ethanol with triglycerides for lipase active sites, which could affect triglyceride hydrolysis, reducing FFAs release, and consequently decreasing the availability of precursors for ketones synthesis (Yan et al., 2020). Aldehyde composition also varied, with propanal, valeraldehyde, and butyraldehyde dominating the P and L series, while 3-methyl-2-butenal, hexanal, and carboxaldehyde were predominant in the EP and EL series. Across all groups, aldehyde contents decreased with prolonged reaction time. This decrease may be related to aldehyde loss and transformation during the reaction, potentially including oxidation and volatilization (Zhao et al., 2025).

The reliability of the PLS-DA models were evaluated using a 200-permutation test (Fig. 5A and Fig. 5C). The PLS-DA models exhibited high goodness of fit (R^2^Y = 0.998 and R^2^Y = 0.995), predictive ability (Q^2^ = 0.997 and Q^2^ = 0.992), and the Q^2^ regression line had a negative intercept, suggesting that the models were not overfitted and exhibited good reliability. Based on the validated PLS-DA models, VOCs with VIP > 1 were selected as key discriminatory compounds. For the P, L and Y series, VIP analysis (Fig. 5B) identified 48 key flavor compounds, including aldehydes, esters, and ketones, which play a critical role in shaping the overall flavor characteristics of butter. For the EP and EL series, VIP analysis (Fig. 5D) indicated that ethyl butyrate was a key ester compound. We also performed PCA analysis. For the P, L series and Y1 (Fig. 5E), PCA showed that PC1 and PC2 explained 80.1% and 10.1% of the total variance. Y1 was clearly separated from all lipase-catalyzed treatment samples, indicating substantial changes in the volatile profiles after lipase-catalyzed treatment. The P and L series were separated along PC2, indicating differences in their volatile profiles between the two lipase treatments, which were also influenced by reaction time. For the EP and EL series (Fig. 5F), PC1 and PC2 explained 49.3% and 35.9% of the total variance. The clear separation between EP and EL samples indicates differences in their volatile profiles, which were also affected by reaction time. The HS-GC-IMS results complemented HS-SPME-GC–MS findings, confirming that the use of multiple analytical methods ensures a more comprehensive characterization of VOCs. These results collectively confirmed that lipase-catalyzed hydrolysis enhanced the diversity of VOCs, while the introduction of ethanol alerted the flavor profile toward an ester-rich composition.

**Fig. 5:**
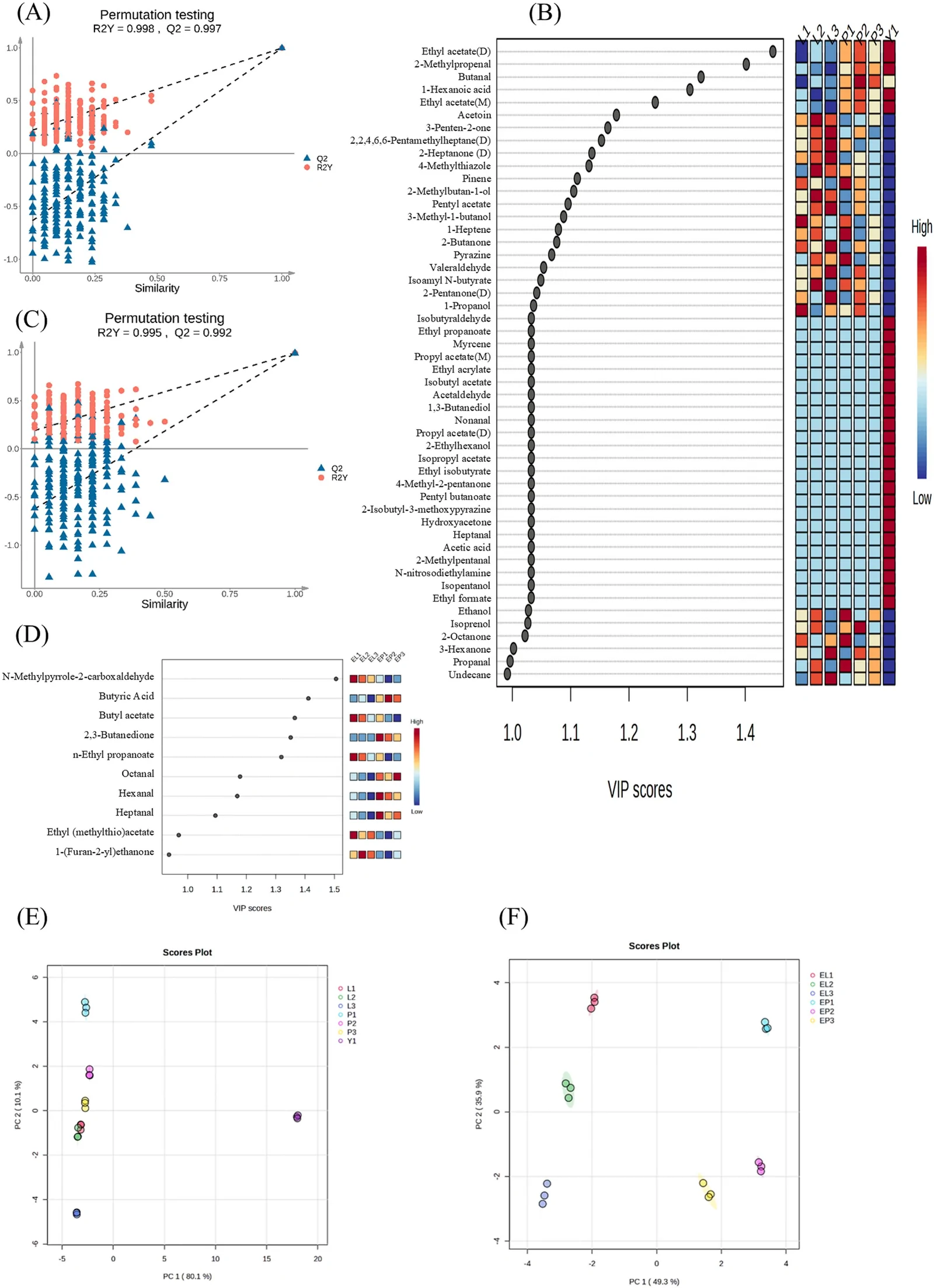
HS-GC-IMS analysis of VOCs in raw, lipase-catalyzed hydrolyzed and lipase-catalyzed alcoholized butter. (A) Plot of 200-permutation tests for raw and lipase-catalyzed hydrolyzed butter; (B) VIP scores of VOCs for raw and lipase-catalyzed hydrolyzed butter; (C) Plot of 200-permutation tests for lipase-catalyzed alcoholized butter; (D) VIP scores of VOCs for lipase-catalyzed alcoholized butter; (E) Score plot of PCA for lipase-catalyzed hydrolyzed butter; (F) Score plot of PCA for lipase-catalyzed alcoholized butter.

#### 3.3.3 ROAV analysis

Since relative content cannot fully reflect the contribution of VOCs to the overall flavor profile, this study calculated the ROAV based on odor thresholds to evaluate their flavor contribution and identify key flavor compounds. ROAV is positively correlated with the contribution of compounds to the overall flavor, meaning that a higher ROAV indicates a greater contribution. According to established criteria, compounds with ROAV ≥1 are major flavor contributors, those with 0.1 ≤ ROAV <1 are flavor modifiers, and those with ROAV <0.1 have negligible flavor contribution. (Xu et al., 2022).

As shown in Table S4, 23 VOCs in Y1 exhibited ROAV >0.1, with 7 exceeding 1. Notably, the P and L series contained 34 compounds with ROAV >0.1, 27 of which >1, indicating that lipase-catalyzed hydrolysis significantly enriched the flavor profile of butter. Specifically, isobutyraldehyde was the core flavor compound in Y1, while ethyl butyrate and 3-penten-2-one served as the key flavor compounds in the P and L series, respectively. Aldehydes, characterized by extremely low odor thresholds (0.0024–0.0151 mg/kg) and relatively high actual contents, play a critical role in shaping the overall flavor profile (Mu et al., 2024). Their flavor contributions exhibited temporal and enzyme specificity across different series. In the P series at 24 h, propanal (ROAV = 58.83) and isovaleraldehyde (ROAV = 52.43) imparted intense nutty and chocolate aromas (The Good Scents Company Information System*, 2025*). However, their ROAV gradually decreased with prolonged reaction time. In the L series, 2-methylbutanal (ROAV = 60.24) and valeraldehyde (ROAV = 49.52) maintained prominent roasted and fermented notes during prolonged reaction (The Good Scents Company Information System*, 2025*). Except for isobutyraldehyde and nonanal, most aldehydes in Y1 exhibited low ROAV, resulting in its relatively monotonous aroma. Similar to aldehydes, ketones also played a significant role in influencing the flavor profile of butte. In the L series, ketones reached higher ROAV at 72 h of reaction, potentially associated with intensified lipid oxidation and enhanced lipase-catalyzed reactions (Grebenteuch et al., 2021). Notably, 2,3-butanedione (ROAV = 76.92) and 2-heptanone (D) (ROAV = 18.05) imparted stronger creamy, fruity, and woody notes to the butter (The Good Scents Company Information System*, 2025*). The P series contributed herbaceous notes by 2-octanone (ROAV = 19.34) within 24 h (The Good Scents Company Information System*, 2025*). Although ketones in Y1 exhibited low ROAV, a large number of ketones generated by lipase-catalyzed hydrolysis compensated for the aromatic deficiencies of Y1. Ethyl butyrate (ROAV = 100), pentyl acetate (ROAV = 22.03), and isoamyl n-butyrate (ROAV = 11.55) were identified as core aroma esters, significantly contributing to the fruity flavor of the butter (Duan et al., 2024). Among these, ethyl butyrate showed the most prominent contribution in the P series at the initial reaction stage (ROAV = 100), while the L series efficiently generated ethyl butyrate (ROAV = 84.82) at the mid-stage of the reaction.

The ROAV of the lipase-catalyzed alcoholyzed butter are presented in Table S5. Compared with the P series, the ROAV of 2,3-butanedione increased sharply to 100 in the EP series, becoming the dominant flavor compound, while n-ethyl propanoate served as the dominant flavor compound in the EL series. Compared with the L series, the EL series exhibited elevated ROAV of esters coupled with suppressed aldehyde formation (e.g., a 60% reduction in the ROAV of hexanal), indicated that ethanol may have contributed to enhanced fruity notes of butter by esterification (Fang et al., 2025). Aldehydes in the EP series exhibited significantly higher ROAV than those in the EL series, particularly hexanal, heptanal, and octanal, which imparted more pronounced fatty aroma to the EP series (The Good Scents Company Information System*, 2025*). Compared with the P and L series, pyrazines also showed markedly increased ROAV in the EP and EL series. This increase may be attributed to the conversion of ethanol to acetaldehyde, which may have participated in Strecker degradation or combined with nitrogen-containing compounds (e.g., amino acids) to form pyrazine precursors that further condensed with amino compounds into pyrazine rings, imparting nutty and roasted aromas and enhancing the flavor complexity of the butter (Zhou et al., 2022).

## 4 Conclusion

This study utilized Anchor original flavor butter as the raw material, combining physicochemical indicators, flavoromics, and fatty acid composition to investigate differences between lipase-catalyzed hydrolyzed butter, lipase-catalyzed alcoholized butter and the original butter under different reaction times. Notably, Palatase 20,000 L exhibited stronger hydrolytic and esterification activities than Lipase MHA 10SD. Physicochemical analysis indicated that the ethanol reduced the AV of lipase-catalyzed hydrolyzed butter significantly. Palatase 20,000 L achieved a higher alcoholysis degree than lipase MHA 10SD, with an especially significant decrease (from 8.74 to 0.72 mg KOH·g^−1^). Lipase-catalyzed hydrolysis and lipase-catalyzed alcoholysis markedly altered the SFC-temperature profile, with all treated samples exhibited steeper melting curves than raw butter, showing a sharp decrease between 10 °C and 20 °C. GC indicated that lipase-catalyzed hydrolysis and lipase-catalyzed alcoholysis promoted the generation of SCFAs and MCFAs, with higher contents in the P and L series than in the EP and EL series. Flavoromics revealed that ethanol fundamentally reshaped the flavor profile of the butter. The addition of ethanol led to the originally dominant acids, ketones, and lactones almost completely disappeared or were significantly reduced, being replaced primarily by a range of ethyl esters. These esters not only imparted more distinct fruity and creamy aroma but also elevated the overall sensory experience from a simple milky taste to a richer aroma. These findings provide theoretical support for the directional regulation of butter flavor and offer practical value for developing specialty dairy fat ingredients. Subsequent research could focus on optimizing process parameters for precise control of lipid composition and flavor quality, and elucidating catalytic mechanisms to enable efficient and controllable lipase-catalyzed modification technologies.

## CRediT authorship contribution statement

**Xinyu Guo:** Writing – review & editing, Validation, Methodology, Data curation. **Yunjie Lei:** Investigation, Formal analysis. **Weinong Zhang:** Conceptualization. **Tao Yang:** Resources. **Zongyuan Wu:** Validation. **Jiangtao Feng:** Methodology, Conceptualization. **Yanxia Cong:** Supervision, Project administration, Funding acquisition.

## Declaration of competing interest

The authors declare that they have no known competing financial interests or personal relationships that could have appeared to influence the work reported in this paper.

## Data Availability

Data will be made available on request.

## Appendix A Supplementary data

Supplementary material: Table S1 Fatty acids composition of raw, lipase-catalyzed hydrolyzed and lipase-catalyzed alcoholized butter. Table S2 HS-GC-IMS integration parameters of VOCs in raw and lipase-catalyzed hydrolyzed butter. Table S3 HS-GC-IMS integration parameters of VOCs in lipase-catalyzed alcoholized butter. Table S4 ROAV of VOCs in raw and lipase-catalyzed hydrolyzed butter. Table S5 ROAV of VOCs in lipase-catalyzed alcoholized butter. Table S6 SFC curves of raw, lipase-catalyzed hydrolyzed and lipase-catalyzed alcoholized butter.
