# Supplementary material for: Lipase-catalyzed alcoholysis of butter for acid value reduction: a comparative study with lipase-catalyzed hydrolysis
Source: Food Chem X. 2026 Nov 5;40:104753. doi: 10.1016/j.fochx.2026.104753 (PMC13769537; doi:10.1016/j.fochx.2026.104753)
Supplement: Supplementary file 1 [file mmc1.docx]

**Table S1.** Fatty acids composition of raw, lipase-catalyzed hydrolyzed and lipase-catalyzed alcoholized butter.

| **FAs** | **Relative content (%)** | | | | | | | | | | | | |
| --- | --- | --- | --- | --- | --- | --- | --- | --- | --- | --- | --- | --- | --- |
|  | **Y1** | **P1** | **P2** | **P3** | **EP1** | **EP2** | **EP3** | **L1** | **L2** | **L3** | **EL1** | **EL2** | **EL3** |
| C4:0 | 0.66±0.04^e^ | 0.91±0.02^a^ | 0.88±0.01^ab^ | 0.86±0.001^c^ | 0.88±0.02^ab^ | 0.88±0.0001^ab^ | 0.75±0.004^cd^ | 0.76±0.02^c^ | 0.73±0.004^cd^ | 0.74±0.01^cd^ | 0.74±0.01^cd^ | 0.72±0.003^d^ | 0.74±0.001^cd^ |
| C6:0 | 0.01±0.0007^c^ | 0.02±0.0005^a^ | 0.02±0.0005^a^ | 0.02±0.0001^ab^ | 0.02±0.0004^a^ | 0.01±0.002^b^ | 0.01±0.0006^c^ | 0.01±0.0004^c^ | 0.01±0.0004^c^ | 0.01±0.0006^c^ | 0.01±0.0001^c^ | 0.01±0.0002^c^ | 0.01±0.0001^c^ |
| C8:0 | 0.75±0.05^b^ | 0.97±0.02^a^ | 0.94±0.02^a^ | 0.93±0.002^a^ | 0.94±0.02^a^ | 0.95±0.001^a^ | 0.74±0.003^b^ | 0.74±0.01^b^ | 0.72±0.01^b^ | 0.73±0.01^b^ | 0.72±0.0006^b^ | 0.71±0.01^b^ | 0.72±0.0003^b^ |
| C10:0 | 2.27±0.11^b^ | 3.11±0.04^a^ | 3.04±0.06^a^ | 3.03±0.01^a^ | 3.06±0.06^a^ | 3.13±0.01^a^ | 2.22±0.01^b^ | 2.21±0.01^b^ | 2.18±0.04^b^ | 2.21±0.001^b^ | 2.16±0.003^b^ | 2.15±0.02^b^ | 2.16±0.005^b^ |
| C11:0 | 0.22±0.01^a^ | 0.09±0.001^b^ | 0.09±0.001^b^ | 0.09±0.0004^b^ | 0.09±0.002^b^ | 0.09±0.0006^b^ | 0.04±0.0002^c^ | 0.04±0.0001^c^ | 0.04±0.0006^c^ | 0.04±0.0004^c^ | 0.04±0.0001^c^ | 0.04±0.0004^c^ | 0.04±0.0003^c^ |
| C12:0 | 4.49±0.11^c^ | 5.88±0.04^a^ | 5.84±0.11^a^ | 5.82±0.03^a^ | 5.93±0.06^a^ | 5.95±0.0009^a^ | 4.74±0.03^b^ | 4.73±0.02^b^ | 4.66±0.06^b^ | 4.73±0.01^b^ | 4.62±0.005^bc^ | 4.63±0.04^bc^ | 4.64±0.004^bc^ |
| C13:0 | 0.11±0.002^b^ | 0.13±0.002^a^ | 0.13±0.002^a^ | 0.13±0.0008^a^ | 0.13±0.002^a^ | 0.13±0.0005^a^ | 0.08±0.0005^c^ | 0.08±0.0005^c^ | 0.08±0.0008^c^ | 0.08±0.0001^c^ | 0.08±0.0001^c^ | 0.08±0.0005^c^ | 0.08±0.0004^c^ |
| C14:0 | 12.29±0.07^c^ | 13.48±0.004^a^ | 13.47±0.15^a^ | 13.49±0.08^a^ | 13.68±0.11^a^ | 13.64±0.003^a^ | 12.66±0.09^b^ | 12.67±0.06^b^ | 12.54±0.08^b^ | 12.64±0.02^b^ | 12.47±0.02^bc^ | 12.50±0.06^bc^ | 12.46±0.01^bc^ |
| C14:1 | 1.13±0.004^a^ | 0.82±0.02^b^ | 0.83±0.01^b^ | 0.82±0.01^b^ | 0.83±0.01^b^ | 0.83±0.0002^b^ | 1.14±0.01^a^ | 1.12±0.004^a^ | 1.12±0.01^a^ | 1.12±0.01^a^ | 1.12±0.003^a^ | 1.11±0.0008^a^ | 1.11±0.01^a^ |
| C15:0 | 1.35±0.001^b^ | 1.22±0.01^d^ | 1.22±0.01^d^ | 1.22±0.01^d^ | 1.25±0.001^c^ | 1.23±0.0004^cd^ | 1.37±0.01^ab^ | 1.37±0.01^a^ | 1.37±0.01^ab^ | 1.37±0.003^ab^ | 1.36±0.01^ab^ | 1.36±0.01^ab^ | 1.35±0.001^b^ |
| C15:1 | 0.05±0.02^a^ | 0.05±0.01^a^ | 0.04±0.02^a^ | 0.05±0.0006^a^ | 0.05±0.0009^a^ | 0.05±0.0002^a^ | 0.04±0.02^a^ | 0.04±0.02^a^ | 0.02±0.0003^a^ | 0.02±0.0002^a^ | 0.02±0.0008^a^ | 0.02±0.0007^a^ | 0.02±0.0007^a^ |
| C16:0 | 33.95±0.13^c^ | 32.04±0.19^e^ | 32.18±0.16^e^ | 32.27±0.24^de^ | 32.74±0.02^d^ | 32.38±0.004^de^ | 35.33±0.27^ab^ | 35.53±0.21^a^ | 35.26±0.08^ab^ | 35.34±0.07^ab^ | 35.16±0.05^ab^ | 35.27±0.11^ab^ | 34.96±0.06^b^ |
| C16:1 | 2.11±0.01^a^ | 1.45±0.0008^b^ | 1.49±0.03^b^ | 1.46±0.02^b^ | 1.47±0.04^b^ | 1.44±0.0001^b^ | 1.97±0.16^a^ | 1.95±0.19^a^ | 2.13±0.01^a^ | 1.95±0.17^a^ | 1.97±0.17^a^ | 2.13±0.0001^a^ | 2.10±0.01^a^ |
| C17:0 | 0.76±0.003^ab^ | 0.68±0.001^d^ | 0.71±0.03^bcd^ | 0.70±0.02^cd^ | 0.68±0.003^d^ | 0.67±0.0006^d^ | 0.77±0.02^a^ | 0.78±0.04^a^ | 0.77±0.01^a^ | 0.75±0.002^abc^ | 0.79±0.01^a^ | 0.80±0.01^a^ | 0.75±0.002^abc^ |
| C17:1 | 0.10±0.01^a^ | 0.01±0.0004^b^ | 0.01±0.0002^b^ | 0.01±0.001^b^ | 0.01±0.001^b^ | 0.01±0.0004^b^ | 0.01±0.0007^b^ | 0.01±0.0004^b^ | 0.01±0.0001^b^ | 0.01±0.0003^b^ | 0.01±0.0007^b^ | 0.01±0.0003^b^ | 0.01±0.0004^b^ |
| C18:0 | 11.13±0.08^abc^ | 11.33±0.15^ab^ | 11.43±0.004^a^ | 10.84±0.50^bc^ | 11.63±0.06^a^ | 11.48±0.0006^a^ | 10.68±0.10^c^ | 10.82±0.09^bc^ | 10.75±0.0007^c^ | 10.74±0.001^c^ | 10.73±0.02^c^ | 10.80±0.02^bc^ | 10.64±0.04^c^ |
| C18:1n9t | 6.62±0.14^a^ | 5.75±0.36^ab^ | 5.73±0.17^ab^ | 5.47±0.35^b^ | 5.28±0.31^de^ | 5.43±0.0008^de^ | 4.79±0.62^e^ | 4.37±0.41^e^ | 4.74±0.02^bcde^ | 4.44±0.13^cde^ | 5.39±0.25^bcd^ | 5.53±0.27^bc^ | 4.41±0.04^e^ |
| C18:1n9c | 18.28±0.56^abc^ | 17.72±0.08^de^ | 17.11±0.64^de^ | 17.80±0.17^de^ | 16.32±0.17^f^ | 16.21±0.0004^f^ | 17.94±0.17^de^ | 17.81±0.22^de^ | 18.16±0.14^de^ | 18.93±0.22^d^ | 18.09±0.26^abc^ | 18.08±0.0008^abc^ | 18.67±0.14^ab^ |
| C18:2n6t | 0.36±0.01^a^ | 0.39±0.01^a^ | 0.40±0.01^a^ | 0.39±0.01^a^ | 0.40±0.01^a^ | 0.39±0.0003^a^ | 0.30±0.002^a^ | 0.75±0.45^a^ | 0.30±0.003^a^ | 0.30±0.0003^a^ | 0.30±0.0009^a^ | 0.30±0.002^a^ | 0.30±0.005^a^ |
| C18:2n6c | 1.21±0.004^a^ | 1.21±0.06^a^ | 1.26±0.02^a^ | 1.24±0.02^a^ | 1.26±0.02^a^ | 1.23±0.0005^a^ | 1.21±0.01^a^ | 0.61±0.61^a^ | 1.21±0.01^a^ | 1.20±0.01^a^ | 1.21±0.002^a^ | 1.21±0.004^a^ | 1.19±0.01^a^ |

**Table S1.** (continued)

| **FAs** | **Relative content (%)** | | | | | | | | | | | | |
| --- | --- | --- | --- | --- | --- | --- | --- | --- | --- | --- | --- | --- | --- |
|  | **Y1** | **P1** | **P2** | **P3** | **EP1** | **EP2** | **EP3** | **L1** | **L2** | **L3** | **EL1** | **EL2** | **EL3** |
| C20:0 | 0.02±0.001^d^ | 0.16±0.002^c^ | 0.16±0.0001^c^ | 0.16±0.003^c^ | 0.17±0.0002^c^ | 0.16±0.0003^c^ | 0.22±0.002^ab^ | 0.22±0.003^ab^ | 0.22±0.0003^ab^ | 0.22±0.0005^ab^ | 0.22±0.0004^ab^ | 0.22±0.0008^a^ | 0.21±0.001^b^ |
| C18:3n6 | 0.02±0.01^b^ | 0.03±0.0006^ab^ | 0.11±0.08^ab^ | 0.03±0.003^ab^ | 0.07±0.04^ab^ | 0.12±0.0005^a^ | 0.03±0.0005^ab^ | 0.04±0.002^ab^ | 0.04±0.003^ab^ | 0.04±0.001^ab^ | 0.03±0.002^ab^ | 0.03±0.003^ab^ | 0.04±0.0001^ab^ |
| C20:1 | 0.07±0.04^c^ | 0.76±0.02^ab^ | 0.84±0.09^b^ | 0.76±0.0006^ab^ | 0.76±0.02^ab^ | 0.76±0.0005^ab^ | 0.69±0.01^b^ | 0.69±0.0006^b^ | 0.69±0.0005^b^ | 0.69±0.01^b^ | 0.69±0.005^b^ | 0.68±0.01^b^ | 0.68±0.01^b^ |
| C18:3n3 | 0.17±0.02^e^ | 0.60±0.07^abcd^ | 0.69±0.11^abcd^ | 0.54±0.06^bcd^ | 0.51±0.01^cd^ | 0.50±0.0001^d^ | 0.68±0.01^abcd^ | 0.73±0.05^ab^ | 0.70±0.05^abc^ | 0.64±0.05^abcd^ | 0.75±0.07^a^ | 0.68±0.09^abcd^ | 0.66±0.004^abcd^ |
| C21:0 | 1.02±0.04^a^ | 0.07±0.01^b^ | 0.14±0.07^b^ | 0.05±0.03^b^ | 0.05±0.02^b^ | 0.08±0.0001^b^ | 0.10±0.003^b^ | 0.07±0.02^b^ | 0.10±0.01^b^ | 0.11±0.003^b^ | 0.10±0.01^b^ | 0.07±0.04^b^ | 0.10±0.0008^b^ |
| C20:2 | 0.08±0.01^b^ | 0.14±0.02^b^ | 0.11±0.01^b^ | 0.23±0.10^ab^ | 0.35±0.10^a^ | 0.12±0.001^b^ | 0.12±0.01^b^ | 0.13±0.08^b^ | 0.11±0.0007^b^ | 0.12±0.02^b^ | 0.14±0.001^b^ | 0.11±0.01^b^ | 0.08±0.02^b^ |
| C22:0 | 0.10±0.02^c^ | 0.24±0.06^bc^ | 0.16±0.12^c^ | 0.17±0.11^bc^ | 0.32±0.03^abc^ | 0.54±0.001^a^ | 0.17±0.11^bc^ | 0.31±0.15^abc^ | 0.25±0.003^bc^ | 0.08±0.003^c^ | 0.24±0.10^bc^ | 0.24±0.04^bc^ | 0.45±0.06^ab^ |
| C20:3n6 | 0.13±0.03^a^ | 0.22±0.04^a^ | 0.21±0.07^a^ | 0.13±0.08^a^ | 0.29±0.07^a^ | 0.33±0.0009^a^ | 0.36±0.16^a^ | 0.16±0.08^a^ | 0.25±0.08^a^ | 0.24±0.0007^a^ | 0.23±0.01^a^ | 0.13±0.003^a^ | 0.32±0.03^a^ |
| C22:1n9 | 0.08±0.01^b^ | 0.12±0.05^ab^ | 0.19±0.08^ab^ | 0.21±0.10^ab^ | 0.15±0.003^ab^ | 0.31±0.0005^a^ | 0.21±0.06^ab^ | 0.13±0.09^ab^ | 0.15±0.08^ab^ | 0.12±0.01^ab^ | 0.11±0.02^ab^ | 0.07±0.005^b^ | 0.20±0.004^ab^ |
| C20:3n3 | 0.04±0.0003^ab^ | 0.09±0.04^ab^ | 0.12±0.05^ab^ | 0.37±0.23^a^ | 0.22±0.10^ab^ | 0.19±0.002^ab^ | 0.16±0.003^ab^ | 0.07±0.03^ab^ | 0.15±0.06^ab^ | 0.10±0.05^ab^ | 0.09±0.05^ab^ | 0.04±0.002^b^ | 0.19±0.01^ab^ |
| C20:4n6 | 0.05±0.02^d^ | 0.09±0.01^bcd^ | 0.21±0.12^abcd^ | 0.29±0.02^ab^ | 0.22±0.11^abcd^ | 0.38±0.01^a^ | 0.17±0.05^abcd^ | 0.09±0.02^bcd^ | 0.20±0.11^abcd^ | 0.09±0.03^bcd^ | 0.14±0.07^bcd^ | 0.06±0.0006^cd^ | 0.28±0.01^abc^ |
| C23:0 | 0.13±0.10^a^ | 0.02±0.001^a^ | 0.07±0.05^a^ | 0.19±0.08^a^ | 0.07±0.05^a^ | 0.14±0.0003^a^ | 0.12±0.01^a^ | 0.04±0.0002^a^ | 0.18±0.13^a^ | 0.04±0.0006^a^ | 0.13±0.08^a^ | 0.04±0.001^a^ | 0.26±0.01^a^ |
| C22:2 | 0.11±0.06^abc^ | 0.10±0.04^abc^ | 0.09±0.02^abc^ | 0.11±0.01^ab^ | 0.09±0.02^abc^ | 0.13±0.0003^a^ | 0.05±0.01^abc^ | 0.04±0.0006^c^ | 0.06±0.02^abc^ | 0.04±0.0007^c^ | 0.05±0.01^bc^ | 0.04±0.0008^c^ | 0.08±0.0004^abc^ |
| C24:0 | 0.06±0.06^a^ | 0.10±0.004^a^ | 0.09±0.002^a^ | 0.09±0.002^a^ | 0.09±0.002^a^ | 0.09±0.0001^a^ | 0.06±0.02^a^ | 0.09±0.002^a^ | 0.09±0.0002^a^ | 0.09±0.0003^a^ | 0.09±0.0005^a^ | 0.09±0.0001^a^ | 0.08±0.0004^a^ |
| C20:5 | 0.02±0.01^a^ | n.d.^1^ | n.d. | n.d. | n.d. | n.d. | 0.04±0.04^a^ | 0.47±0.46^a^ | 0.01±0.0006^a^ | 0.01±0.0004^a^ | 0.01±0.0005^a^ | 0.01±0.003^a^ | 0.01±0.0001^a^ |
| C24:1 | 0.05±0.05^a^ | n.d. | n.d. | n.d. | n.d. | n.d. | n.d. | n.d. | n.d. | n.d. | n.d. | 0.01±0.01^b^ | 0.01±0.01^b^ |
| C22:6n3 | 0.01±0.002^a^ | 0.01±0.0005^a^ | 0.02±0.0002^a^ | 0.01±0.0002^a^ | 0.01±0.0001^a^ | 0.02±0.002^a^ | 0.01±0.0002^a^ | 0.32±0.31^a^ | 0.01±0.0003^a^ | 0.01±0.0001^a^ | 0.01±0.0003^a^ | 0.01±0.0005^a^ | 0.01±0.0007^a^ |
| SCFAs | 0.67±0.03^e^ | 0.93±0.02^a^ | 0.90±0.02^ab^ | 0.88±0.001^b^ | 0.89±0.02^ab^ | 0.89±0.003^ab^ | 0.76±0.004^cd^ | 0.77±0.02^c^ | 0.74±0.005^cd^ | 0.74±0.01^cd^ | 0.75±0.01^cd^ | 0.73±0.003^d^ | 0.74±0.001^cd^ |
| MCFAs | 7.72±0.20^b^ | 10.05±0.10^a^ | 9.91±0.19^a^ | 9.87±0.04^a^ | 10.02±0.14^a^ | 10.12±0.01^a^ | 7.74±0.04^b^ | 7.72±0.04^b^ | 7.60±0.11^b^ | 7.72±0.002^b^ | 7.54±0.01^b^ | 7.54±0.07^b^ | 7.56±0.01^b^ |
| LCFAs | 91.62±0.22^a^ | 89.02±0.12^b^ | 89.19±0.21^b^ | 89.25±0.04^b^ | 89.08±0.16^b^ | 88.99±0.01^b^ | 91.50±0.03^a^ | 91.51±0.06^a^ | 91.66±0.12^a^ | 91.54±0.01^a^ | 91.71±0.003^a^ | 91.74±0.07^a^ | 91.70±0.01^a^ |

**Table S1.** (continued)

| **FAs** | **Relative content (%)** | | | | | | | | | | | | |
| --- | --- | --- | --- | --- | --- | --- | --- | --- | --- | --- | --- | --- | --- |
|  | **Y1** | **P1** | **P2** | **P3** | **EP1** | **EP2** | **EP3** | **L1** | **L2** | **L3** | **EL1** | **EL2** | **EL3** |
| SFAs | 69.30±0.27^d^ | 70.44±0.29^bc^ | 70.56±0.46^b^ | 70.07±0.11^bcd^ | 71.71±0.18^a^ | 71.55±0.01^a^ | 70.07±0.35^bcd^ | 70.48±0.34^bc^ | 69.94±0.16^bcd^ | 69.92±0.08^bcd^ | 69.64±0.10^cd^ | 69.74±0.36^bcd^ | 69.65±0.17^cd^ |
| MUFAs | 28.49±0.33^a^ | 26.67±0.35^cd^ | 26.23±0.43^d^ | 26.58±0.11^cd^ | 24.87±0.22^e^ | 25.05±0.001^e^ | 26.79±0.51^bcd^ | 26.12±0.26^d^ | 27.03±0.07^bcd^ | 27.29±0.07^bc^ | 27.39±0.18^bc^ | 27.64±0.26^ab^ | 27.21±0.21^bc^ |
| PUFAs | 2.21±0.07^b^ | 2.88±0.06^ab^ | 3.20±0.03^a^ | 3.36±0.0008^a^ | 3.42±0.40^a^ | 3.40±0.01^a^ | 3.14±0.16^a^ | 3.40±0.61^a^ | 3.03±0.23^a^ | 2.79±0.003^ab^ | 2.97±0.07^ab^ | 2.62±0.10^ab^ | 3.14±0.04^a^ |

The results were expressed as mean ± standard deviation (n = 3). Mean values with different lowercase letters in the same row are significantly different at *p* < 0.05. Y1: raw butter; P1-P3: hydrolysis by Palatase 20000 L for 24, 48, 72 h; EP1-EP3: alcoholysis by Palatase 20000 L for 24, 48, 72 h; L1-L3: hydrolysis by Lipase MHA 10SD for 24, 48, 72 h; EL1-EL3: alcoholysis by Lipase MHA 10SD for 24, 48, 72 h.^1^n.d., not detected.

**Table S2.** HS-GC-IMS integration parameters of VOCs in raw and lipase-catalyzed hydrolyzed butter.

| No | Compounds | RI^1^ | RT (s)^2^ | DT (ms)^3^ | Relative content (%) | | | | | | |
| --- | --- | --- | --- | --- | --- | --- | --- | --- | --- | --- | --- |
|  |  |  |  |  | **Y1** | **P1** | **P2** | **P3** | **L1** | **L2** | **L3** |
|  | **ACIDS** |  |  |  | **6.73±0.05^a^** | **0.81±0.03^c^** | **0.90±0.03^b^** | **0.70±0.01^d^** | **0.44±0.01^e^** | **0.14±0.001^f^** | **0.14±0.0005^f^** |
| 1 | Acetic acid | 587 | 37.2 | 1.0393 | 1.16±0.12^a^ | n.d. | n.d. | n.d. | n.d. | n.d. | n.d. |
| 2 | Butyric Acid | 813 | 233 | 1.1658 | n.d. | n.d. | n.d. | n.d. | n.d. | n.d. | n.d. |
| 3 | Propionic acid | 704 | 111.8 | 1.0975 | n.d. | n.d. | n.d. | n.d. | n.d. | n.d. | n.d. |
| 4 | 2-Methylbutyric acid | 899 | 352.9 | 1.2115 | n.d. | n.d. | n.d. | n.d. | n.d. | n.d. | n.d. |
| 5 | 1-Hexanoic acid | 1022 | 602.8 | 1.2947 | 5.57±0.12^a^ | 0.81±0.03^bc^ | 0.90±0.03^b^ | 0.70±0.01^c^ | 0.44±0.01^d^ | 0.14±0.001^e^ | 0.14±0.0005^e^ |
|  | **ALDEHYDES** |  |  |  | **30.70±0.21^a^** | **16.98±0.04^b^** | **13.23±0.13^c^** | **11.82±0.08^d^** | **9.47±0.15^e^** | **11.98±0.18^d^** | **9.45±0.05^e^** |
| 1 | Valeraldehyde | 724 | 128.9 | 1.4224 | n.d. | 2.34±0.04^d^ | 3.13±0.04^b^ | 2.60±0.01^c^ | 3.01±0.07^b^ | 3.12±0.21^b^ | 3.47±0.09^a^ |
| 2 | 2-Methylbutanal | 696 | 105.5 | 1.3966 | 0.59±0.03^b^ | 0.86±0.05^a^ | 0.63±0.03^b^ | 0.42±0.01^c^ | 0.40±0.01^c^ | 0.80±0.03^a^ | 0.36±0.01^c^ |
| 3 | Octanal | 1007 | 558.3 | 1.3983 | n.d. | n.d. | n.d. | n.d. | n.d. | n.d. | n.d. |
| 4 | Furfural | 804 | 223.2 | 1.3373 | n.d. | n.d. | n.d. | n.d. | n.d. | n.d. | n.d. |
| 5 | 3-Methyl-2-butenal | 784 | 200.1 | 1.0959 | n.d. | n.d. | n.d. | n.d. | n.d. | n.d. | n.d. |
| 6 | N-Methylpyrrole-2-carboxaldehyde | 1011 | 575.3 | 1.4937 | n.d. | n.d. | n.d. | n.d. | n.d. | n.d. | n.d. |
| 7 | Butanal | 614 | 50.1 | 1.2914 | 2.11±0.02^d^ | 2.26±0.03^c^ | 2.93±0.06^a^ | 2.43±0.01^b^ | 1.09±0.04^g^ | 1.53±0.01^e^ | 1.24±0.02^f^ |
| 8 | Isovaleraldehyde | 632 | 59.2 | 1.1961 | n.d. | 1.23±0.02^a^ | 0.36±0.005^d^ | 0.49±0.005^b^ | 0.29±0.01^e^ | 0.39±0.003^c^ | 0.14±0.003^f^ |
| 9 | 2-Methylpropenal | 575 | 34.8 | 1.2258 | 2.34±0.08^a^ | 1.06±0.04^d^ | 1.63±0.08^b^ | 1.25±0.03^c^ | 0.97±0.02^de^ | 0.86±0.04^ef^ | 0.83±0.04^f^ |
| 10 | Hexanal | 804 | 223 | 1.2534 | 0.96±0.05^a^ | 0.55±0.04^b^ | 0.43±0.02^c^ | 0.33±0.01^d^ | 0.32±0.02^d^ | 0.42±0.002^c^ | 0.13±0.002^e^ |
| 11 | Propanal | 756 | 163.4 | 1.04 | n.d. | 8.69±0.09^a^ | 4.13±0.09^c^ | 4.30±0.07^c^ | 3.38±0.07^d^ | 4.85±0.02^b^ | 3.28±0.08^d^ |
| 12 | Acetaldehyde | 749 | 160.9 | 0.9577 | 5.94±0.11^a^ | n.d. | n.d. | n.d. | n.d. | n.d. | n.d. |
| 13 | Nonanal | 1101 | 745.6 | 1.4805 | 3.47±0.08^a^ | n.d. | n.d. | n.d. | n.d. | n.d. | n.d. |
| 14 | Isobutyraldehyde | 552 | 19.5 | 1.1144 | 14.60±0.03^a^ | n.d. | n.d. | n.d. | n.d. | n.d. | n.d. |
| 15 | Heptanal | 900 | 355.5 | 1.3335 | 0.67±0.04^a^ | n.d. | n.d. | n.d. | n.d. | n.d. | n.d. |
| 16 | 2-Methylpentanal | 755 | 167.7 | 1.216 | 0.02±0.0008^a^ | n.d. | n.d. | n.d. | n.d. | n.d. | n.d. |
|  | **ALCOHOLS** |  |  |  | **6.88±0.23^f^** | **28.49±0.05^a^** | **23.04±0.48^e^** | **23.14±0.19^e^** | **26.37±0.29^b^** | **25.11±0.25^c^** | **24.02±0.26^d^** |
| 1 | 2-Methylbutan-1-ol | 732 | 137.1 | 1.4771 | n.d. | 0.39±0.07^d^ | 0.65±0.06^c^ | 0.47±0.003^d^ | 0.59±0.01^c^ | 1.08±0.03^a^ | 0.83±0.08^b^ |
| 2 | Ethanol | 964 | 467.4 | 1.0409 | n.d. | 7.40±0.70^a^ | 3.69±0.14^de^ | 4.64±0.12^c^ | 4.17±0.12^cd^ | 5.58±0.04^b^ | 3.33±0.14^e^ |
| 3 | 1-Propanol | 565 | 31.9 | 1.1129 | n.d. | 10.94±0.44^c^ | 12.66±0.18^a^ | 10.84±0.24^c^ | 12.83±0.02^a^ | 10.07±0.06^d^ | 11.91±0.15^b^ |
| 4 | 3-Methyl-1-butanol | 767 | 176.4 | 1.1190 | n.d. | 4.95±0.09^a^ | 2.48±0.12^d^ | 2.75±0.02^c^ | 4.99±0.13^a^ | 3.11±0.02^b^ | 2.70±0.05^c^ |
| 5 | Isoprenol | 734 | 138.7 | 1.1646 | n.d. | 1.11±0.09^b^ | 1.38±0.02^a^ | 0.93±0.05^c^ | 1.10±0.06^b^ | 1.17±0.02^b^ | 0.68±0.005^d^ |
| 6 | Methanethiol | 641 | 64.2 | 1.0398 | 1.10±0.12^d^ | 3.62±0.13^a^ | 2.05±0.02^c^ | 2.09±0.01^c^ | 2.53±0.05^b^ | 1.99±0.06^c^ | 1.19±0.02^d^ |

**Table S2.** (continued)

| No | Compounds | RI | RT (s) | DT (ms) | Relative content (%) | | | | | | |
| --- | --- | --- | --- | --- | --- | --- | --- | --- | --- | --- | --- |
|  |  |  |  |  | **Y1** | **P1** | **P2** | **P3** | **L1** | **L2** | **L3** |
| 7 | 2-Methylpropanol | 1132 | 970.5 | 1.1707 | n.d. | 0.09±0.01^d^ | 0.11±0.004^d^ | 1.40±0.03^c^ | 0.14±0.003^d^ | 2.10±0.15^b^ | 3.37±0.10^a^ |
| 8 | 1,3-Butanediol | 784 | 201 | 1.1240 | 4.37±0.09^a^ | n.d. | n.d. | n.d. | n.d. | n.d. | n.d. |
| 9 | 2-Ethylhexanol | 1039 | 622.8 | 1.4093 | 1.20±0.03^a^ | n.d. | n.d. | n.d. | n.d. | n.d. | n.d. |
| 10 | Isopentanol | 747 | 158.8 | 1.5233 | 0.20±0.02^a^ | n.d. | n.d. | n.d. | n.d. | n.d. | n.d. |
|  | **KETONES** |  |  |  | **21.47±0.03^g^** | **33.34±0.25^f^** | **40.42±0.06^d^** | **35.23±0.32^e^** | **44.01±0.33^b^** | **43.18±0.17^c^** | **48.08±0.20^a^** |
| 1 | 2-Heptanone (M) | 901 | 355.9 | 1.2607 | 5.34±0.19^b^ | 4.76±0.11^c^ | 6.07±0.06^a^ | 4.81±0.07^c^ | 5.87±0.05^a^ | 5.45±0.03^b^ | 4.50±0.04^d^ |
| 2 | 2-Heptanone (D) | 897 | 349.6 | 1.6349 | 2.05±0.08^f^ | 6.88±0.08^e^ | 10.99±0.11^d^ | 10.98±0.04^d^ | 12.29±0.16^c^ | 12.79±0.16^b^ | 14.73±0.14^a^ |
| 3 | 2-Pentanone(M) | 670 | 84.1 | 1.1220 | 2.24±0.08^d^ | 3.87±0.08^a^ | 2.79±0.04^c^ | 2.27±0.03^d^ | 3.04±0.03^b^ | 2.21±0.16^d^ | 2.32±0.06^d^ |
| 4 | 2-Pentanone(D) | 697 | 106.2 | 1.3687 | 3.87±0.06^e^ | 9.46±0.14^d^ | 12.33±0.004^b^ | 10.73±0.36^c^ | 11.97±0.42^b^ | 10.26±0.20^c^ | 13.00±0.05^a^ |
| 5 | 2, 3-Pentanedione (M) | 697 | 106.2 | 1.2261 | n.d. | 1.71±0.03^a^ | 0.61±0.01^c^ | 0.82±0.01^b^ | 0.61±0.03^c^ | 0.47±0.004^d^ | 0.27±0.005^e^ |
| 6 | 2, 3-Pentanedione (D) | 656 | 75.5 | 1.2401 | n.d. | 0.47±0.01^d^ | 0.66±0.004^a^ | 0.50±0.004^c^ | 0.42±0.003^e^ | 0.38±0.01^f^ | 0.60±0.03^b^ |
| 7 | 2-Butanone | 595 | 42.1 | 1.2456 | 0.34±0.002^f^ | 0.47±0.01^e^ | 0.79±0.01^b^ | 0.61±0.02^d^ | 0.79±0.01^b^ | 0.66±0.03^c^ | 0.87±0.04^a^ |
| 8 | 1-Penten-3-one | 672 | 85.2 | 1.0783 | n.d. | 0.93±0.02^a^ | 0.35±0.005^b^ | 0.32±0.01^c^ | 0.20±0.02^d^ | 0.23±0.002^d^ | 0.11±0.0004^e^ |
| 9 | 2-Octanone | 1005 | 558.5 | 1.3372 | n.d. | 0.94±0.01^a^ | 0.08±0.005^f^ | 0.22±0.01^d^ | 0.28±0.01^b^ | 0.14±0.001^e^ | 0.26±0.01^c^ |
| 10 | 2-Methyltetrahydrofuran-3-one | 808 | 227.2 | 1.4296 | 1.85±0.09^d^ | 0.01±0.001^e^ | 0.17±0.01^e^ | 0.18±0.01^e^ | 4.12±0.13^c^ | 5.52±0.14^b^ | 5.95±0.04^a^ |
| 11 | 3-Hexanone | 796 | 214.8 | 1.4787 | n.d. | 0.23±0.01^e^ | 0.44±0.02^b^ | 0.36±0.01^c^ | 0.36±0.01^c^ | 0.26±0.02^d^ | 0.47±0.01^a^ |
| 12 | Acetoin | 741 | 146.1 | 1.3302 | 0.07±0.001^e^ | 0.20±0.005^d^ | 0.22±0.01^d^ | 0.26±0.01^cd^ | 0.31±0.02^c^ | 1.58±0.04^a^ | 1.21±0.04^b^ |
| 13 | 3-Penten-2-one | 748 | 153.7 | 1.3475 | n.d. | 0.20±0.01^d^ | 0.30±0.003^c^ | 0.19±0.005^d^ | 0.28±0.01^c^ | 0.80±0.05^b^ | 0.87±0.01^a^ |
| 14 | 2,3-Butanedione | 613 | 49.5 | 1.1788 | 3.81±0.19^b^ | 3.23±0.12^cd^ | 4.61±0.12^a^ | 2.98±0.05^de^ | 3.48±0.01^c^ | 2.45±0.06^f^ | 2.91±0.04^e^ |
| 15 | Hydroxyacetone | 659 | 77.2 | 1.2421 | 0.25±0.01^a^ | n.d. | n.d. | n.d. | n.d. | n.d. | n.d. |
| 16 | 4-Methyl-2-pentanone | 706 | 116.0 | 1.1840 | 1.64±0.07^a^ | n.d. | n.d. | n.d. | n.d. | n.d. | n.d. |
|  | **ESTERS** |  |  |  | **29.81±0.16^a^** | **11.17±0.30^b^** | **8.90±0.03^c^** | **8.46±0.14^d^** | **6.12±0.04^f^** | **6.92±0.02^e^** | **3.90±0.09^g^** |
| 1 | Ethyl acetate(M) | 630 | 58.0 | 1.0972 | 5.16±0.06^a^ | 0.79±0.08^c^ | 0.93±0.03^b^ | 0.59±0.05^d^ | 0.52±0.01^d^ | 0.35±0.01^e^ | 0.34±0.003^e^ |
| 2 | Ethyl acetate(D) | 622 | 53.8 | 1.3362 | 6.15±0.14^a^ | 2.77±0.06^c^ | 3.50±0.08^b^ | 2.66±0.09^c^ | 0.39±0.004^e^ | 1.19±0.02^d^ | 0.39±0.04^e^ |
| 3 | Ethyl crotonate | 807 | 226.5 | 1.5568 | n.d. | 0.18±0.001^a^ | 0.11±0.005^c^ | 0.14±0.01^b^ | 0.07±0.001^d^ | 0.05±0.0003^f^ | 0.06±0.001^e^ |
| 4 | Isoamyl N-butyrate | 1069 | 739.3 | 1.4074 | n.d. | 0.72±0.04^b^ | 0.71±0.01^b^ | 0.62±0.01^c^ | 0.71±0.007^b^ | 0.95±0.005^a^ | 0.39±0.01^d^ |
| 5 | Ethyl butanoate | 807 | 226.8 | 1.2014 | 0.76±0.01^b^ | 0.98±0.01^a^ | 0.49±0.05^d^ | 0.56±0.01^c^ | 0.33±0.001^e^ | 0.47±0.003^d^ | 0.23±0.01^f^ |
| 6 | Pentyl acetate | 902 | 357.7 | 1.3160 | n.d. | 0.18±0.001^e^ | 0.28±0.03^c^ | 0.24±0.003^d^ | 0.26±0.001^cd^ | 0.45±0.003^b^ | 0.52±0.005^a^ |
| 7 | Methyl acetate | 854 | 284.2 | 1.0385 | 2.18±0.03^e^ | 5.55±0.33^a^ | 2.88±0.09^d^ | 3.65±0.07^bc^ | 3.84±0.03^b^ | 3.45±0.01^c^ | 1.97±0.09^e^ |
| 8 | Propyl acetate(M) | 723 | 132.2 | 1.1642 | 3.46±0.03^a^ | n.d. | n.d. | n.d. | n.d. | n.d. | n.d. |
| 9 | Propyl acetate(D) | 722 | 131.9 | 1.4752 | 4.70±0.12^a^ | n.d. | n.d. | n.d. | n.d. | n.d. | n.d. |

**Table S2.** (continued)

| No | Compounds | RI | RT (s) | DT (ms) | Relative content (%) | | | | | | |
| --- | --- | --- | --- | --- | --- | --- | --- | --- | --- | --- | --- |
|  |  |  |  |  | **Y1** | **P1** | **P2** | **P3** | **L1** | **L2** | **L3** |
| 10 | Ethyl acrylate | 703 | 112.3 | 1.4178 | 2.50±0.03^a^ | n.d. | n.d. | n.d. | n.d. | n.d. | n.d. |
| 11 | Pentyl butanoate | 1093 | 730.3 | 1.4086 | 0.23±0.01^a^ | n.d. | n.d. | n.d. | n.d. | n.d. | n.d. |
| 12 | Ethyl formate | 621 | 55.0 | 1.0559 | 0.84±0.11^a^ | n.d. | n.d. | n.d. | n.d. | n.d. | n.d. |
| 13 | Ethyl (methylthio)acetate | 1005 | 559.5 | 1.5933 | n.d. | n.d. | n.d. | n.d. | n.d. | n.d. | n.d. |
| 14 | Ethyl butyrate | 1111 | 886.8 | 1.6135 | n.d. | n.d. | n.d. | n.d. | n.d. | n.d. | n.d. |
| 15 | Ethyl propanoate | 711 | 120.3 | 1.4425 | 0.27±0.0008^a^ | n.d. | n.d. | n.d. | n.d. | n.d. | n.d. |
| 16 | Isobutyl acetate | 744 | 155.3 | 1.5946 | 0.30±0.003^a^ | n.d. | n.d. | n.d. | n.d. | n.d. | n.d. |
| 17 | Ethyl isobutyrate | 743 | 154.8 | 1.5485 | 0.19±0.004^a^ | n.d. | n.d. | n.d. | n.d. | n.d. | n.d. |
| 18 | Isopropyl acetate | 654 | 73.7 | 1.1779 | 3.09±0.10^a^ | n.d. | n.d. | n.d. | n.d. | n.d. | n.d. |
|  | **OLEFINS** |  |  |  | **0.43±0.04^g^** | **4.35±0.14^f^** | **8.68±0.24^b^** | **12.61±0.03^a^** | **8.31±0.19^c^** | **5.43±0.03^e^** | **5.90±0.04^d^** |
| 1 | Limonene | 1008 | 559.4 | 1.279 | n.d. | n.d. | n.d. | n.d. | n.d. | n.d. | n.d. |
| 2 | *α*-Phellandrene | 1012 | 575.8 | 1.6712 | n.d. | 1.65±0.06^f^ | 7.90±0.20^b^ | 12.02±0.04^a^ | 7.13±0.15^c^ | 4.22±0.02^e^ | 5.39±0.05^d^ |
| 3 | 1-Heptene | 744 | 149.7 | 1.0782 | n.d. | 1.72±0.02^a^ | 0.09±0.002^f^ | 0.24±0.002^d^ | 0.34±0.005^c^ | 0.55±0.004^b^ | 0.15±0.002^e^ |
| 4 | *β*-Pinene | 967 | 475.1 | 1.2162 | n.d. | 0.81±0.05^a^ | 0.57±0.06^c^ | 0.16±0.005^e^ | 0.70±0.04^b^ | 0.47±0.004^d^ | 0.12±0.002^e^ |
| 5 | 1-Octene | 824 | 246.0 | 1.4613 | 0.35±0.04^a^ | 0.18±0.01^cd^ | 0.12±0.01^e^ | 0.18±0.02^cd^ | 0.14±0.005^de^ | 0.20±0.001^bc^ | 0.24±0.001^b^ |
| 6 | Myrcene | 961 | 466.4 | 1.2118 | 0.08±0.0002^a^ | n.d. | n.d. | n.d. | n.d. | n.d. | n.d. |
|  | **ALKANES** |  |  |  | **1.49±0.07^e^** | **2.26±0.18^c^** | **1.24±0.03^f^** | **1.83±0.04^d^** | **1.63±0.08^de^** | **3.50±0.07^b^** | **5.25±0.07^a^** |
| 1 | 2,2,4,6,6-Pentamethylheptane(M) | 1003 | 555.7 | 1.0387 | 1.49±0.07^ab^ | 1.68±0.21^a^ | 0.95±0.03^c^ | 0.92±0.07^c^ | 1.13±0.09^c^ | 1.41±0.03^b^ | 0.94±0.04^c^ |
| 2 | 2,2,4,6,6-Pentamethylheptane(D) | 911 | 372.3 | 1.3667 | n.d. | 0.38±0.04^b^ | 0.07±0.001^d^ | 0.33±0.01^c^ | 0.35±0.01^bc^ | 0.45±0.001^a^ | 0.46±0.01^a^ |
| 3 | Undecane | 1094 | 823.7 | 1.1628 | n.d. | 0.20±0.01^d^ | 0.22±0.004^d^ | 0.57±0.02^c^ | 0.15±0.004^d^ | 1.64±0.06^b^ | 3.85±0.09^a^ |
|  | **HETEROCYCLIC** |  |  |  | **2.49±0.13^d^** | **2.59±0.05^d^** | **3.59±0.13^b^** | **6.21±0.09^a^** | **3.65±0.04^b^** | **3.74±0.03^b^** | **3.27±0.09^c^** |
| 1 | 2-Methylpyrazine | 800 | 226.7 | 1.3832 | n.d. | n.d. | n.d. | n.d. | n.d. | n.d. | n.d. |
| 2 | 1-(Furan-2-yl)ethanone | 897 | 349.4 | 1.4458 | n.d. | n.d. | n.d. | n.d. | n.d. | n.d. | n.d. |
| 3 | 2-Isobutyl-3-methylpyrazine | 1112 | 890.9 | 1.8084 | n.d. | 0.22±0.05^e^ | 1.66±0.12^c^ | 4.70±0.10^a^ | 2.11±0.06^b^ | 0.65±0.01^d^ | 1.84±0.09^c^ |
| 4 | 4-Methylthiazole | 804 | 223.5 | 1.3526 | n.d. | 0.20±0.02^c^ | 0.17±0.004^cd^ | 0.17±0.003^d^ | 0.14±0.001^e^ | 1.15±0.01^a^ | 0.56±0.01^b^ |
| 5 | Pyrazine | 729 | 133.5 | 1.2746 | n.d. | 0.86±0.01^a^ | 0.06±0.0009^e^ | 0.14±0.01^d^ | 0.13±0.003^d^ | 0.39±0.003^b^ | 0.19±0.003^c^ |
| 6 | dms | 758 | 165.6 | 0.9615 | n.d. | 0.82±0.05^c^ | 1.15±0.05^a^ | 0.80±0.02^c^ | 1.07±0.02^b^ | 0.55±0.004^d^ | 0.27±0.01^e^ |
| 7 | 2,6-Dimethylpyrazine | 905 | 361.9 | 1.5456 | n.d. | 0.49±0.02^c^ | 0.54±0.01^b^ | 0.41±0.002^d^ | 0.21±0.004^e^ | 1.00±0.02^a^ | 0.41±0.004^d^ |

**Table S2.** (continued)

| No | Compounds | RI | RT (s) | DT (ms) | Relative content (%) | | | | | | |
| --- | --- | --- | --- | --- | --- | --- | --- | --- | --- | --- | --- |
|  |  |  |  |  | **Y1** | **P1** | **P2** | **P3** | **L1** | **L2** | **L3** |
| 8 | N-nitrosodiethylamine | 891 | 340.1 | 1.5405 | 0.15±0.01^a^ | n.d. | n.d. | n.d. | n.d. | n.d. | n.d. |
| 9 | 2-Isobutyl-3-methoxypyrazine | 1173 | 891.1 | 1.8243 | 2.34±0.13^a^ | n.d. | n.d. | n.d. | n.d. | n.d. | n.d. |

The results were expressed as mean ± standard deviation (n = 3). Mean values with different lowercase letters in the same row are significantly different at *p* < 0.05. M, monomer; D, dimer. ^1^RI, retention index; ^2^Rt, retention time; ^3^Dt, drift time.

**Table S3.** HS-GC-IMS integration parameters of VOCs in lipase-catalyzed alcoholized butter.

| No | Compounds | RI | RT (s) | DT (ms) | Relative content (%) | | | | | |
| --- | --- | --- | --- | --- | --- | --- | --- | --- | --- | --- |
|  |  |  |  |  | EP1 | EP2 | EP3 | EL1 | EL2 | EL3 |
|  | **ACIDS** |  |  |  | **4.40±0.17^d^** | **8.53±0.18^a^** | **8.05±0.08^b^** | **3.48±0.13^f^** | **3.62±0.18^f^** | **6.00±0.13^c^** |
| 1 | Acetic acid | 587 | 37.2 | 1.0393 | n.d. | n.d. | n.d. | n.d. | n.d. | n.d. |
| 2 | Butyric Acid | 813 | 233 | 1.1658 | 2.81±0.10^c^ | 5.77±0.16^a^ | 5.33±0.06^b^ | 1.29±0.06^d^ | 0.44±1.60^d^ | 1.21±0.08^d^ |
| 3 | Propionic acid | 704 | 111.8 | 1.0975 | 0.44±0.02^e^ | 1.87±0.02^b^ | 1.69±0.02^c^ | 0.60±0.03^e^ | 1.32±0.12^d^ | 4.36±0.19^a^ |
| 4 | 2-Methylbutyric acid | 899 | 352.9 | 1.2115 | 1.16±0.07^b^ | 0.89±0.03^c^ | 1.02±0.04^bc^ | 1.59±0.06^a^ | 0.96±0.04^c^ | 0.43±0.18^d^ |
| 5 | 1-Hexanoic acid | 1022 | 602.8 | 1.2947 | n.d. | n.d. | n.d. | n.d. | n.d. | n.d. |
|  | **ALDEHYDES** |  |  |  | **16.67±0.64^a^** | **11.35±0.16^b^** | **11.71±0.15^b^** | **11.48±0.16^b^** | **11.58±0.50^b^** | **9.67±0.44^c^** |
| 1 | Valeraldehyde | 724 | 128.9 | 1.4224 | n.d. | n.d. | n.d. | n.d. | n.d. | n.d. |
| 2 | 2-Methylbutanal | 696 | 105.5 | 1.3966 | n.d. | n.d. | n.d. | n.d. | n.d. | n.d. |
| 3 | Octanal | 1007 | 558.3 | 1.3983 | 0.98±0.03^a^ | 0.97±0.03^a^ | 0.99±0.03^a^ | 0.11±0.002^b^ | 0.08±0.01^b^ | 0.03±0.001^c^ |
| 4 | Furfural | 804 | 223.2 | 1.3373 | 0.41±0.01^d^ | 0.46±0.04^d^ | 1.11±0.10^a^ | 0.40±0.04^d^ | 0.62±0.01^c^ | 0.89±0.16^b^ |
| 5 | 3-Methyl-2-butenal | 784 | 200.1 | 1.0959 | 11.12±0.62^a^ | 7.10±0.30^c^ | 6.87±0.46^c^ | 8.48±0.24^b^ | 8.88±0.27^b^ | 7.16±0.53^c^ |
| 6 | N-Methylpyrrole-2-carboxaldehyde | 1011 | 575.3 | 1.4937 | 1.20±0.04^d^ | 0.99±0.02^e^ | 1.01±0.04^e^ | 2.26±0.07^a^ | 1.95±0.19^b^ | 1.52±0.05^c^ |
| 7 | Butanal | 614 | 50.1 | 1.2914 | n.d. | n.d. | n.d. | n.d. | n.d. | n.d. |
| 8 | Isovaleraldehyde | 632 | 59.2 | 1.1961 | n.d. | n.d. | n.d. | n.d. | n.d. | n.d. |
| 9 | 2-Methylpropenal | 575 | 34.8 | 1.2258 | n.d. | n.d. | n.d. | n.d. | n.d. | n.d. |
| 10 | Hexanal | 804 | 223 | 1.2534 | 2.04±0.09^a^ | 1.14±0.24^b^ | 0.95±0.23^b^ | 0.09±0.01^c^ | 0.06±0.002^c^ | 0.04±0.01^c^ |
| 11 | Propanal | 756 | 163.4 | 1.04 | n.d. | n.d. | n.d. | n.d. | n.d. | n.d. |
| 12 | Acetaldehyde | 749 | 160.9 | 0.9577 | n.d. | n.d. | n.d. | n.d. | n.d. | n.d. |
| 13 | Nonanal | 1101 | 745.6 | 1.4805 | n.d. | n.d. | n.d. | n.d. | n.d. | n.d. |
| 14 | Isobutyraldehyde | 552 | 19.5 | 1.1144 | n.d. | n.d. | n.d. | n.d. | n.d. | n.d. |
| 15 | Heptanal | 900 | 355.5 | 1.3335 | 0.93±0.03^a^ | 0.70±0.07^c^ | 0.78±0.05^b^ | 0.14±0.01^d^ | 0.09±0.01^de^ | 0.04±0.01^e^ |
| 16 | 2-Methylpentanal | 755 | 167.7 | 1.216 | n.d. | n.d. | n.d. | n.d. | n.d. | n.d. |
|  | **ALCOHOLS** |  |  |  | **n.d.** | **n.d.** | **n.d.** | **n.d.** | **n.d.** | **n.d.** |
| 1 | 2-Methylbutan-1-ol | 732 | 137.1 | 1.4771 | n.d. | n.d. | n.d. | n.d. | n.d. | n.d. |
| 2 | Ethanol | 964 | 467.4 | 1.0409 | n.d. | n.d. | n.d. | n.d. | n.d. | n.d. |
| 3 | 1-Propanol | 565 | 31.9 | 1.1129 | n.d. | n.d. | n.d. | n.d. | n.d. | n.d. |
| 4 | 3-Methyl-1-butanol | 767 | 176.4 | 1.1190 | n.d. | n.d. | n.d. | n.d. | n.d. | n.d. |
| 5 | Isoprenol | 734 | 138.7 | 1.1646 | n.d. | n.d. | n.d. | n.d. | n.d. | n.d. |
| 6 | Methanethiol | 641 | 64.2 | 1.0398 | n.d. | n.d. | n.d. | n.d. | n.d. | n.d. |

**Table S3.** (continued)

| No | Compounds | RI | RT (s) | DT (ms) | Relative content (%) | | | | | |
| --- | --- | --- | --- | --- | --- | --- | --- | --- | --- | --- |
|  |  |  |  |  | **EP1** | **EP2** | **EP3** | **EL1** | **EL2** | **EL3** |
| 7 | 2-Methylpropanol | 1132 | 970.5 | 1.1707 | n.d. | n.d. | n.d. | n.d. | n.d. | n.d. |
| 8 | 1,3-Butanediol | 784 | 201 | 1.1240 | n.d. | n.d. | n.d. | n.d. | n.d. | n.d. |
| 9 | 2-Ethylhexanol | 1039 | 622.8 | 1.4093 | n.d. | n.d. | n.d. | n.d. | n.d. | n.d. |
| 10 | Isopentanol | 747 | 158.8 | 1.5233 | n.d. | n.d. | n.d. | n.d. | n.d. | n.d. |
|  | **KETONES** |  |  |  | **21.69±1.52^a^** | **15.39±0.45^b^** | **14.44±1.78^b^** | **0.42±0.02^c^** | **0.95±0.07^c^** | **0.99±0.14^c^** |
| 1 | 2-Heptanone (M) | 901 | 355.9 | 1.2607 | n.d. | n.d. | n.d. | n.d. | n.d. | n.d. |
| 2 | 2-Heptanone (D) | 897 | 349.6 | 1.6349 | n.d. | n.d. | n.d. | n.d. | n.d. | n.d. |
| 3 | 2-Pentanone(M) | 670 | 84.1 | 1.1220 | n.d. | n.d. | n.d. | n.d. | n.d. | n.d. |
| 4 | 2-Pentanone(D) | 697 | 106.2 | 1.3687 | 0.32±0.03^b^ | 0.41±0.07^b^ | 1.02±0.05^a^ | 0.42±0.02^b^ | 0.96±0.06^a^ | 0.99±0.14^a^ |
| 5 | 2, 3-Pentanedione (M) | 697 | 106.2 | 1.2261 | n.d. | n.d. | n.d. | n.d. | n.d. | n.d. |
| 6 | 2, 3-Pentanedione (D) | 656 | 75.5 | 1.2401 | n.d. | n.d. | n.d. | n.d. | n.d. | n.d. |
| 7 | 2-Butanone | 595 | 42.1 | 1.2456 | n.d. | n.d. | n.d. | n.d. | n.d. | n.d. |
| 8 | 1-Penten-3-one | 672 | 85.2 | 1.0783 | n.d. | n.d. | n.d. | n.d. | n.d. | n.d. |
| 9 | 2-Octanone | 1005 | 558.5 | 1.3372 | n.d. | n.d. | n.d. | n.d. | n.d. | n.d. |
| 10 | 2-Methyltetrahydrofuran-3-one | 808 | 227.2 | 1.4296 | n.d. | n.d. | n.d. | n.d. | n.d. | n.d. |
| 11 | 3-Hexanone | 796 | 214.8 | 1.4787 | n.d. | n.d. | n.d. | n.d. | n.d. | n.d. |
| 12 | Acetoin | 741 | 146.1 | 1.3302 | n.d. | n.d. | n.d. | n.d. | n.d. | n.d. |
| 13 | 3-Penten-2-one | 748 | 153.7 | 1.3475 | n.d. | n.d. | n.d. | n.d. | n.d. | n.d. |
| 14 | 2,3-Butanedione | 613 | 49.5 | 1.1788 | 21.37±1.49^a^ | 14.97±0.41^b^ | 13.42±1.82^b^ | n.d. | n.d. | n.d. |
| 15 | Hydroxyacetone | 659 | 77.2 | 1.2421 | n.d. | n.d. | n.d. | n.d. | n.d. | n.d. |
| 16 | 4-Methyl-2-pentanone | 706 | 116.0 | 1.1840 | n.d. | n.d. | n.d. | n.d. | n.d. | n.d. |
|  | **ESTERS** |  |  |  | **35.49±2.09^c^** | **25.66±0.59^e^** | **28.78±1.30^d^** | **52.67±0.64^a^** | **44.57±2.07^b^** | **36.26±0.64^c^** |
| 1 | Ethyl acetate(M) | 630 | 58.0 | 1.0972 | n.d. | n.d. | n.d. | n.d. | n.d. | n.d. |
| 2 | Ethyl acetate(D) | 622 | 53.8 | 1.3362 | n.d. | n.d. | n.d. | n.d. | n.d. | n.d. |
| 3 | Ethyl crotonate | 807 | 226.5 | 1.5568 | n.d. | n.d. | n.d. | n.d. | n.d. | n.d. |
| 4 | Isoamyl N-butyrate | 1069 | 739.3 | 1.4074 | n.d. | n.d. | n.d. | n.d. | n.d. | n.d. |
| 5 | Ethyl butanoate | 807 | 226.8 | 1.2014 | n.d. | n.d. | n.d. | n.d. | n.d. | n.d. |
| 6 | Pentyl acetate | 902 | 357.7 | 1.3160 | n.d. | n.d. | n.d. | n.d. | n.d. | n.d. |
| 7 | Methyl acetate | 854 | 284.2 | 1.0385 | n.d. | n.d. | n.d. | n.d. | n.d. | n.d. |
| 8 | Propyl acetate(M) | 723 | 132.2 | 1.1642 | n.d. | n.d. | n.d. | n.d. | n.d. | n.d. |
| 9 | Propyl acetate(D) | 722 | 131.9 | 1.4752 | n.d. | n.d. | n.d. | n.d. | n.d. | n.d. |

**Table S3.** (continued)

| No | Compounds | RI | RT (s) | DT (ms) | Relative content (%) | | | | | |
| --- | --- | --- | --- | --- | --- | --- | --- | --- | --- | --- |
|  |  |  |  |  | **EP1** | **EP2** | **EP3** | **EL1** | **EL2** | **EL3** |
| 10 | Ethyl acrylate | 703 | 112.3 | 1.4178 | n.d. | n.d. | n.d. | n.d. | n.d. | n.d. |
| 11 | Pentyl butanoate | 1093 | 730.3 | 1.4086 | n.d. | n.d. | n.d. | n.d. | n.d. | n.d. |
| 12 | Ethyl formate | 621 | 55.0 | 1.0559 | n.d. | n.d. | n.d. | n.d. | n.d. | n.d. |
| 13 | Ethyl (methylthio)acetate | 1005 | 559.5 | 1.5933 | 1.32±0.04^d^ | 0.86±0.06^d^ | 4.01±0.27^c^ | 7.93±0.09^a^ | 5.90±0.66^b^ | 6.46±0.37^b^ |
| 14 | Ethyl butyrate | 1111 | 886.8 | 1.6135 | 25.35±2.43^b^ | 19.02±0.37^d^ | 17.38±0.75^d^ | 28.30±0.44^a^ | 27.25±2.40^ab^ | 21.93±0.66^c^ |
| 15 | Ethyl propanoate | 711 | 120.3 | 1.4425 | 8.83±0.34^c^ | 5.78±0.58^e^ | 7.39±0.36^d^ | 16.44±1.13^a^ | 11.82±0.66^b^ | 7.87±1.10^cd^ |
| 16 | Isobutyl acetate | 744 | 155.3 | 1.5946 | n.d. | n.d. | n.d. | n.d. | n.d. | n.d. |
| 17 | Ethyl isobutyrate | 743 | 154.8 | 1.5485 | n.d. | n.d. | n.d. | n.d. | n.d. | n.d. |
| 18 | Isopropyl acetate | 654 | 73.7 | 1.1779 | n.d. | n.d. | n.d. | n.d. | n.d. | n.d. |
|  | **OLEFINS** |  |  |  | **2.22±0.06^d^** | **5.46±0.51^b^** | **4.91±0.38^bc^** | **1.53±0.06^d^** | **4.04±0.93^c^** | **7.55±0.71^a^** |
| 1 | Limonene | 1008 | 559.4 | 1.279 | 2.22±0.06^d^ | 5.46±0.51^b^ | 4.91±0.38^bc^ | 1.53±0.06^d^ | 4.07±0.88^c^ | 7.55±0.71^a^ |
| 2 | *α*-Phellandrene | 1012 | 575.8 | 1.6712 | n.d. | n.d. | n.d. | n.d. | n.d. | n.d. |
| 3 | 1-Heptene | 744 | 149.7 | 1.0782 | n.d. | n.d. | n.d. | n.d. | n.d. | n.d. |
| 4 | *β*-Pinene | 967 | 475.1 | 1.2162 | n.d. | n.d. | n.d. | n.d. | n.d. | n.d. |
| 5 | 1-Octene | 824 | 246.0 | 1.4613 | n.d. | n.d. | n.d. | n.d. | n.d. | n.d. |
| 6 | Myrcene | 961 | 466.4 | 1.2118 | n.d. | n.d. | n.d. | n.d. | n.d. | n.d. |
|  | **ALKANES** |  |  |  | **n.d.** | **n.d.** | **n.d.** | **n.d.** | **n.d.** | **n.d.** |
| 1 | 2,2,4,6,6-Pentamethylheptane(M) | 1003 | 555.7 | 1.0387 | n.d. | n.d. | n.d. | n.d. | n.d. | n.d. |
| 2 | 2,2,4,6,6-Pentamethylheptane(D) | 911 | 372.3 | 1.3667 | n.d. | n.d. | n.d. | n.d. | n.d. | n.d. |
| 3 | Undecane | 1094 | 823.7 | 1.1628 | n.d. | n.d. | n.d. | n.d. | n.d. | n.d. |
|  | **HETEROCYCLIC** |  |  |  | **19.52±0.56^e^** | **33.61±0.54^bc^** | **32.11±0.33^cd^** | **30.41±0.52^d^** | **35.24±3.50^b^** | **39.53±0.17^a^** |
| 1 | 2-Methylpyrazine | 800 | 226.7 | 1.3832 | 19.19±0.54^d^ | 33.34±0.54^b^ | 31.14±0.39^bc^ | 28.94±0.32^c^ | 33.63±3.95^b^ | 37.89±0.18^a^ |
| 2 | 1-(Furan-2-yl)ethanone | 897 | 349.4 | 1.4458 | 0.33±0.02^d^ | 0.28±0.003^d^ | 0.97±0.10^c^ | 1.47±0.22^b^ | 1.96±0.07^a^ | 1.64±0.08^b^ |
| 3 | 2-Isobutyl-3-methylpyrazine | 1112 | 890.9 | 1.8084 | n.d. | n.d. | n.d. | n.d. | n.d. | n.d. |
| 4 | 4-Methylthiazole | 804 | 223.5 | 1.3526 | n.d. | n.d. | n.d. | n.d. | n.d. | n.d. |
| 5 | Pyrazine | 729 | 133.5 | 1.2746 | n.d. | n.d. | n.d. | n.d. | n.d. | n.d. |
| 6 | dms | 758 | 165.6 | 0.9615 | n.d. | n.d. | n.d. | n.d. | n.d. | n.d. |
| 7 | 2,6-Dimethylpyrazine | 905 | 361.9 | 1.5456 | n.d. | n.d. | n.d. | n.d. | n.d. | n.d. |
| 8 | N-nitrosodiethylamine | 891 | 340.1 | 1.5405 | n.d. | n.d. | n.d. | n.d. | n.d. | n.d. |

**Table S3.** (continued)

| No | Compounds | RI | RT (s) | DT (ms) | Relative content (%) | | | | | |
| --- | --- | --- | --- | --- | --- | --- | --- | --- | --- | --- |
|  |  |  |  |  | **EP1** | **EP2** | **EP3** | **EL1** | **EL2** | **EL3** |
| 9 | 2-Isobutyl-3-methoxypyrazine | 1173 | 891.1 | 1.8243 | n.d. | n.d. | n.d. | n.d. | n.d. | n.d. |

The results were expressed as mean ± standard deviation (n = 3). Mean values with different lowercase letters in the same row are significantly different at *p* < 0.05.

**Table S4.** ROAV of VOCs in raw and lipase-catalyzed hydrolyzed butter.

| No. | Compounds | Odor characteristics^1^ | Threshold (mg/kg)^2^ | ROAV | | | | | | |
| --- | --- | --- | --- | --- | --- | --- | --- | --- | --- | --- |
|  |  |  |  | **Y1** | **P1** | **P2** | **P3** | **L1** | **L2** | **L3** |
| 1 | Acetic acid | Acid, Fruit, Pungent, Sour, Vinegar | 22 | 0.0001 | n.d. | n.d. | n.d. | n.d. | n.d. | n.d. |
| 2 | 1-Hexanoic acid | Coconut meat and cheese | 3 | 0.01 | 0.03 | 0.03 | 0.03 | 0.01 | 0.01 | 0.01 |
| 3 | Butyric Acid | Yogurt | 0.1 | n.d. | n.d. | n.d. | n.d. | n.d. | n.d. | n.d. |
| 4 | Propionic acid | Acidic cheese, vinegar | 0.1 | n.d. | n.d. | n.d. | n.d. | n.d. | n.d. | n.d. |
| 5 | 2-Methylbutyric acid | Acidic goat milk cheese | 0.01 | n.d. | n.d. | n.d. | n.d. | n.d. | n.d. | n.d. |
| 6 | Valeraldehyde | Fermented bread and fruit | 0.012 | n.d. | 19.97 | 22.61 | 27.29 | 23.47 | 47.18 | 49.52 |
| 7 | 2-Methylbutanal | Fermented and baked | 0.0024 | 0.67 | 36.52 | 22.75 | 21.94 | 15.61 | 60.24 | 26.00 |
| 8 | Butanal | Pungent | 0.009 | 0.64 | 25.66 | 28.21 | 34.00 | 11.37 | 30.80 | 23.70 |
| 9 | Isovaleraldehyde | Chocolate and peach | 0.0024 | n.d. | 52.43 | 12.90 | 25.33 | 11.35 | 29.89 | 9.92 |
| 10 | 2-Methylpropenal | Wild hyacinth | 0.025 | 0.26 | 4.33 | 5.67 | 6.33 | 3.62 | 6.22 | 5.71 |
| 11 | Hexanal | Oil and grass | 0.0045 | 0.59 | 12.48 | 8.21 | 9.26 | 6.70 | 16.90 | 4.79 |
| 12 | Propanal | Soil and nut | 0.0151 | n.d. | 58.83 | 23.76 | 35.85 | 20.95 | 58.29 | 37.26 |
| 13 | Acetaldehyde | Floral, Green Apple | 0.025 | 0.65 | n.d. | n.d. | n.d. | n.d. | n.d. | n.d. |
| 14 | Nonanal | Fat, Floral, Green, Lemon | 0.001 | 9.51 | n.d. | n.d. | n.d. | n.d. | n.d. | n.d. |
| 15 | Isobutyraldehyde | Burnt, Caramel, Cocoa, Green, Malt | 0.0004 | 100.00 | n.d. | n.d. | n.d. | n.d. | n.d. | n.d. |
| 16 | Heptanal | Citrus, Fat, Green, Nut | 0.003 | 0.61 | n.d. | n.d. | n.d. | n.d. | n.d. | n.d. |
| 17 | Octanal | Fat, sweet orange | 0.0034 | n.d. | n.d. | n.d. | n.d. | n.d. | n.d. | n.d. |
| 18 | Furfural | Almonds, toasted bread | 3 | n.d. | n.d. | n.d. | n.d. | n.d. | n.d. | n.d. |
| 19 | N-Methylpyrrole-2-carboxaldehyde | roasted nuts | 0.037 | n.d. | n.d. | n.d. | n.d. | n.d. | n.d. | n.d. |
| 20 | 2-Methylpentanal | Green, Fruit | 0.0016 | 0.03 | n.d. | n.d. | n.d. | n.d. | n.d. | n.d. |
| 21 | 2-Methylbutan-1-ol | Alcohol | 0.25 | n.d. | 0.16 | 0.23 | 0.24 | 0.22 | 0.78 | 0.57 |
| 22 | Ethanol | Alcohol | 0.025 | n.d. | 30.27 | 12.82 | 23.38 | 15.61 | 40.50 | 22.88 |
| 23 | 1-Propanol | Alcohol | 5.7 | n.d. | 0.20 | 0.19 | 0.24 | 0.21 | 0.32 | 0.36 |
| 24 | 3-Methyl-1-butanol | Slight alcohol | 0.25 | n.d. | 2.03 | 0.86 | 1.39 | 1.87 | 2.26 | 1.86 |
| 25 | Isoprenol | Sweet fruit and wine | 0.547125 | n.d. | 0.21 | 0.22 | 0.22 | 0.19 | 0.39 | 0.21 |
| 26 | Methanethiol | Garlic | 0.004 | 0.76 | 92.61 | 44.53 | 65.87 | 59.16 | 90.20 | 51.11 |
| 27 | 1,3-Butanediol | Bitter-sweet | 10 | 0.001 | n.d. | n.d. | n.d. | n.d. | n.d. | n.d. |
| 28 | 2-Ethylhexanol | Green, Rose | 0.83 | 0.004 | n.d. | n.d. | n.d. | n.d. | n.d. | n.d. |
| 29 | Isopentanol | Burnt, Cocoa, Floral, Malt | 0.25 | 0.002 | n.d. | n.d. | n.d. | n.d. | n.d. | n.d. |
| 30 | 2-Heptanone (M) | Fruit, herbal and wood | 0.14 | 0.10 | 3.47 | 3.77 | 4.32 | 3.92 | 7.06 | 5.51 |
| 31 | 2-Heptanone (D) | Fruit, herbal and wood | 0.14 | 0.04 | 5.03 | 6.81 | 9.88 | 8.21 | 16.56 | 18.05 |
| 32 | 2-Pentanone(M) | Sweet fruit, wine and woody | 1.38 | 0.17 | 0.29 | 0.18 | 0.21 | 0.21 | 0.29 | 0.29 |
| 33 | 2-Pentanone(D) | Sweet fruit, wine and wood | 1.38 | 0.29 | 0.70 | 0.78 | 0.98 | 0.81 | 1.35 | 1.62 |
| 34 | 2, 3-Pentanedione (M) | Butter and cream | 0.02 | n.d. | 8.74 | 2.63 | 5.20 | 2.87 | 4.22 | 2.28 |
| 35 | 2, 3-Pentanedione (D) | Butter and cream | 0.02 | n.d. | 2.39 | 2.86 | 3.15 | 1.96 | 3.42 | 5.14 |
| 36 | 2-Butanone | Fragrant and fruit | 17 | 0.00006 | 0.003 | 0.004 | 0.004 | 0.004 | 0.01 | 0.01 |
| 37 | 1-Penten-3-one | Spicy pepper | 0.023 | n.d. | 4.13 | 1.34 | 1.76 | 0.82 | 1.78 | 0.84 |
| 38 | 2-Octanone | Natural woody and herbal | 0.005 | n.d. | 19.34 | 1.49 | 5.57 | 5.27 | 4.94 | 8.93 |
| 39 | 3-Hexanone | Sweet fruit and wine | 0.041 | n.d. | 0.57 | 0.93 | 1.10 | 0.81 | 1.13 | 1.95 |

**Table S4.** (continued)

| No. | | Compounds | Odor characteristics | Threshold (mg/kg) | ROAV | | | | | | |
| --- | --- | --- | --- | --- | --- | --- | --- | --- | --- | --- | --- |
|  |  |  |  |  | **Y1** | **P1** | **P2** | **P3** | **L1** | **L2** | **L3** |
| 40 | Acetoin | | Butter | 8 | 0.00002 | 0.003 | 0.002 | 0.004 | 0.004 | 0.04 | 0.03 |
| 41 | 3-Penten-2-one | | Acetone | 0.0015 | n.d. | 13.19 | 17.26 | 15.73 | 17.24 | 96.90 | 100.00 |
| 42 | 2,3-Butanedione | | Rich butter and sweet cream | 0.0065 | 1.61 | 50.73 | 61.54 | 57.65 | 50.01 | 68.23 | 76.92 |
| 43 | Hydroxyacetone | | Butter, Herb, Malt, Pungent | 80 | 0.00001 | n.d. | n.d. | n.d. | n.d. | n.d. | n.d. |
| 44 | 4-Methyl-2-pentanone | | green | 0.24 | 0.02 | n.d. | n.d. | n.d. | n.d. | n.d. | n.d. |
| 45 | Ethyl (methylthio)acetate | | Fruit | 0.025 | n.d. | n.d. | n.d. | n.d. | n.d. | n.d. | n.d. |
| 46 | Ethyl butyrate | | Fruit | 0.066 | n.d. | n.d. | n.d. | n.d. | n.d. | n.d. | n.d. |
| 47 | n-Ethyl propanoate | | sweet fruits | 0.01 | n.d. | n.d. | n.d. | n.d. | n.d. | n.d. | n.d. |
| 48 | Ethyl acetate(M) | | Fruit | 5 | 0.003 | 0.02 | 0.02 | 0.01 | 0.01 | 0.01 | 0.01 |
| 49 | Ethyl acetate(D) | | Fruit | 5 | 0.003 | 0.06 | 0.06 | 0.07 | 0.01 | 0.04 | 0.01 |
| 50 | Isoamyl N-butyrate | | Fruit | 0.015 | n.d. | 4.90 | 4.10 | 5.22 | 4.39 | 11.55 | 4.46 |
| 51 | Butyl acetate | | Apple or pineapple | 0.001 | 2.08 | 100.00 | 42.78 | 70.72 | 30.74 | 84.82 | 38.86 |
| 52 | Pentyl acetate | | Banana | 0.0041 | n.d. | 4.56 | 6.06 | 7.42 | 6.03 | 19.98 | 22.03 |
| 53 | Methyl acetate | | Sweet | 1.5 | 0.004 | 0.38 | 0.17 | 0.31 | 0.24 | 0.42 | 0.23 |
| 54 | Propyl acetate(M) | | Celery, Floral, Pear, Red Fruit | 2.7 | 0.004 | n.d. | n.d. | n.d. | n.d. | n.d. | n.d. |
| 55 | Propyl acetate(D) | | Celery, Floral, Pear, Red Fruit | 2.7 | 0.005 | n.d. | n.d. | n.d. | n.d. | n.d. | n.d. |
| 56 | Ethyl acrylate | | Plastic, Fragrant, Pungent | 0.0067 | 1.02 | n.d. | n.d. | n.d. | n.d. | n.d. | n.d. |
| 57 | Pentyl butanoate | | Banana,fruity | 0.21 | 0.003 | n.d. | n.d. | n.d. | n.d. | n.d. | n.d. |
| 58 | Ethyl formate | | Pungent | 8.9 | 0.0003 | n.d. | n.d. | n.d. | n.d. | n.d. | n.d. |
| 59 | Ethyl propanoate | | Apple, Pineapple, Rum, Strawberry | 0.01 | 0.07 | n.d. | n.d. | n.d. | n.d. | n.d. | n.d. |
| 60 | Isobutyl acetate | | Apple, Banana, Floral, Herb | 0.44 | 0.002 | n.d. | n.d. | n.d. | n.d. | n.d. | n.d. |
| 61 | Ethyl isobutyrate | | fruity, wine | 0.0001 | 5.13 | n.d. | n.d. | n.d. | n.d. | n.d. | n.d. |
| 62 | Isopropyl acetate | | Banana | 1.7 | 0.005 | n.d. | n.d. | n.d. | n.d. | n.d. | n.d. |
| 63 | Myrcene | | Balsamic, Fruit, Geranium, Herb, Must | 0.015 | 0.01 | n.d. | n.d. | n.d. | n.d. | n.d. | n.d. |
| 64 | *α*-Phellandrene | | Citrus | 0.04 | n.d. | 4.21 | 17.13 | 37.85 | 16.68 | 19.10 | 23.11 |
| 65 | *β*-Pinene | | Eucalyptus camphor | 0.14 | n.d. | 0.59 | 0.36 | 0.15 | 0.47 | 0.60 | 0.15 |
| 66 | 1-Octene | | Gasoline smell | 0.0005 | 1.93 | 36.06 | 20.23 | 45.19 | 26.79 | 72.29 | 81.30 |
| 67 | 2-Isobutyl-3-methylpyrazine | | Syrup | 0.035 | n.d. | 0.63 | 4.12 | 16.91 | 5.64 | 3.35 | 9.02 |
| 68 | 4-Methylthiazole | | Nut and tomato | 0.055 | n.d. | 0.37 | 0.27 | 0.38 | 0.23 | 3.80 | 1.73 |
| 69 | Pyrazine | | Sweet corn | 75 | n.d. | 0.001 | 0.00007 | 0.0002 | 0.0002 | 0.0009 | 0.0004 |
| 70 | dms | | Pungent | 0.001 | n.d. | 84.07 | 100.00 | 100.00 | 100.00 | 100.00 | 45.82 |
| 71 | 2,6-Dimethylpyrazine | | Cocoa, nut, and grilled meat | 0.4 | n.d. | 0.13 | 0.12 | 0.13 | 0.05 | 0.45 | 0.18 |
| 72 | 2-Isobutyl-3-methoxypyrazine | | Bell Pepper, Earth, Green Pepper, Spice | 0.01 | 0.64 | n.d. | n.d. | n.d. | n.d. | n.d. | n.d. |
| 73 | Limonene | | Lemon | 0.2 | n.d. | n.d. | n.d. | n.d. | n.d. | n.d. | n.d. |

**Table S4.** (continued)

| No. | Compounds | Odor characteristics | Threshold (mg/kg) | ROAV | | | | | | |
| --- | --- | --- | --- | --- | --- | --- | --- | --- | --- | --- |
|  |  |  |  | **Y1** | **P1** | **P2** | **P3** | **L1** | **L2** | **L3** |
| 74 | 2-Methylpyrazine | Nut,baked | 0.06 | n.d. | n.d. | n.d. | n.d. | n.d. | n.d. | n.d. |
| 75 | 1-(Furan-2-yl)ethanone | Almond, caramel coffee | 8 | n.d. | n.d. | n.d. | n.d. | n.d. | n.d. | n.d. |

^1^Odor characteristics were from FEMA database; ^2^aroma threshold values as reported in Van Gemert (2011)

**Table S5.** ROAV of VOCs in lipase-catalyzed alcoholized butter.

| No. | Compounds | Odor characteristics | Threshold (mg/kg) | ROAV | | | | | |
| --- | --- | --- | --- | --- | --- | --- | --- | --- | --- |
|  |  |  |  | **EP1** | **EP2** | **EP3** | **EL1** | **EL2** | **EL3** |
| 1 | Acetic acid | Acid, Fruit, Pungent, Sour, Vinegar | 22 | n.d. | n.d. | n.d. | n.d. | n.d. | n.d. |
| 2 | 1-Hexanoic acid | Coconut meat and cheese | 3 | n.d. | n.d. | n.d. | n.d. | n.d. | n.d. |
| 3 | Butyric Acid | Yogurt | 0.1 | 1.97 | 5.78 | 5.96 | 0.79 | 1.16 | 1.54 |
| 4 | Propionic acid | Acidic cheese, vinegar | 0.1 | 0.31 | 1.87 | 1.89 | 0.36 | 1.12 | 5.54 |
| 5 | 2-Methylbutyric acid | Acidic goat milk cheese | 0.01 | 8.12 | 8.93 | 11.44 | 9.66 | 8.14 | 5.41 |
| 6 | Valeraldehyde | Fermented bread and fruit | 0.012 | n.d. | n.d. | n.d. | n.d. | n.d. | n.d. |
| 7 | 2-Methylbutanal | Fermented and baked | 0.0024 | n.d. | n.d. | n.d. | n.d. | n.d. | n.d. |
| 8 | Butanal | Pungent | 0.009 | n.d. | n.d. | n.d. | n.d. | n.d. | n.d. |
| 9 | Isovaleraldehyde | Chocolate and peach | 0.0024 | n.d. | n.d. | n.d. | n.d. | n.d. | n.d. |
| 10 | 2-Methylpropenal | Wild hyacinth | 0.025 | n.d. | n.d. | n.d. | n.d. | n.d. | n.d. |
| 11 | Hexanal | Oil and grass | 0.0045 | 31.77 | 25.27 | 23.52 | 1.25 | 1.11 | 1.12 |
| 12 | Propanal | Soil and nut | 0.0151 | n.d. | n.d. | n.d. | n.d. | n.d. | n.d. |
| 13 | Acetaldehyde | Floral, Green Apple | 0.025 | n.d. | n.d. | n.d. | n.d. | n.d. | n.d. |
| 14 | Nonanal | Fat, Floral, Green, Lemon | 0.001 | n.d. | n.d. | n.d. | n.d. | n.d. | n.d. |
| 15 | Isobutyraldehyde | Burnt, Caramel, Cocoa, Green, Malt | 0.0004 | n.d. | n.d. | n.d. | n.d. | n.d. | n.d. |
| 16 | Heptanal | Citrus, Fat, Green, Nut | 0.003 | 21.71 | 23.24 | 29.10 | 2.89 | 2.57 | 1.56 |
| 17 | Octanal | Fat, sweet orange | 0.0034 | 20.14 | 28.69 | 32.62 | 1.99 | 1.98 | 1.16 |
| 18 | Furfural | Almonds, toasted bread | 3 | 0.01 | 0.02 | 0.04 | 0.01 | 0.02 | 0.04 |
| 19 | N-Methylpyrrole-2-carboxaldehyde | roasted nuts | 0.037 | 2.28 | 2.67 | 3.05 | 3.71 | 4.47 | 5.21 |
| 20 | 2-Methylpentanal | Green, Fruit | 0.0016 | n.d. | n.d. | n.d. | n.d. | n.d. | n.d. |
| 21 | 2-Methylbutan-1-ol | Alcohol | 0.25 | n.d. | n.d. | n.d. | n.d. | n.d. | n.d. |
| 22 | Ethanol | Alcohol | 0.025 | n.d. | n.d. | n.d. | n.d. | n.d. | n.d. |
| 23 | 1-Propanol | Alcohol | 5.7 | n.d. | n.d. | n.d. | n.d. | n.d. | n.d. |
| 24 | 3-Methyl-1-butanol | Slight alcohol | 0.25 | n.d. | n.d. | n.d. | n.d. | n.d. | n.d. |
| 25 | Isoprenol | Sweet fruit and wine | 0.547125 | n.d. | n.d. | n.d. | n.d. | n.d. | n.d. |
| 26 | Methanethiol | Garlic | 0.004 | n.d. | n.d. | n.d. | n.d. | n.d. | n.d. |
| 27 | 1,3-Butanediol | Bitter-sweet | 10 | n.d. | n.d. | n.d. | n.d. | n.d. | n.d. |
| 28 | 2-Ethylhexanol | Green, Rose | 0.83 | n.d. | n.d. | n.d. | n.d. | n.d. | n.d. |
| 29 | Isopentanol | Burnt, Cocoa, Floral, Malt | 0.25 | n.d. | n.d. | n.d. | n.d. | n.d. | n.d. |
| 30 | 2-Heptanone (M) | Fruit, herbal and wood | 0.14 | n.d. | n.d. | n.d. | n.d. | n.d. | n.d. |
| 31 | 2-Heptanone (D) | Fruit, herbal and wood | 0.14 | n.d. | n.d. | n.d. | n.d. | n.d. | n.d. |
| 32 | 2-Pentanone(M) | Sweet fruit, wine and woody | 1.38 | n.d. | n.d. | n.d. | n.d. | n.d. | n.d. |
| 33 | 2-Pentanone(D) | Sweet fruit, wine and wood | 1.38 | 0.02 | 0.03 | 0.08 | 0.02 | 0.06 | 0.09 |
| 34 | 2, 3-Pentanedione (M) | Butter and cream | 0.02 | n.d. | n.d. | n.d. | n.d. | n.d. | n.d. |
| 35 | 2, 3-Pentanedione (D) | Butter and cream | 0.02 | n.d. | n.d. | n.d. | n.d. | n.d. | n.d. |
| 36 | 2-Butanone | Fragrant and fruit | 17 | n.d. | n.d. | n.d. | n.d. | n.d. | n.d. |
| 37 | 1-Penten-3-one | Spicy pepper | 0.023 | n.d. | n.d. | n.d. | n.d. | n.d. | n.d. |
| 38 | 2-Octanone | Natural woody and herbal | 0.005 | n.d. | n.d. | n.d. | n.d. | n.d. | n.d. |
| 39 | 3-Hexanone | Sweet fruit and wine | 0.041 | n.d. | n.d. | n.d. | n.d. | n.d. | n.d. |

**Table S5.** (continued)

| No. | Compounds | Odor characteristics | Threshold (mg/kg) | ROAV | | | | | |
| --- | --- | --- | --- | --- | --- | --- | --- | --- | --- |
|  |  |  |  | **EP1** | **EP2** | **EP3** | **EL1** | **EL2** | **EL3** |
| 40 | Acetoin | Butter | 8 | n.d. | n.d. | n.d. | n.d. | n.d. | n.d. |
| 41 | 3-Penten-2-one | Acetone | 0.0015 | n.d. | n.d. | n.d. | n.d. | n.d. | n.d. |
| 42 | 2,3-Butanedione | Rich butter and sweet cream | 0.0065 | 100.00 | 100.00 | 100.00 | n.d. | n.d. | n.d. |
| 43 | Hydroxyacetone | Butter, Herb, Malt, Pungent | 80 | n.d. | n.d. | n.d. | n.d. | n.d. | n.d. |
| 44 | 4-Methyl-2-pentanone | green | 0.24 | n.d. | n.d. | n.d. | n.d. | n.d. | n.d. |
| 45 | Ethyl (methylthio)acetate | Fruit | 0.025 | 3.70 | 3.46 | 17.95 | 19.30 | 20.01 | 32.85 |
| 46 | Ethyl butyrate | Fruit | 0.066 | 26.96 | 28.86 | 29.42 | 26.09 | 34.95 | 42.23 |
| 47 | n-Ethyl propanoate | sweet fruits | 0.01 | 61.95 | 57.86 | 82.58 | 100.00 | 100.00 | 100.00 |
| 48 | Ethyl acetate(M) | Fruit | 5 | n.d. | n.d. | n.d. | n.d. | n.d. | n.d. |
| 49 | Ethyl acetate(D) | Fruit | 5 | n.d. | n.d. | n.d. | n.d. | n.d. | n.d. |
| 50 | Isoamyl N-butyrate | Fruit | 0.015 | n.d. | n.d. | n.d. | n.d. | n.d. | n.d. |
| 51 | Butyl acetate | Apple or pineapple | 0.001 | n.d. | n.d. | n.d. | n.d. | n.d. | n.d. |
| 52 | Pentyl acetate | Banana | 0.0041 | n.d. | n.d. | n.d. | n.d. | n.d. | n.d. |
| 53 | Methyl acetate | Sweet | 1.5 | n.d. | n.d. | n.d. | n.d. | n.d. | n.d. |
| 54 | Propyl acetate(M) | Celery, Floral, Pear, Red Fruit | 2.7 | n.d. | n.d. | n.d. | n.d. | n.d. | n.d. |
| 55 | Propyl acetate(D) | Celery, Floral, Pear, Red Fruit | 2.7 | n.d. | n.d. | n.d. | n.d. | n.d. | n.d. |
| 56 | Ethyl acrylate | Plastic, Fragrant, Pungent | 0.0067 | n.d. | n.d. | n.d. | n.d. | n.d. | n.d. |
| 57 | Pentyl butanoate | Banana,fruity | 0.21 | n.d. | n.d. | n.d. | n.d. | n.d. | n.d. |
| 58 | Ethyl formate | Pungent | 8.9 | n.d. | n.d. | n.d. | n.d. | n.d. | n.d. |
| 59 | Ethyl propanoate | Apple, Pineapple, Rum, Strawberry | 0.01 | n.d. | n.d. | n.d. | n.d. | n.d. | n.d. |
| 60 | Isobutyl acetate | Apple, Banana, Floral, Herb | 0.44 | n.d. | n.d. | n.d. | n.d. | n.d. | n.d. |
| 61 | Ethyl isobutyrate | fruity, wine | 0.0001 | n.d. | n.d. | n.d. | n.d. | n.d. | n.d. |
| 62 | Isopropyl acetate | Banana | 1.7 | n.d. | n.d. | n.d. | n.d. | n.d. | n.d. |
| 63 | Myrcene | Balsamic, Fruit, Geranium, Herb, Must | 0.015 | n.d. | n.d. | n.d. | n.d. | n.d. | n.d. |
| 64 | *α*-Phellandrene | Citrus | 0.04 | n.d. | n.d. | n.d. | n.d. | n.d. | n.d. |
| 65 | *β*-Pinene | Eucalyptus camphor | 0.14 | n.d. | n.d. | n.d. | n.d. | n.d. | n.d. |
| 66 | 1-Octene | Gasoline smell | 0.0005 | n.d. | n.d. | n.d. | n.d. | n.d. | n.d. |
| 67 | 2-Isobutyl-3-methylpyrazine | Syrup | 0.035 | n.d. | n.d. | n.d. | n.d. | n.d. | n.d. |
| 68 | 4-Methylthiazole | Nut and tomato | 0.055 | n.d. | n.d. | n.d. | n.d. | n.d. | n.d. |
| 69 | Pyrazine | Sweet corn | 75 | n.d. | n.d. | n.d. | n.d. | n.d. | n.d. |
| 70 | dms | Pungent | 0.001 | n.d. | n.d. | n.d. | n.d. | n.d. | n.d. |
| 71 | 2,6-Dimethylpyrazine | Cocoa, nut, and grilled meat | 0.4 | n.d. | n.d. | n.d. | n.d. | n.d. | n.d. |
| 72 | 2-Isobutyl-3-methoxypyrazine | Bell Pepper, Earth, Green Pepper, Spice | 0.01 | n.d. | n.d. | n.d. | n.d. | n.d. | n.d. |

**Table S5.** (continued)

| No. | Compounds | Odor characteristics | Threshold (mg/kg) | ROAV | | | | | |
| --- | --- | --- | --- | --- | --- | --- | --- | --- | --- |
|  |  |  |  | **EP1** | **EP2** | **EP3** | **EL1** | **EL2** | **EL3** |
| 73 | Limonene | Lemon | 0.2 | 0.78 | 2.74 | 2.74 | 0.47 | 1.73 | 4.80 |
| 74 | 2-Methylpyrazine | Nut,baked | 0.06 | 22.45 | 55.66 | 58.00 | 29.35 | 47.41 | 80.26 |
| 75 | 1-(Furan-2-yl)ethanone | Almond, caramel coffee | 8 | 0.003 | 0.003 | 0.01 | 0.01 | 0.02 | 0.03 |

**Table S6.** SFC curves of raw, lipase-catalyzed hydrolyzed and lipase-catalyzed alcoholized butter.

**Table S6 (A).**

| **Temperature (℃)** | **SFC (%)** | | | |  | **Temperature (℃)** | **SFC (%)** | | | |
| --- | --- | --- | --- | --- | --- | --- | --- | --- | --- | --- |
|  | **Y1** | **P1** | **P2** | **P3** |  |  | **Y1** | **EP1** | **EP2** | **EP3** |
| 0 | 49.84±3.82^b^ | 56.68±1.97^a^ | 54.72±0.01^ab^ | 50.12±0.22^b^ |  | 0 | 49.84±3.82^a^ | 50.04±0.18^a^ | 50.50±1.10^a^ | 55.14±1.11^a^ |
| 10 | 38.34±3.90^ab^ | 43.47±2.35^a^ | 40.12±0.64^ab^ | 36.41±0.38^b^ |  | 10 | 38.34±3.90^ab^ | 36.46±0.84^b^ | 36.69±0.58^b^ | 43.25±1.31^a^ |
| 20 | 21.56±2.70^a^ | 9.87±1.07^b^ | 8.43±0.60^b^ | 6.69±0.08^b^ |  | 20 | 21.56±2.70^a^ | 7.49±0.01^c^ | 7.91±0.001^c^ | 11.60±0.01^b^ |
| 25 | 9.95±1.53^a^ | 2.50±0.22^b^ | 1.43±0.18^bc^ | n.d. |  | 25 | 9.95±1.53^a^ | 0.38±0.10^c^ | 0.72±0.33^c^ | 3.87±0.19^b^ |
| 30 | 2.73±0.91^a^ | n.d. | n.d. | n.d. |  | 30 | 2.73±0.91^a^ | n.d. | n.d. | n.d. |
| 35 | 0.60±0.47^a^ | n.d. | n.d. | n.d. |  | 35 | 0.60±0.47^a^ | n.d. | n.d. | n.d. |
|  |  |  |  |  |  |  |  |  |  |  |
| **Temperature (℃)** | **SFC (%)** | | | |  | **Temperature (℃)** | **SFC (%)** | | | |
|  | **Y1** | **L1** | **L2** | **L3** |  |  | **Y1** | **EL1** | **EL2** | **EL3** |
| 0 | 49.84±3.82^b^ | 63.07±0.01^a^ | 62.80±0.00^a^ | 62.23±0.14^a^ |  | 0 | 49.84±3.82^b^ | 56.34±0.16^ab^ | 58.09±0.01^a^ | 58.09±0.24^a^ |
| 10 | 38.34±3.90^b^ | 50.87±0.19^a^ | 50.32±0.76^a^ | 50.05±0.23^a^ |  | 10 | 38.34±3.90^b^ | 44.30±0.23^ab^ | 45.86±0.03^ab^ | 46.62±0.37^a^ |
| 20 | 21.56±2.70^b^ | 16.73±0.14^a^ | 16.44±0.42^a^ | 16.04±1.26^a^ |  | 20 | 21.56±2.70^a^ | 13.47±0.23^b^ | 14.24±0.05^b^ | 14.87±0.11^b^ |
| 25 | 9.95±1.53^a^ | 8.35±0.03^a^ | 8.15±0.15^a^ | 8.03±0.71^a^ |  | 25 | 9.95±1.53^a^ | 5.61±0.02^b^ | 6.40±0.02^b^ | 6.61±0.06^b^ |
| 30 | 2.73±0.91^a^ | 1.70±0.05^ab^ | 1.43±0.16^b^ | 1.17±0.18^b^ |  | 30 | 2.73±0.91^a^ | n.d. | 0.17±0.14^b^ | 0.42±0.34^b^ |
| 35 | 0.60±0.47^a^ | n.d. | n.d. | n.d. |  | 35 | 0.60±0.47^a^ | n.d. | n.d. | n.d. |

**Table S6 (B).**

| **Temperature (℃)** | **SFC (%)** | |  | **SFC (%)** | |  | **SFC (%)** | |  |
| --- | --- | --- | --- | --- | --- | --- | --- | --- | --- |
|  | **P1** | **EP1** |  | **P2** | **EP2** |  | **P3** | **EP3** |  |
| 0 | 56.68±1.97 | 50.04±0.18 | * | 54.72±0.01 | 50.50±1.10 | * | 50.12±0.22 | 55.14±1.11 | * |
| 10 | 43.47±2.35 | 36.46±0.84 | * | 40.12±0.64 | 36.69±0.58 | ** | 36.41±0.38 | 43.25±1.31 | ** |
| 20 | 9.87±1.07 | 7.49±0.01 | n.s. | 8.43±0.60 | 7.91±0.001 | n.s. | 6.69±0.08 | 11.60±0.01 | *** |
| 25 | 2.50±0.22 | 0.38±0.10 | *** | 1.43±0.18 | 0.72±0.33 | * | n.d. | 3.87±0.19 | *** |
| 30 | n.d. | n.d. |  | n.d. | n.d. |  | n.d. | n.d. |  |
| 35 | n.d. | n.d. |  | n.d. | n.d. |  | n.d. | n.d. |  |
|  |  |  |  |  |  |  |  |  |  |
| **Temperature (℃)** | **SFC (%)** | |  | **SFC (%)** | |  | **SFC (%)** | |  |
|  | **L1** | **EL1** |  | **L2** | **EL2** |  | **L3** | **EL3** |  |
| 0 | 63.07±0.01 | 56.34±0.16 | *** | 62.80±0.00 | 58.09±0.01 | *** | 62.23±0.14 | 58.09±0.24 | *** |
| 10 | 50.87±0.19 | 44.30±0.23 | *** | 50.32±0.76 | 45.86±0.03 | ** | 50.05±0.23 | 46.62±0.37 | *** |
| 20 | 16.73±0.14 | 13.47±0.23 | *** | 16.44±0.42 | 14.24±0.05 | * | 16.04±1.26 | 14.87±0.11 | n.s. |
| 25 | 8.35±0.03 | 5.61±0.02 | *** | 8.15±0.15 | 6.40±0.02 | ** | 8.03±0.71 | 6.61±0.06 | n.s. |
| 30 | 1.70±0.05 | n.d. | *** | 1.43±0.16 | 0.17±0.14 | *** | 1.17±0.18 | 0.42±0.34 | * |
| 35 | n.d. | n.d. |  | n.d. | n.d. |  | n.d. | n.d. |  |

The results were expressed as mean ± standard deviation (n = 3). Mean values with different lowercase letters in the same row are significantly different at *p* < 0.05. *, *p* < 0.05; **, *p* < 0.01; ***, *p* < 0.001; n.s., not significant.

**Reference:**

Van Gemert, L. J. (2011). Compilations of odour threshold values in air, water and other media (2th ed.). Science. <https://doi.org/10.1016/0048-9697(78)90024-4>
